# Discovery of a First-In-Class Small Molecule Antagonist against the Adrenomedullin-2 Receptor: Structure–Activity Relationships and Optimization

**DOI:** 10.1021/acs.jmedchem.0c02191

**Published:** 2021-03-05

**Authors:** Jean-Olivier Zirimwabagabo, Ameera B. A. Jailani, Paris Avgoustou, Matthew J. Tozer, Karl R. Gibson, Paul A. Glossop, James E. J. Mills, Roderick A. Porter, Paul Blaney, Ning Wang, Timothy M. Skerry, Gareth O. Richards, Joseph P. A. Harrity

**Affiliations:** † Department of Chemistry, University of Sheffield, Sheffield S10 2TN, U.K.; ‡ Department of Oncology and Metabolism, University of Sheffield, Sheffield S10 2TN, U.K.; § Matt Tozer Consultancy, Bognor Regis PO21 1DY, U.K.; ∥ Sandexis Medicinal Chemistry Ltd., Sandwich, Kent CT13 9ND, U.K.; ⊥ Rod Porter Consultancy, Ashwell, Hertfordshire SG7 5PG, U.K.; ¶ Concept Life Sciences, High Peak SK23 0PG, U.K.

## Abstract

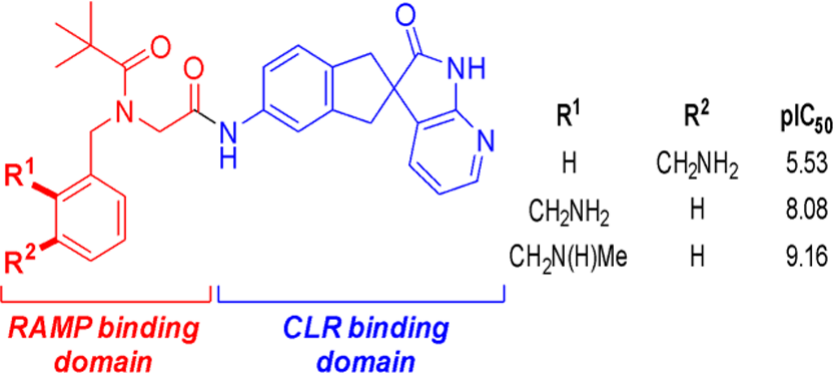

Class B G-protein-coupled receptors (GPCRs) remain an underexploited target for drug development. The calcitonin receptor (CTR) family is particularly challenging, as its receptors are heteromers comprising two distinct components: the calcitonin receptor-like receptor (CLR) or calcitonin receptor (CTR) together with one of three accessory proteins known as receptor activity-modifying proteins (RAMPs). CLR/RAMP1 forms a CGRP receptor, CLR/RAMP2 forms an adrenomedullin-1 (AM_1_) receptor, and CLR/RAMP3 forms an adrenomedullin-2 (AM_2_) receptor. The CTR/RAMP complexes form three distinct amylin receptors. While the selective blockade of AM_2_ receptors would be therapeutically valuable, inhibition of AM_1_ receptors would cause clinically unacceptable increased blood pressure. We report here a systematic study of structure–activity relationships that has led to the development of first-in-class AM_2_ receptor antagonists. These compounds exhibit therapeutically valuable properties with 1000-fold selectivity over the AM_1_ receptor. These results highlight the therapeutic potential of AM_2_ antagonists.

## Introduction

G-protein-coupled receptors (GPCRs) are the largest family of cell surface receptors, with a broad range of physiological and pathophysiological roles.^1^ GPCRs have been promising and successful targets for many therapeutic interventions.^2^ Functional complexity and pharmacological diversity of GPCRs can be further influenced by interactions with receptor activity-modifying proteins (RAMPs). RAMPs are a family of single transmembrane domain proteins that complex with GPCRs to facilitate cell surface trafficking, receptor pharmacology as well as recycling and degradation.^3,4^ Of the six classes of GPCRs, members of class B (secretin receptor family) have been most studied for their interactions with RAMPs and include calcitonin (CTR) and calcitonin receptor-like (CLR) receptors.^4,5^ Despite their physiological importance and promising therapeutic potential, the small number of full-length ligand-bound structures of class B GPCRs and the limited structural information on druggable binding sites have made the development of compounds that target this GPCR family challenging.^6,7^ However, a number of structures have been solved recently^8−10^ due to advances in cryo-EM technology and resolution, so that further developments are now more feasible. Regardless, a number of compounds have been reported in the past decade, including synthetic modulators of glucagon, glucagon-like peptide-1, corticotropin-releasing factor 1, and calcitonin receptor-like receptors.^11−13^ The most successful target of class B GPCRs for small molecule modulators has been the CGRP receptor (comprising CLR and RAMP1) for which several antagonists and antibodies have been developed in recent years for the treatment of migraine.^14−18^ Some of these have reached the market including the two oral small molecule antagonists, rimegepant^19^ (Nurtec ODT) and ubrogepant^20^ (Ubrelvy), as well as the three injectable signal blocking monoclonal antibodies, erenumab^21^ (Aimovig), eptinezumab^22^ (Vyepti), and galcanezumab^23^ (Emgality). For small molecule antagonists, the binding site has been shown by X-ray crystallography studies to be at the interface between RAMP1 and the CLR.^24^

The selectivity of CGRP receptor antagonists indicates the potential of exploiting differences between CLR/RAMP receptor complexes to develop antagonists for other members of the CLR family, such as receptors of the hormone adrenomedullin (AM). While the CGRP receptor comprises CLR and RAMP1, adrenomedullin-1 (AM_1_) and adrenomedullin-2 (AM_2_) receptors form by the interaction of CLR with RAMP2 and RAMP3, respectively.^4^ AM is a potent vasodilator that regulates blood pressure.^25^ While AM signaling through the AM_1_ receptor is required for cardiovascular homeostasis,^26^ aberrant AM signaling is implicated in cancer development and progression.^27,28^ Both AM and the AM_2_ receptors have been shown to be upregulated and mediate protumoral processes in many cancers,^29−31^ including breast and pancreatic cancers.^32,33^

We have recently reported the discovery of the first-in-class small molecule antagonists against the AM_2_ receptor.^34^ These molecules are important new tools that will provide significant insight into the pharmacology of the CLR/RAMP receptor family. Additionally, they show promising antitumoral effects in both *in vitro* and *in vivo* models of pancreatic cancer. With a view to therapeutic potential, the new AM_2_ receptor antagonists show 1000-fold selectivity against the AM_1_ receptor, enabling physiological signaling of AM to continue through AM_1_ receptors, lowering the risk of off-target side effects mediated by the AM_1_ receptor.

Here, we describe the development and structure–activity relationships (SARs) of this family of small molecule antagonists. The chemistry strategy is underpinned by simple and convergent synthesis routes, and the efficacy of these compounds was evaluated in *in vitro* and *in vivo* models of breast cancer. The exploration of full drug-like characteristics (ADME, PK, and *in vitro* safety markers) of lead compounds is described by Avgoustou et al.^34^

## Results and Discussion

### Design and SAR

There are four significant differences between RAMP1 and RAMP3 in the vicinity of the small molecule ligand-binding pocket, namely, R67E, A70T, D71N, and W74E.^34^ Of these, we chose W74E as a residue difference to exploit because of its interaction with ligands that have been crystallized in the CGRP receptor. The incorporation of a basic center to interact with the glutamate carboxylate provided a compelling strategy for designing AM_2_ receptor-selective ligands. The W74E change is also seen when comparing RAMP1 with RAMP2; therefore, the simplest approach to building a pseudo (hybrid)-model of the AM_2_ receptor-binding pocket was to transpose the side-chain conformation of Glu105 from the RAMP2 crystal structure (PDB code 3AQF(35)) into the Trp74 position of the CGRP receptor crystal structure (PDB code 3N7R(36)). Alternative conformations of the glutamate side chain were examined but the results were not significantly affected. As all our compounds were active, at least to some extent at both CGRP and AM_2_ receptors, it was decided to use this slightly modified structure of the CGRP receptor as a basis for docking, making the assumption that the binding modes in both CGRP and AM_2_ receptors would be the same.^37^

The crystal structure of MK-3207 has been solved,^38^ but the only information published is a figure that shows a stick representation of the ligand and a surface representation of the protein. The ligand was docked such that it replicated as much of the information presented in this image as possible. Conformations of ligands were initially built using Open Babel (version 2.3.1). The starting conformation of the CLR-binding spiro ring system was fixed to replicate the configuration observed in the image, and the resultant conformer was refined by density functional theory (DFT) minimization in ORCA.^39^ Docking was carried out using GOLD,^40^ tethering the lactam or equivalent portion of the headgroup onto that observed in the PDB structure of telcagepant bound to the ectodomain of the CGRP receptor (PDB code 3N7R(36)) and generating 30 docks per compound using default options. Results were processed using an in-house script to cluster the docks and assess the quality of hydrogen bonds, identifying docks with high GOLD scores, no antihydrogen bonds, and, where relevant, a high-quality interaction with the glutamate (distance between heavy atoms of 2.7–3.5 Å and the angle subtended at donor H of close to 180°).

**Figure 1:**
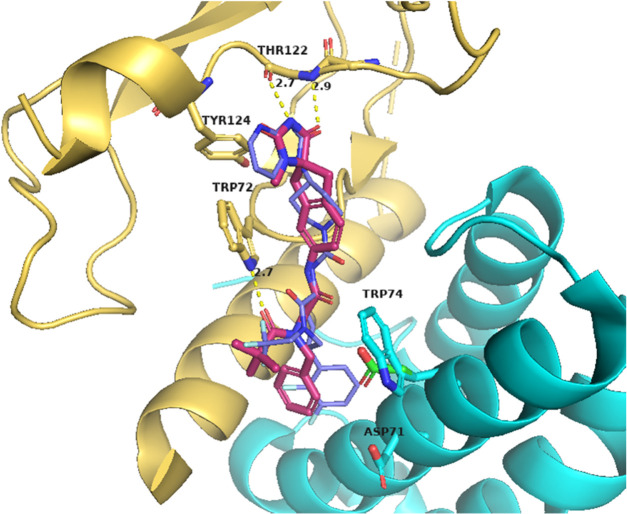
Docking of telcagepant (magenta) and compound **1** (purple) in our pseudo (hybrid)-model of the AM_2_ receptor-binding pocket. The Glu74 residue from RAMP3 is indicated in green. CLR is shown in yellow and RAMP3 in cyan. Hydrogen bonds are shown as dotted lines. Compound **1** has similar spatial occupancy and interactions to telcagepant (magenta) as observed in the CGRP receptor crystal structure (PDB code 3N7R(36)).

The dock of the published structure of compound **1**, a CGRP receptor antagonist with an encouraging activity against the AM_2_ receptor,^41^ overlaid well with telcagepant, preserving the interactions of the tethered headgroup (Figure 1). In addition, the carbonyl oxygen atom of the pivalamide substituent formed a hydrogen bond with the indole NH of CLR Trp72. The residue implicated in selectivity, W74E, appeared to be accessible from the ortho position of the phenyl ring, suggesting a position to introduce basic substituents (Figure 1).

**Figure 2:**
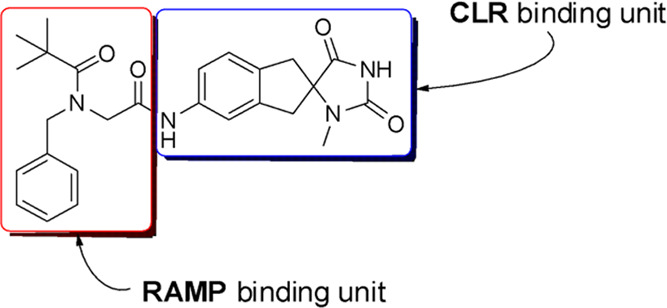
Compound **1** structure analysis for SAR.

From starting point compound **1**, the aim was to design and synthesize compounds that would bind strongly to the AM_2_ receptor. Our strategy assumed that modulating the RAMP structural binding fragment, while conserving the CLR-binding fragment, would lead to the successful identification of RAMP3-binding groups (Figure 2).

We first set out to investigate the antagonist behavior of several known CLR-binding fragments attached to the RAMP-binding motif present in **1**. As shown in Table 1, combining a set of known CLR fragments from CGRP inhibitors (specifically, telcagepant/rimegepant, MK-3207, and olcegepant) with the RAMP-binding portion of **1** led to analogues **2**–**4**, of which indene **3** showed moderately improved inhibition at the AM_2_ receptor (pIC_50_ = 6.7) relative to the initial indene lead **1**, with non-indenes losing measurable activity.

**Table 1:**
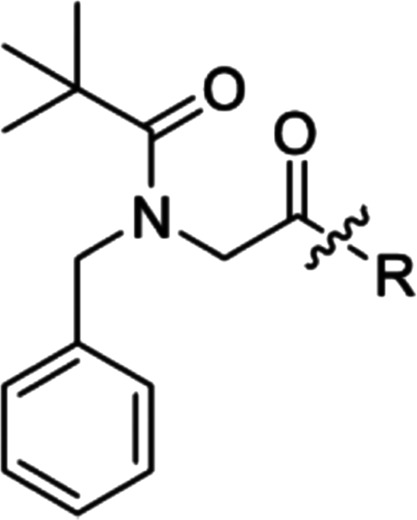

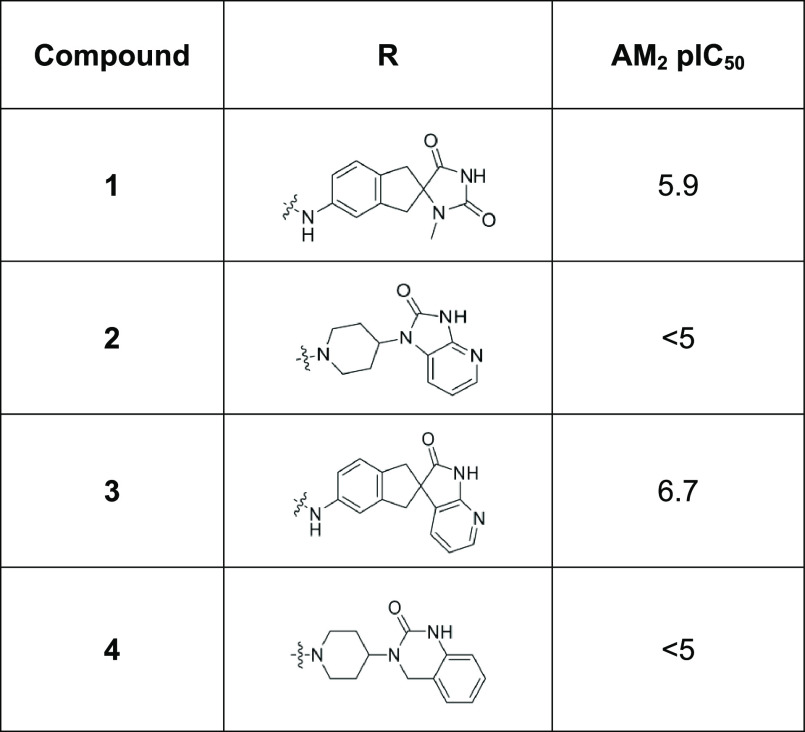
Investigating the Effect of Different CLR-Binding Fragments on the Antagonism against the AM_2_ Receptor

Working on the basis that the CLR fragment in **3** was optimal for our purposes, we implemented the strategy of incorporating a basic moiety into the benzyl side chain of the RAMP fragment. The pIC_50_ data of our compounds against AM_2_, together with data for selected compounds against AM_1_ and CGRP, are summarized in Table 2. Compounds bearing a heterocyclic or heteroaromatic ring showed reduced potency (compounds **5**–**8**), while the activity was broadly maintained when a pyrrolidine-substituted benzyl group or an indazole was incorporated (compounds **9**, **10**). Pleasingly, aniline **11** provided our first significant increase in receptor affinity, with a pIC_50_ of 8.2. Activity was maintained when the aniline was changed to a benzylic amine (compound **12**) but changing to the corresponding benzylic alcohol or moving the aminomethyl group to the meta-position led to a marked decrease in activity (compounds **13**, **14**). Returning to the positional scanning at the *ortho*-benzyl position, we were interested in finding that a primary amide, homologated primary amine, and tethered secondary amine produced pIC_50_ values of around 7 (compounds **15**–**17**). In contrast, benzylic morpholine, imidazole, pyridine, and nitrile groups performed poorly (compounds **18**–**21**), as did the aminomethyl analogue attached to a pyridine ring (compound **22**). The incorporation of further basic residues via an imidazole (compound **23**) or by changing to a guanidine moiety (compound **24**) failed to improve potency. However, simply homologating the aminomethyl group to secondary amine **23** gave a dramatic increase in affinity, providing our first inhibitor in the subnanomolar range (compound **25**). Further efforts to increase activity by increasing alkylation at various points around the benzylic aminomethyl fragment did not result in a significant enhancement of activity (compounds **25**–**29**). Overall, this study highlighted the importance of the spatial orientation of the basic group (e.g., compound **12** versus compound **14**) and the sensitivity of the receptor to steric bulk in the RAMP3-binding region (cf. compounds **25**, **27**, **18**). Finally, in all cases where compounds were cross-screened against AM_1_ receptor, very weak potency was observed. Although RAMP2 contains Asp and Glu at the equivalent positions, it is believed that other significant differences in the pocket are responsible for the lack of activity at the AM_1_ receptor. For example, (i) residue 70 (Ala in RAMP1 and Thr in RAMP3) is the much larger Arg in RAMP2, which, in crystal structures (PDB code 4RWF(42)), sits in space that would clash with these ligands, and (ii) Trp84 in RAMP3, which makes significant interactions with the core of these ligands, is a Trp in RAMP3 but the smaller Phe in RAMP2, which is unable to contact the ligands and therefore leaving an energetically unfavorable void.

**Table 2:**
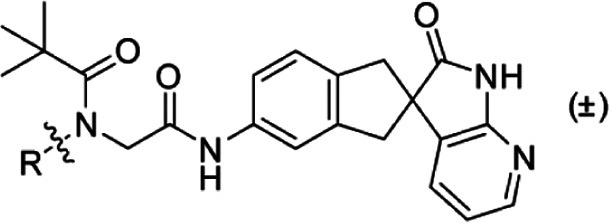

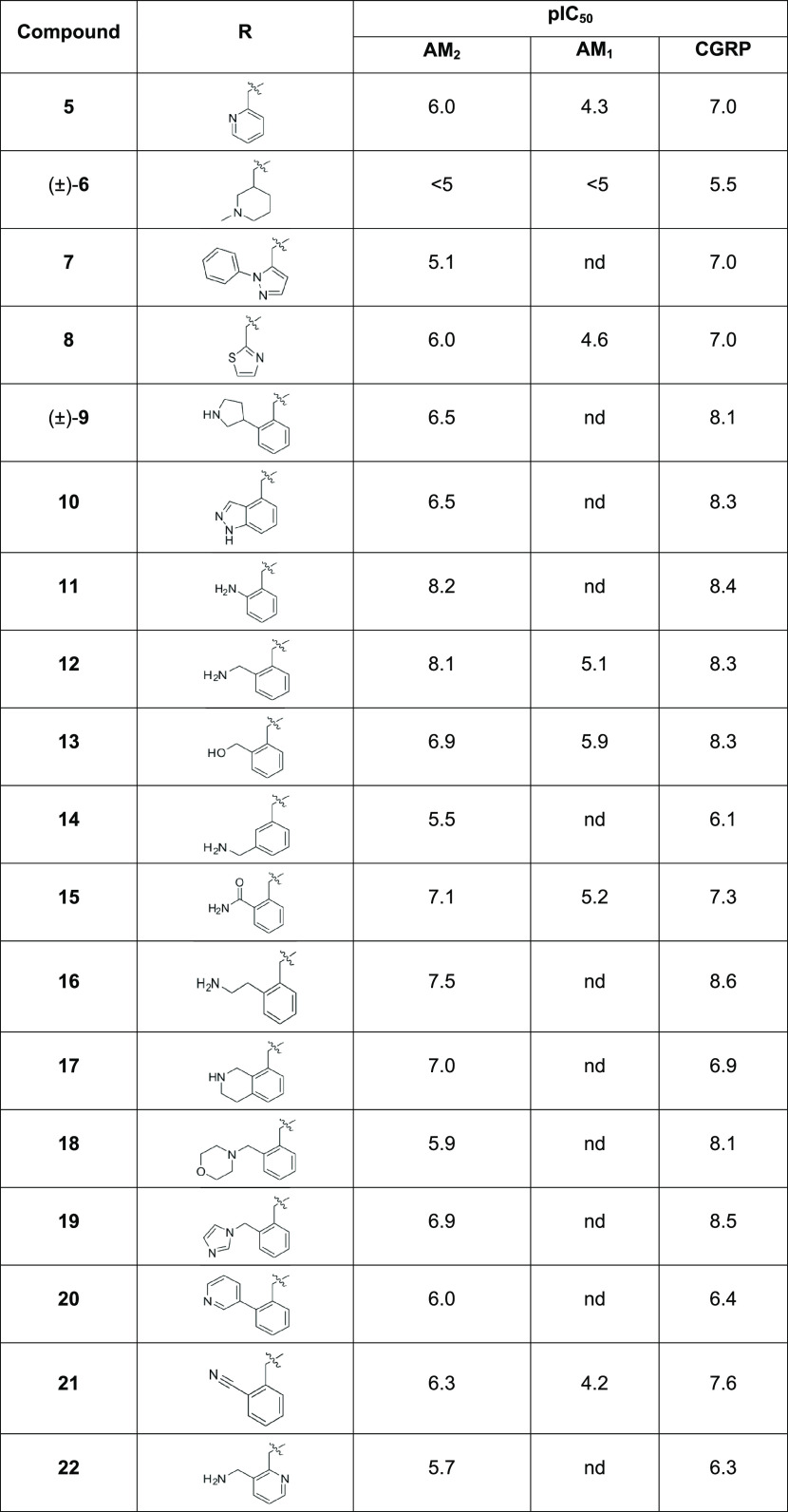

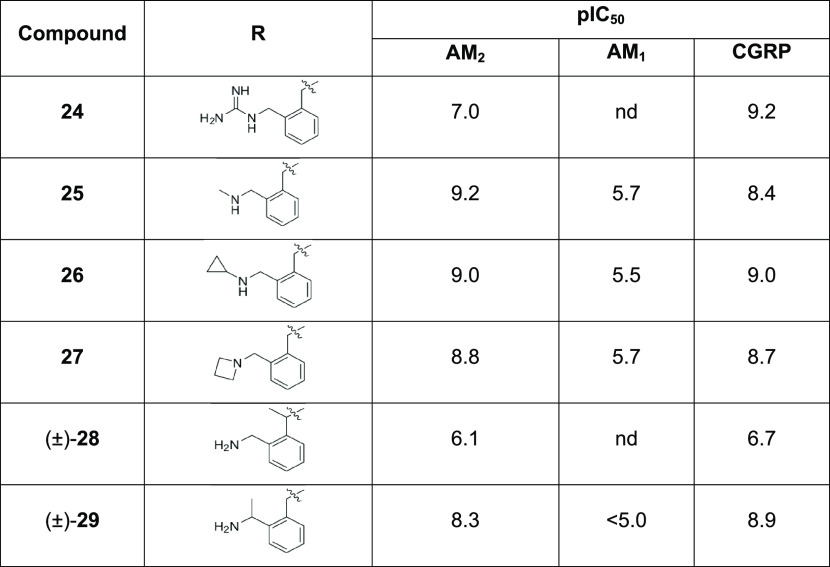
pIC_50_ Values of RAMP with *N*-Alkyl-Substituent SAR Library against the AM_2_ Receptor Compared to Those of AM_1_ and CGRP Receptors^a^

a nd: not determined.

## Chemistry

**Scheme 1:**
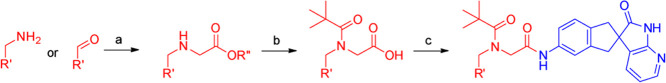
Reagents and General Conditions (a) Ethyl bromoacetate, *N*,*N*-diisopropylethylamine (DIPEA), dimethylformamide (DMF) or benzylbromoacetate, Et_3_N, tetrahydrofuran (THF; when amine was used) and glycine ethyl ester hydrochloride, NaBH_3_CN, MeOH (when aldehyde or ketone was used); (b) (i) PivCl, DIPEA, dichloromethane (DCM); (ii) 2.5 N NaOH, MeOH or LiOH·H_2_O, MeOH/THF/H_2_O; (c) HATU, NMM, DM or EDCI, HOAt, DIPEA, DMF.

The modular nature of our inhibitors offered simple and efficient synthetic routes from commercially available and inexpensive starting materials, allowing us rapidly to identify analogues with increased potency against the AM_2_ receptor. The general synthetic route is shown in Scheme 1. The reductive amination of the appropriate aldehyde with Ala-OMe or simple alkylation of amines with ethyl bromoacetate provided intermediates that were subjected to acylation with pivaloyl chloride and saponification to produce the desired RAMP-binding acids. Amide bond formation promoted by EDCI or HATU generated the final RAMP–CLR inhibitor constructs that could be further manipulated using standard functional group transformations (detailed synthetic procedures are described in the Supporting Information).

With a robust method in hand, we next wanted to assess the impact of the stereochemistry of the CLR-binding unit on activity as it is known to have an impact on the CGRP potency.^43,44^ For example, in the case of MK-3207, the analogue with a CLR-binding group in the (*S*) rather than (*R*) configuration reduces potency by 100-fold, from 0.12 to 10 nM. Indeed, as discussed in our preliminary report on this work,^34^ the (*R*)-enantiomer of **25** (isolated by the preparative chiral high-performance liquid chromatography (HPLC) of the racemate) was found to have improved potency over the corresponding (*S*)-enantiomer (pIC_50_ = 9.2 versus 7.2), so we set about devising an efficient synthesis of (*R*)-**25**.

**Scheme 2:**
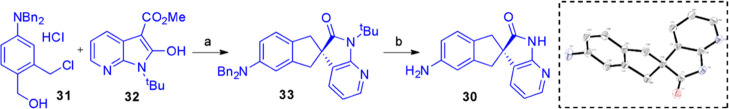
Reagents and Conditions (a) (i) NaOH, PTC*, toluene/H_2_O; (ii) recrystallized from toluene/MeOH; (b) (i) MsOH, toluene, 90 °C; (ii) ∼10% Pd/C, H_2_, HCl/MeOH, rt, o/n. PTC*: Chiral phase transfer catalyst. Please see Experimental Section for details.

The synthesis of the (*R*)-CLR-binding motif amine **30** was accomplished by a modification of the method reported by Bulger and Yasuda.^45,46^ An enantioselective phase transfer-catalyzed alkylation of **31** with **32** was found to give a higher degree of selectivity when excess sodium hydroxide was employed in toluene/H_2_O. This method allowed us to generate intermediate **33** in an ∼90% yield with 83% *ee* although this sample could be delivered in >99% *ee* after a single recrystallization (Scheme 2). Finally, the removal of the Bn- and ^*t*^Bu- groups gave the (*R*)-enantiomer of the desired amine **30** with >99% *ee*. Slow crystallization of **30** in methanol allowed us to confirm the product stereochemistry using single-crystal X-ray diffraction.

**Figure 3:**
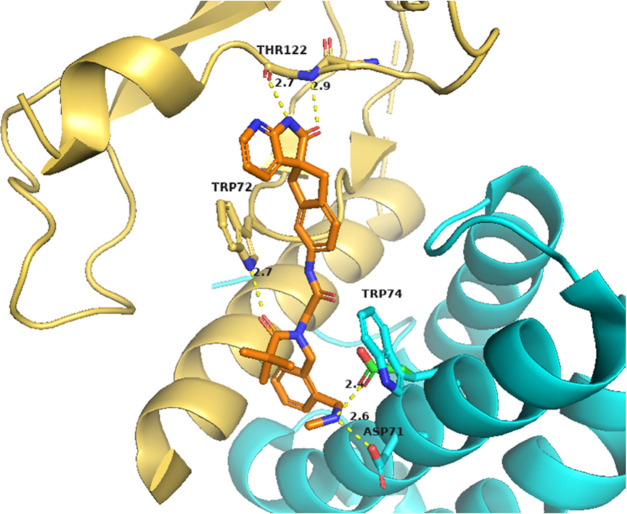
Docking of compound (*R*)-25 in our pseudo (hybrid)-model of the AM_2_ receptor-binding pocket. Glu74 residues from RAMP3 are indicated in green. CLR is shown in yellow and RAMP3 in cyan. Hydrogen bonds are shown as dotted lines. The protonated amine of compound (*R*)-25 forms salt bridges with the carboxylate of Glu74 and the carboxylate of Asp71 (an asparagine carboxamide in RAMP3).

The docked pose of (*R*)-**25** was consistent with that of **1** in the placement of their common substructures. As predicted, the protonated amine formed salt bridges with the carboxylate of Glu74 and the carboxylate of Asp71 (an asparagine carboxamide in RAMP3), possibly explaining why it is tolerated in both CGRP and AM_2_. The apparent preference for RAMP3 over RAMP1 could be explained by the interactions with the acid of Glu74 and the carbonyl of Asn71 being more favorable than the indole of Trp74 and the acid of Asp71, either because of differences in the salt bridge geometry or because the carboxamide is a preferred partner when compared with the indole (Figure 3).

### Antitumor Effect of (±)-**25**

**Figure 4:**
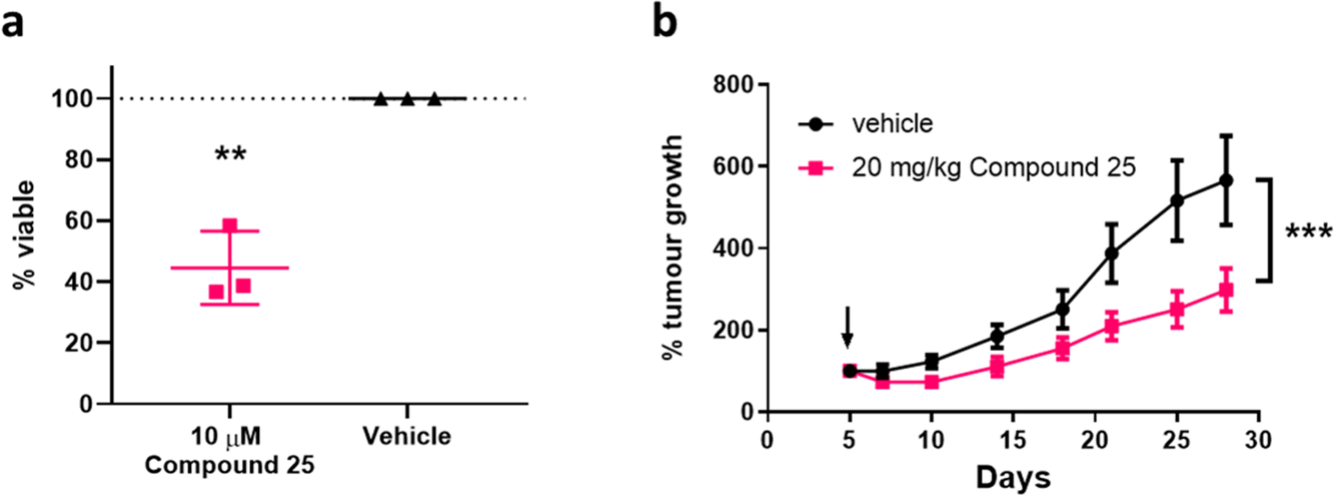
AM_2_ receptor antagonist inhibits *in vitro* viability of human breast cancer cell line MDA-MB-231 as well as subcutaneous MDA-MB-231 tumor growth in BALB/c nude mice. (a) Daily treatment with small molecule AM_2_ receptor antagonists significantly decreased the viability of MDA-MB-231 cells *in vitro* by 55% after 3 days when treated with 10 μM (±)-**25**, compared to that of vehicle-treated controls (*p* < 0.01, unpaired *t*-test). Data are from three independent experiments and presented as mean ± SD. (b) Mice (*n* = 10 per group) were inoculated subcutaneously with MDA-MB-231 cells to generate tumors, and first treatment was given on the day of the first tumor volume measurement (arrow). Tumor growth rates were significantly reduced in mice treated daily with 20 mg/kg ip (±)-**25** (*p* < 0.001, simple linear regression comparing the line of best fit). Data are presented as mean ± SD.

*In vitro* viability and *in vivo* subcutaneous xenograft models were used to determine antitumor effects of the AM_2_ receptor antagonist (±)-**25**, using the highly aggressive triple-negative breast cancer cell line MDA-MB-231. (±)-**25** decreased the MDA-MB-231 viability by 55% after 3 days of daily treatment at 10 μM concentration (Figure 4a; *p* < 0.01). For the *in vivo* xenograft study, MDA-MB-231 cells were subcutaneously inoculated under the skin of the flank of female BALB/c nude mice. Once the tumors were palpable (5 days after inoculation), (±)-**25** (20 mg/kg) or vehicle control was administered ip once daily. Tumors were measured twice weekly, and the well-being of mice was assessed by measuring the body weight and monitoring behavior. (±)-**25** was well tolerated, and the body weight of (±)-**25**-treated mice did not differ significantly when compared with that of vehicle-treated mice. No adverse effects were observed, and all mice exhibited apparently normal activity, feeding, and inquisitiveness. Daily administration of (±)-**25** (20 mg/kg) significantly decreased breast cancer xenograft tumor growth by 47% (Figure 4b; *p* < 0.001), 4 weeks after treatment.

## Conclusions

Here, we report a systematic and extensive structure–activity relationship study of our first-in-class AM_2_ receptor small molecule antagonists.^34^ Through the careful optimization of CLR and RAMP domain-binding fragments, we have been able to develop a family of antagonists that show high selectivity for AM_2_ over the closely related AM_1_ heteromer, by exploiting differences in the RAMP structures, focusing on residues 70 and 84. A robust chemistry strategy allowed us to prepare a large library of analogues and led to numerous derivatives with nanomolar potencies. In addition, the products are readily generated as single enantiomers through the employment of an efficient asymmetric synthesis of the (*R*)-CLR-binding unit. While our original goal was to identify compounds with selectivity for AM_2_ receptors over all of the CLR and CTR family receptors, we found it hard to separate AM_2_ and CGRP receptors in this respect. However, since CGRP receptors mediate pain, particularly bone pain in metastatic cancers, this may be an additional benefit for therapy in oncology. Finally, although we have previously shown the full drug-like properties (ADME, hERG, and PK) and selectivity profile of compound **25** and the effects of this compound class in pancreatic cancer cell viability and apoptosis in both *in vitro* and *in vivo* tumor growth models,^34^ we demonstrate here that similar potent antitumor effects are also observed in breast cancer models using the highly aggressive triple-negative breast cancer cell line MDA-MB-231.

## Experimental Section

All reagents, unless otherwise stated, were obtained from commercial sources and used without further purification. Small molecule antagonists were prepared as 2 mM dimethyl sulfoxide (DMSO) stocks for cell culture experiments and stored at −20 °C. Based on each cell line’s ligand–receptor combination, the appropriate unlabeled peptide was used. Human CGRP was obtained from Sigma-Aldrich (SCP0060), and human AM was purchased from Anaspec (AS-60447).

### Chemical Methods

**Scheme 3:**
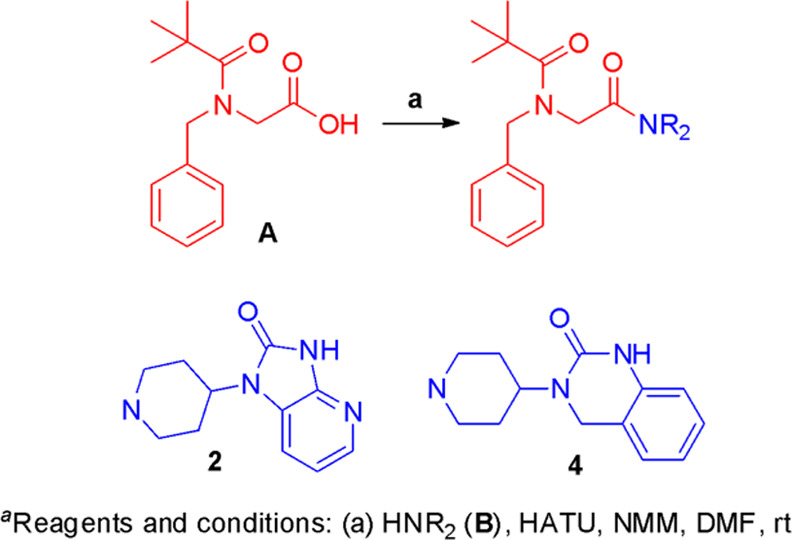
Synthesis of **2** and **4**

^1^H NMR spectra were recorded on a Bruker AVIII HD 400 (400 MHz), Bruker AVI 400 (400 MHz), Bruker AMX-400 (400 MHz), or DPX-400 (400 MHz) supported by an Aspect 3000 data system and referenced to the residual solvent peak (CDCl_3_: δ 7.26 ppm). Signal positions were recorded in δ ppm with the abbreviations s, d, t, q, br, and m denoting singlet, doublet, triplet, quartet, broad, and multiplet, respectively. ^19^F NMR spectra were recorded on a Bruker AVIII HD 400 (377 MHz) and are uncorrected. Flash chromatography was performed on silica gel (BDH Silica Gel 60 43-60 or Fluorochem Davisil silica gel 43-60) using head pressure by means of a compressed air line. Thin-layer chromatography (TLC) was performed on commercially available precoated aluminum-backed plates (Merck silica Kieselgel 60 F254). Spots were made visible either by the quenching of UV fluorescence or by staining with a potassium permanganate solution. All reactions were conducted in an oven or flame-dried glassware under an inert atmosphere of dry nitrogen or argon. Low-resolution mass spectra were (LC-MS) recorded on Micromass Autospec, operating in E.I., C.I., or FAB mode, or a PerkinElmer Turbomass Bench top GCMS operating in either E.I. or C.I. mode. All ultraperformance liquid chromatography-mass spectroscopy (UPLC-MS) analyses were carried out using Waters Acquity UPLC-MS (quaternary pump flow 0.8 mL/min, Acquity autosampler, PDA and QDA). All solvents, substrates, and reagents that were commercially available were used without further purification. Enantioselectivities were determined by high-performance liquid chromatography (HPLC) analysis employing a Gilson HPLC chain with an ABI Analytical Spectroflow 783 UV or an SPD-10 Shimadzu UV–vis detector. Purities of all final reported compounds were greater than 95% based on analytical HPLC chromatograms. Purification of the final compounds by preparative HPLC was accomplished on a C18 250 mm × 21 mm column in water/acetonitrile or with Biotage Isolera using a C18 Ultra cartridge in water/acetonitrile with pH = 10 buffer followed by freeze-drying of the pooled fractions containing pure products (Scheme 3).

#### *N*-Benzyl-*N*-(2-oxo-2-(4-(2-oxo-2,3-dihydro-1*H*-imidazo[4,5-*b*]pyridin-1-yl)piperidin-1-yl)ethyl)pivalamide (**2**)

**2A** (12 mg, 0.047 mmol), **2B** (13 mg, 0.052 mmol), and HATU (20 mg, 0.053 mmol) were dissolved in dry DMF (2 mL). *N*-Methylmorpholine (0.1 mL, 1.75 mmol) was added, and the mixture was stirred at rt for 5 min. The mixture was diluted with ethyl acetate and then washed with brine, dried over magnesium sulfate, filtered, and the filtrate was evaporated. The crude was purified using a Biotage Isolera (12 g, C18 Ultra cartridge, 60–80% acetonitrile/water with pH 10 buffer) and freeze-dried to provide **2** as a white solid (14.3 mg, 68%). ^1^H NMR (CD_3_OD, 300 MHz) δ 1.38 (s, 9H), 2.12 (br, 4H), 3.10–3.22 (m, 1H), 3.48–3.59 (m, 1H), 3.85 (br, 2H), 4.25–4.39 (m, 1H), 4.48–4.58 (m, 1H), 4.72–4.83 (m, 1H), 5.06 (br, 2H), 6.90 (d, *J* = 7.5 Hz, 1H), 7.10 (t, *J* = 7.7 Hz, 1H), 7.25–7.34 (m, 4H), 7.35–7.43 (m, 2H); LC-MS [M + H]^+^ 450.

#### *N*-Benzyl-*N*-(2-oxo-2-(4-(2-oxo-1,2-dihydroquinazolin-3(4*H*)-yl)piperidin-1-yl)ethyl)pivalamide (**4**)

**Scheme 4:**
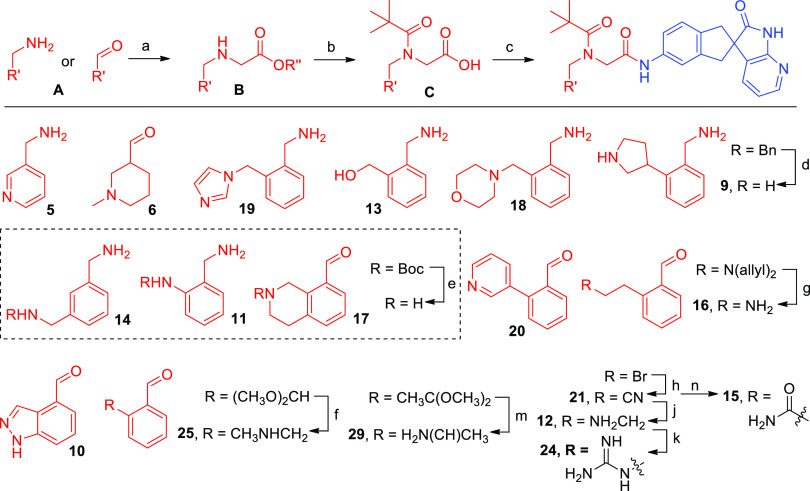
Synthesis of **5**, **6**, **9**–**14**, **16**–**21**, **24**, **25**, and **29** (a) Ethyl bromoacetate, SIPEA, DMF, rt or benzyl bromoacetate, Et_3_N, THF, rt (from amine) or glycine ethyl ester hydrochloride, NaBH_3_CN, MeOH, rt (fromaldehyde or ketone); (b) (i) PivCl, DIPEA, DCM, rt; (ii) 2.5 N NaOH, MeOH, rt; (c) **D**, HATU, NMM, DMF, rt or **D**, EDCl, HOAt, DIPEA, DMF, rt; (d) H_2_, Pd/C, NH_4_COOH, MeOH, reflux; (e) TFA, DCM, rt or TsOH, MeOH, rt; (f) (i) *p*TsOH, acetone, rt; (ii) MeNH_2_, HCl, DIPEA, Na_2_SO_4_, DCM, rt then NaBH(OAc)_3_, rt; (g) 20% Pd(PPh_3_)_4_, 1,3-dimethylbarbituric acid, DCM, 35 °C; (h) Zn(CN)_2_, Pd(PPh_3_)_4_, DMF, 130 °C, MW; (j) H_2_, Raney-Ni, 2M NH_3_ in MeOH, 55 °C; (k) 4-benzyl-3,5-dimethyl-1*H*-pyrazole-1-carboximidamide hydrochloride, 5 equiv, Et_3_N, MeCN/THF, MW, 90 °C; (m) (i) *p*TsOH, acetone, rt; (ii) NH_4_OAc, MeOH, reflux; then NaBH_3_CN, rt; (n) H_2_O_2_, H_2_O, NaOH, DMSO, rt.

**2A** (12.5 mg, 0.05 mmol), **2B** (15 mg, 0.06 mmol), and HATU (23 mg, 0.06 mmol) were dissolved in dry DMF (2 mL). *N*-Methylmorpholine (0.1 mL, 1.75 mmol) was added, and the mixture was stirred at rt for 5 min. The mixture was diluted with ethyl acetate, washed with brine, dried over magnesium sulfate, filtered, and the filtrate was evaporated. The crude was purified using a Biotage Isolera (12 g, C18 Ultra cartridge, 60–80% acetonitrile/water with pH 10 buffer) and freeze-dried to provide **2** as a white solid (18.3 mg, 79%). ^1^H NMR (CD_3_OD, 300 MHz) δ 1.38 (s, 9H), 1.66–1.76 (m, 2H), 1.77–1.89 (m, 1H), 3.06–3.17 (m, 1H), 3.86–3.98 (m, 1H), 4.22 (br, 2H), 4.38 (s, 2H), 4.43–4.53 (m, 1H), 4.59–4.72 (m, 1H), 5.06 (br, 2H), 6.79 (d, *J* = 7.9 Hz, 1H), 6.94 (t, *J* = 7.5 Hz, 1H), 7.11–7.17 (m, 1H), 7.26–7.34 (m, 4H), 7.36–7.43 (m, 2H); LC-MS [M + H]^+^ 463. (Scheme 4)

#### Ethyl 2-((Pyridin-2-ylmethyl)amino)acetate (**5B**)

(2-Methylamino)pyridine **5A** (6 g, 55.4 mmol) was dissolved in dry THF (45 mL), and ethyl bromoacetate (4.63 g, 27.7 mmol) was added dropwise at 0 °C. The mixture was stirred at rt for 2 h. The reaction mixture was poured into water and extracted three times with ethyl acetate. The organic layer was washed twice with ammonium chloride, dried over sodium sulfate, filtered, and evaporated. The residue was purified by flash chromatography (1:1 ethyl acetate/heptane to 1:10 methanol/ethyl acetate) to provide **5B** (1.43 g, quantitative yield). (2.55 g, 47%) as an orange oil. UPLC-MS (short basic) ^*t*^R 1.30 (195 [M + H]^+^).

#### Ethyl 2-(*N*-(Pyridin-2-ylmethyl)pivalamido)acetate

**5B** (1 g, 5.14 mmol) was dissolved in dichloromethane (30 mL) under an argon atmosphere before trimethylamine (1.55 g, 15.42 mmol) was added. Trimethylacetyl chloride (743 mg, 6.17 mmol) was added dropwise at 0 °C. The mixture was stirred at rt for 2 h after which the reaction was shown to be complete by TLC. The mixture was poured into water, and the aqueous layer was extracted three times with dichloromethane. The combined organic extracts were washed with brine, dried over magnesium sulfate, filtered, and the filtrate was evaporated to provide the crude product. The residue was purified by flash chromatography (1:1–1:0 ethyl acetate/heptane) to provide ethyl 2-(*N*-(pyridin-2-ylmethyl)pivalamido)acetate (1.45 g, quantitative yield). UPLC-MS (short basic) ^*t*^R 0.69 (279 [M + H]^+^).

#### 2-(*N*-(Pyridin-2-ylmethyl)pivalamido)acetic Acid (**5C**)

Ethyl 2-(*N*-(pyridin-2-ylmethyl)pivalamido)acetate (1.45 g, 5.21 mmol) was dissolved in THF (7 mL) and water (7 mL) and then lithium hydroxide monohydrate (655 mg, 15.62 mmol) was added, and the mixture was stirred at rt for 16 h. The aqueous pH was again adjusted to 6 and extracted with ethyl acetate (repeated 3 × 30 mL). The organics were washed with brine, dried over magnesium sulfate, filtered, and the filtrate was evaporated to provide **5C** as a yellow oil (871 mg, 67%). ^1^H NMR (DMSO-*d*_6_, 300 MHz) δ 1.15 (s, 9H), 3.32 (s, 2H), 4.75 (s, 2H), 7.49–7.61 (m, 1H), 7.81–7.92 (m, 1H), 8.51–8.60 (m, 2H). UPLC-MS (short basic) ^*t*^R 0.42 (251 [M + H]^+^).

#### *N*-(2-Oxo-2-((2′-oxo-1,1′,2′,3-tetrahydrospiro[indene-2,3′-pyrrolo[2,3-*b*]pyridin]-5-yl)amino)ethyl)-*N*-(pyridin-2-ylmethyl)pivalamide (**5**)

**5C** (50 mg, 0.20 mmol), **5D** (50 mg, 0.20 mmol), and HATU (75 mg, 0.20 mmol) were dissolved in dry DMF (2 mL). *N*,*N*-Diisopropylethylamine (76 mg, 0.59 mmol) was added, and the mixture was stirred at rt for 2 h. The mixture was diluted with ethyl acetate (50 mL) and washed with brine (3 × 30 mL), dried over magnesium sulfate, filtered, and the filtrate was evaporated. The crude was purified on reverse phase chromatography to provide **5** as a white solid (10.7 mg, 11%). ^1^H NMR (CD_3_OD, 300 MHz) δ 1.30 (s, 9H), 3.06 (dd, *J* = 15.8, 5.0 Hz, 2H), 3.50 (dd, *J* = 15.9, 7.9 Hz, 2H), 4.01 (s, 2H), 4.87 (s, 2H), 6.84–6.90 (m, 1H), 7.12 (d, *J* = 7.3 Hz, 1H), 7.23 (d, *J* = 8.0 Hz, 1H), 7.33–7.38 (m, 3H), 7.57 (s, 1H), 7.75–7.83 (m, 1H), 8.03 (dd, *J* = 5.3, 1.5 Hz, 1H), 8.44–8.49 (m, 1H). UPLC-MS (short basic) ^*t*^R 1.82 (484 [M + H]^+^).

#### Benzyl 2-(((1-Methylpiperidin-3-yl)methyl)amino)acetate (**6B**)

**6A** (0.5 g, 3.90 mmol) was dissolved in THF (10 mL), and triethylamine (1.1 mL, 7.80 mmol) and benzylbromoacetate (0.61 mL, 3.90 mmol) were added. The mixture was stirred at rt for 60 h. The mixture was poured into saturated sodium bicarbonate, and the aqueous layer was extracted three times with dichloromethane. The combined organic layers were dried over magnesium sulfate, filtered, and evaporated. The residue was purified via flash silica chromatography (19:1 dichloromethane/methanol) to provide **6B** (318 mg, 30%). ^1^H NMR (CDCl_3_, 300 MHz) δ 0.80–0.97 (m, 1H), 1.40–1.91 (m, 6H), 2.24 (s, 3H), 2.40–2.55 (m, 2H), 2.75 (d, *J* = 11.1 Hz, 1H), 2.87 (d, *J* = 10.7 Hz, 2H), 3.43 (s, 2H), 5.16 (s, 2H), 7.29–7.40 (m, 5H). UPLC-MS (short basic) ^*t*^R 1.81 (277 [M + H]^+^), 79% pure.

#### Benzyl 2-(*N*-((1-Methylpiperidin-3-yl)methyl)pivalamido)acetate

**6B** (318 mg, 1.152 mmol) was dissolved in dichloromethane (8 mL) under an argon atmosphere and triethylamine (0.19 mL, 1.382 mmol) was added, and the mixture was stirred at 5 °C (ice/water). Trimethylacetyl chloride (0.14 mL, 1.152 mmol) was added dropwise, and the mixture was stirred at rt for overnight. The reaction mixture was diluted in dichloromethane, washed with brine, saturated sodium bicarbonate, dried over magnesium sulfate, filtered, and the filtrate was evaporated to provide the intermediate of **6C** (344 mg, 83%). ^1^H NMR (DMSO-*d*_6_, 400 MHz) δ 0.91 (d, *J* = 8.0 Hz, 1H), 1.13 (s, 9H), 1.32–1.70 (m, 4H), 2.07 (s, 3H), 2.78–2.90 (m, 2H), 3.05–3.15 (m, 2H), 3.15–3.32 (m, 2H), 4.12 (s, 2H), 5.10 (s, 2H), 7.25–7.37 (m, 5H). UPLC-MS (short basic) ^*t*^R 2.06 (361 [M + H]^+^), 88% pure.

#### 2-(*N*-((1-Methylpiperidin-3-yl)methyl)pivalamido)acetic Acid (**6C**)

An intermediate of **6C** (344 mg, 0.955 mmol) was dissolved in ethanol (10 mL), and palladium-on-carbon (10% wet, 34 mg) was added, the vessel was sealed, and an atmosphere of hydrogen was introduced at a 400 psi pressure. The mixture was stirred at rt overnight. The reaction was filtered through celite, washed with methanol, and the filtrate was evaporated to provide **6C** as a clear glass (240 mg, 93%). ^1^H NMR (DMSO-*d*_6_, 300 MHz) δ 0.88–1.02 (m,1H), 1.17 (s, 9H), 1.35–1.65 (m, 3H), 1.79–2.10 (m, 3H), 2.18 (s, 3H), 2.60 (d, *J* = 9.0 Hz, 2H), 3.22 (d, *J* = 7.0 Hz, 2H), 3.99 (s, 2H). UPLC-MS (short basic) ^*t*^R 0.42 (271 [M + H]^+^).

#### *N*-((1-Methylpiperidin-3-yl)methyl)-*N*-(2-oxo-2-((2′-oxo-1,1′,2′,3-tetrahydrospiro[indene-2,3′-pyrrolo[2,3-*b*]pyridin]-5-yl)amino)ethyl)pivalamide (**6**)

**6C** (53 mg, 0.119 mmol), EDCI·HCl (46 mg, 0.239 mmol), and HOAt (33 mg, 0.239 mmol) were dissolved in dry DMF (1 mL). *N*,*N*-Diisopropylethylamine (110 μL, 0.597 mmol) and **6D** (50 mg, 0.119 mmol) were added, and the mixture was stirred at rt overnight. The reaction mixture was poured into saturated sodium bicarbonate and extracted three times with ethyl acetate and brine, dried over magnesium sulfate, filtered, and the filtrate was evaporated. The crude was directly purified using a Biotage Isolera (18 g, C18 Ultra cartridge, 10–70% acetonitrile/water with pH 10 buffer) to provide a crude compound. This was further purified via SP4 (12 g, C18 cartridge, 5–75% acetonitrile in water with 0.1% ammonium hydroxide) to provide **6** (3.1 mg, 5%). ^1^H NMR (DMSO-*d*_6_, 300 MHz) δ 0.77–0.96 (m, 1H), 1.16 (s, 9H), 1.48–1.62 (m, 4H), 1.75–1.92 (m, 2H), 2.08 (s, 3H), 2.47–2.59 (m, 2H), 2.97–3.00 (m, 2H), 3.23–3.31 (m, 2H), 4.19 (s, 2H), 6.84 (dd, *J* = 7.3, 5.3 Hz, 1H), 7.12–7.21 (m, 2H), 7.33 (d, *J* = 7.9 Hz, 1H), 7.54 (s, 1H), 8.03 (dd, *J* = 5.3, 1.6 Hz, 1H), 9.97 (s, 1H), 11.08 (s, br, 1H). UPLC-MS (short basic) ^*t*^R 1.78 (504 [M + H]^+^).

#### *tert*-Butyl-2-((2-((1*H*-imidazol-1-yl)methyl)benzyl)amino)acetate (**19B**)

(2-((1*H*-Imidazol-1-yl)methyl)phenyl)methanamine dihydrochloride **19A** (200 mg, 0.76 mmol) and *N*,*N*-diisopropylethylamine (596 mg, 4.61 mmol) were dissolved in dry DMF (5 mL). A solution of *tert*-butyl bromoacetate (135 mg, 0.69 mmol) in DMF (1 mL) was added slowly. The mixture was stirred at rt for 18 h. The reaction mixture was combined with another batch of material (0.192 mmol), quenched with saturated aqueous ammonium chloride solution, and extracted three times with ethyl acetate. The organic extracts were dried over sodium sulfate, filtered, and evaporated to provide **19B** (100 mg, 22%) as a pale yellow solid that was used directly in the next step. UPLC-MS (short basic) rt 0.58 (302 [M + H]^+^).

#### *tert*-Butyl-2-(*N*-(2-((1*H*-imidazol-1-yl)methyl)benzyl)pivalamido)acetate

**19B** (50 mg, 0.166 mmol) was dissolved in dichloromethane (3 mL) under an argon atmosphere, and *N*,*N*-diisopropylethylamine (43 mg, 0.332 mmol) was added. Trimethylacetyl chloride (30 mg, 0.249 mmol) was added, and the mixture was stirred at rt overnight. Further trimethylacetyl chloride (20 mg, 0.166 mmol) was added. UPLC indicated that the reaction was incomplete. The mixture was quenched with saturated aqueous ammonium chloride solution and extracted three times with dichloromethane. The organic extracts were dried over sodium sulfate, filtered, and the filtrate was evaporated. The residue was dissolved in pyridine (1 mL), and trimethylacetyl chloride (96 mg, 0.797 mmol) was added. The mixture was stirred at rt for 2 h and evaporated. The residue was dissolved in water and extracted three times with ethyl acetate. The organic extracts were dried over sodium sulfate, filtered, and the filtrate was evaporated. The crude residue was purified via reverse phase chromatography (30 g, C18 cartridge acetonitrile/pH 10 buffer with ammonium bicarbonate) to provide the intermediate of **19C** (80 mg, 83%) as a white solid that was used directly in the next step. UPLC-MS (long basic) rt 2.02 (386 [M + H]^+^).

#### 2-(*N*-(2-((1*H*-Imidazol-1-yl)methyl)benzyl)pivalamido)acetic Acid (**19C**)

An intermediate of **19C** (80 mg, 0.207 mmol) was dissolved in methanol (3 mL) and 2 M sodium hydroxide (0.311 mL, 0.622 mmol) was added, and the mixture was stirred at rt for 2 days. The mixture was acidified to pH 5 with 2 M aqueous HCl solution and extracted twice with ethyl acetate. The organic extracts were dried over sodium sulfate, evaporated, filtered, and the filtrate was evaporated to provide **19C** (40 mg, 59%) as a white solid. ^1^H NMR (CD_3_OD, 300 MHz) δ 1.29 (s, 9H), 3.98 (s, 2H), 4.72 (s, 2H), 5.25 (s, 2H), 6.92–7.03 (m, 2H), 7.17–7.27 (m, 2H), 7.22–7.43 (m, 3H), 7.75 (s, br, 1H). UPLC-MS (short basic) ^*t*^R 0.46 (330 [M + H]^+^), 100% pure.

#### *N*-(2-((1*H*-Imidazol-1-yl)methyl)benzyl)-*N*-(2-oxo-2-((2′-oxo-1,1′,2′,3-tetrahydrospiro[indene-2,3′-pyrrolo[2,3-*b*]pyridin]-5-yl)amino)ethyl)pivalamide (**19**)

**19C** (40 mg, 0.121 mmol), EDCI·HCl (25 mg, 0.182 mmol), and HOAt (35 mg, 0.182 mmol) were dissolved in dry DMF (2 mL). *N*,*N*-Diisopropylethylamine (83 mg, 0.73 mmol) and **19D** (37 mg, 0.147 mmol) were added, and the mixture was stirred at rt for 18 h. The mixture was poured into saturated ammonium chloride, and the aqueous layer was extracted three times with ethyl acetate. The organic extract was washed three times with sodium bicarbonate, dried over sodium sulfate, filtered, and the filtrate was evaporated. The residue was purified via reverse phase chromatography (SP4 30 g, C18 cartridge acetonitrile/pH 10 buffer with ammonium bicarbonate) to provide **19** (16 mg, 24%) as a colorless glass. ^1^H NMR (CD_3_OD, 300 MHz) δ 1.20 (s, br, 9H), 3.03 (d, *J* = 15.7 Hz, 2H), 3.48 (d, *J* = 15.9 Hz, 2H), 4.01 (s, br, 2H), 4.76 (s, 2H), 5.29 (s, 2H), 6.81–7.46 (m, 10H), 7.52 (s, 1H), 7.68 (s, 1H), 8.02 (d, *J* = 5.5 Hz, 1H). UPLC-MS (long basic) ^*t*^R 1.87 (563 [M + H]^+^), 100% pure.

#### Ethyl 2-((2-(Hydroxymethyl)benzyl)amino)acetate (**13B**)

**13A** (200 mg, 1.15 mmol) was dissolved in THF (2 mL) and dry DMF (4 mL), and triethylamine (744 mg, 5.76 mmol) and ethyl bromoacetate (173 mg, 1.04 mmol) were added. The mixture was stirred at rt for 3 h. The mixture was poured into saturated sodium bicarbonate, and the aqueous layer was extracted three times with ethyl acetate. The combined organic layers were dried over magnesium sulfate, filtered, and evaporated. The residue was purified via reverse phase chromatography (SP4 30 g, C18 cartridge acetonitrile/pH 10 buffer with ammonium bicarbonate) to provide **13B** (200 mg, 52%). ^1^H NMR (CDCl_3_, 300 MHz) δ 1.27 (t, *J* = 7.1 Hz, 3H), 3.40 (s, 2H), 3.80 (s, 2H), 4.19 (q, *J* = 7.2 Hz, 2H), 4.69 (s, 2H), 7.22–7.37 (m, 4H). UPLC-MS (short basic) ^*t*^R 0.62 (224 [M + H]^+^).

#### Ethyl 2-(*N*-(2-(Hydroxymethyl)benzyl)pivalamido)acetate

**13B** (210 mg, 0.94 mmol) was dissolved in dichloromethane (10 mL) and dry DMF (2 mL) under an argon atmosphere, *N*,*N*-diisopropylethylamine (364 mg, 2.82 mmol) was added, and the mixture was stirred at 5 °C (ice/water). Trimethylacetyl chloride (113 mg, 0.94 mmol) was added dropwise, and the mixture was stirred at rt overnight. The reaction mixture was diluted in dichloromethane, washed with brine and saturated ammonium chloride, dried over magnesium sulfate, filtered, and the filtrate was evaporated. The residue was purified via reverse phase chromatography (SP4 30 g, C18 cartridge acetonitrile/pH 10 buffer with ammonium bicarbonate) to provide intermediate **13C** (110 mg, 38%). UPLC-MS (short basic) ^*t*^R 0.71 (308 [M + H]^+^).

#### 2-(*N*-(2-(Hydroxymethyl)benzyl)pivalamido)acetic Acid (**13C**)

Intermediate **13C** (110 mg, 0.36 mmol) was dissolved in THF (3 mL). Methanol (3 mL) and lithium hydroxide monohydrate (45 mg, 1.07 mmol) were added, and the mixture was stirred at rt for 3 h. The pH was adjusted carefully to 4 by the addition of 2 M HCl and extracted with ethyl acetate. The volatiles were removed, and the residue was purified via reverse phase chromatography (SP4 30 g, C18 cartridge acetonitrile/pH 10 buffer with ammonium bicarbonate) to provide **13C** (60 mg, 60%). ^1^H NMR (CD_3_OD, 300 MHz) δ 1.24 (s, 9H), 3.86 (s, br, 2H), 4.61 (s, 2H), 4.90 (s, 2H), 7.00–7.30 (m, 4H). UPLC-MS (short basic) ^*t*^R 0.44 (280 [M + H]^+^).

#### *N-*(2-(Hydroxymethyl)benzyl)-*N*-(2-oxo-2-((2′-oxo-1,1′,2′,3-tetrahydrospiro[indene-2,3′-pyrrolo[2,3-*b*]pyridin]-5-yl)amino)ethyl)pivalamide (**13**)

**13C** (60 mg, 0.21 mmol), EDCI·HCl (62 mg, 0.32 mmol), and HOAt (44 mg, 0.32 mmol) were dissolved in dry DMF (4 mL). *N*,*N*-Diisopropylethylamine (83 mg, 0.64 mmol) and **13D** (54 mg, 0.21 mmol) were added, and the mixture was stirred at rt overnight. The mixture was poured into saturated ammonium chloride, and the aqueous layer was extracted twice with ethyl acetate. The organic extract was dried over sodium sulfate, filtered, and the filtrate was evaporated. The residue was purified via reverse phase chromatography (SP4 30 g, C18 cartridge acetonitrile/pH 10 buffer with ammonium bicarbonate) to provide **13** (25 mg, 23%). ^1^H NMR (CD_3_OD, 400 MHz) δ 1.32 (s, 9H), 2.95 (dd, *J* = 15.6, 10.6 Hz, 2H), 3.51 (dd, *J* = 15.8, 7.5 Hz, 2H), 4.09 (s, 2H), 4.63 (s, 2H), 5.00 (s, br, 2H), 6.74–6.83 (m, 1H), 7.07 (dd, *J* = 16.4, 7.6 Hz, 1H), 7.15–7.37 (m, 6H), 7.46 (s, 1H), 8.07 (s, 1H), 8.87 (s, 1H). UPLC-MS (long basic) ^*t*^R 1.83 (513 [M + H]^+^).

#### Ethyl 2-((2-(Morpholinomethyl)benzyl)amino)acetate

**18A** (100 mg, 0.48 mmol) was dissolved in dry DMF (2.5 mL) and *N*,*N*-diisopropylethylamine (0.47 mL, 2.58 mmol). Ethyl bromoacetate (72 mg, 0.43 mmol) was added dropwise at 0 °C. The mixture was stirred at rt for 5 h. The reaction mixture was poured into water and extracted three times with ethyl acetate. The organic layer was washed twice with ammonium chloride, dried over sodium sulfate, filtered, and evaporated to provide **18B** (70 mg) that was used directly in the next step. UPLC-MS (short basic) ^*t*^R 0.73 (293 [M + H]^+^).

#### Ethyl 2-(*N*-(2-(Morpholinomethyl)benzyl)pivalamido)acetate

**18B** (70 mg, 0.24 mmol) was dissolved in dichloromethane (3 mL) under an argon atmosphere and then *N*,*N*-diisopropylethylamine (62 mg, 0.48 mmol) was added, and the mixture was stirred at 5 °C (ice/water). Trimethylacetyl chloride (110 μL, 0.89 mmol) was added dropwise, and then the mixture was stirred at rt over the weekend. The reaction mixture was diluted in dichloromethane, washed with brine and saturated ammonium chloride, dried over magnesium sulfate, filtered, and the filtrate was evaporated. The residue was purified via reverse phase chromatography (SP4 30 g, C18 cartridge acetonitrile/pH 10 buffer with ammonium bicarbonate) to provide the intermediate of **18C** (40 mg, 44%). UPLC-MS (short basic) ^*t*^R 0.87 (377 [M + H]^+^).

#### 2-(*N*-(2-(Morpholinomethyl)benzyl)pivalamido)acetic Acid

An intermediate of **18C** (40 mg, 0.17 mmol) was dissolved in methanol (2 mL) and 2.0 M sodium hydroxide (159 μL, 0.318 mmol) was added, and the mixture was stirred at rt overnight. The volatiles were removed, and then the residue was dissolved in water. The pH was adjusted carefully to 4 by the addition of 2 M HCl, and the crude material was extracted with ethyl acetate. The aqueous pH was again adjusted to 4, and the product was extracted with ethyl acetate. The organics were washed with brine, dried over magnesium sulfate, filtered, and the filtrate was evaporated to provide **18C** (59 mg, quant.) that was used directly in the next step. UPLC-MS (short basic) ^*t*^R 0.50 (349 [M + H]^+^).

#### *N*-(2-(Morpholinomethyl)benzyl)-*N*-(2-oxo-2-((2′-oxo-1,1′,2′,3-tetrahydrospiro[indene-2,3′-pyrrolo[2,3-*b*]pyridin]-5-yl)amino)ethyl)pivalamide (**18**)

**18C** (40 mg, 0.11 mmol), EDCI·HCl (33 mg, 0.17 mmol), and HOAt (22 mg, 0.17 mmol) were dissolved in dry DMF (3 mL). *N*,*N*-Diisopropylethylamine (89 mg, 0.68 mmol) and **18D** (35 mg, 0.14 mmol) were added, and the mixture was stirred at rt for 5 h. The mixture was poured into saturated ammonium chloride, and the aqueous layer was extracted twice with ethyl acetate. The organic extract was dried over sodium sulfate, filtered, and the filtrate was evaporated. The residue was purified via reverse phase chromatography (SP4 30 g, C18 cartridge acetonitrile/pH 10 buffer with ammonium bicarbonate) to provide **18** (44 mg, 66%). ^1^H NMR (CD_3_OD, 300 MHz) δ 1.32 (s, 9H), 2.38 (s, br, 4H), 3.05 (dd, *J* = 15.8, 5.6 Hz, 2H), 3.48 (dd, *J* = 15.6, 10.5 Hz, 2H), 3.52–3.61 (m, 4H), 4.05 (s, 2H), 5.21 (s, 2H), 6.86 (dd, *J* = 7.4, 5.4 Hz, 1H), 7.10 (dd, *J* = 7.4, 1.6 Hz, 1H), 7.17–7.25 (m, 4H), 7.32 (s, br, 1H), 7.37 (d, *J* = 8.4 Hz, 1H), 7.55 (s, 1H), 8.03 (dd, *J* = 5.4, 1.6 Hz, 1H). UPLC-MS (long basic) ^*t*^R 2.27 (582 [M + H]^+^).

#### (2-(1-Benzylpyrrolidin-3-yl)phenyl)methanamine (**9A**)

NaBH_4_ (0.75 g, 20 mmol) was carefully added to a solution of the corresponding nitrile **9E** (1 g, 3.82 mmol) and CoCl_2_ (25 mg, 0.19 mmol) in methanol (40 mL) at room temperature. The mixture was stirred at rt for 4 h. The reaction mixture was slowly quenched with saturated ammonium chloride (4 mL), diluted with ethyl acetate, and filtered through celite. The aqueous layer was extracted three times with ethyl acetate. The organic layer was washed with ammonium chloride, dried over sodium sulfate, filtered, and evaporated. The crude was purified using a Biotage Isolera (18 g, C18 Ultra cartridge, 30–60% acetonitrile/water with pH 10 buffer) to provide **9A** (356 mg, 35%). UPLC-MS (short basic) ^*t*^R 0.48 (267 [M + H]^+^).

#### Ethyl 2-((2-(1-Benzylpyrrolidin-3-yl)benzyl)amino)acetate (**9B**)

**9A** (40 mg, 0.15 mmol) was dissolved in dry DMF (2 mL), and triethylamine (74 mg, 0.57 mmol) and ethyl bromoacetate (27 mg, 0.16 mmol) were added. The mixture was stirred at rt overnight. The mixture was poured into saturated sodium bicarbonate, and the aqueous layer was extracted three times with ethyl acetate. The organic layer was washed twice with ammonium chloride, dried over sodium sulfate, filtered, and evaporated to provide **9B** (63 mg). UPLC-MS (short basic) ^*t*^R 0.62 (353 [M + H]^+^).

#### Ethyl 2-(*N*-(2-(1-Benzylpyrrolidin-3-yl)benzyl)pivalamido)acetate

**9B** (63 mg, 0.178 mmol) was dissolved in dichloromethane (3 mL) under an argon atmosphere and *N*,*N*-diisopropylethylamine (0.40 μL, 0.45 mmol) was added, and the mixture was stirred at 5 °C (ice/water). Trimethylacetyl chloride (25 μL, 0.20 mmol) was added dropwise, and the mixture was stirred at rt for 4 h. The reaction mixture was diluted in dichloromethane, washed with brine and saturated sodium bicarbonate, dried over magnesium sulfate, filtered, and the filtrate was evaporated. The residue was purified via flash silica chromatography (5:1–1:1 heptane/EtOAc) to provide methyl 2-(*N*-(2-(1-benzylpyrrolidin-3-yl)benzyl)pivalamido)acetate (52 mg, 67%). UPLC-MS (short basic) ^*t*^R 1.05 (437 [M + H]^+^).

#### 2-(*N*-(2-(1-Benzylpyrrolidin-3-yl)benzyl)pivalamido)acetic Acid (**9C**)

Methyl 2-(*N*-(2-(1-benzylpyrrolidin-3-yl)benzyl)pivalamido)acetate (52 mg, 0.13 mmol) was dissolved in a mixture of methanol (2 mL), tetrahydrofuran (2 mL), and water (1 mL), and lithium hydroxide monohydrate (16 mg, 0.65 mmol) was added. The reaction mixture was stirred overnight before the pH was adjusted carefully to 4 by the addition of 2 M HCl and volatiles were removed. The crude product was directly purified via flash silica chromatography (5–30% methanol/dichloromethane) to provide the desired **9C** as a colorless solid (50 mg, 95%). UPLC-MS (short basic) ^*t*^R 0.51 (409 [M + H]^+^).

#### *N*-(2-(1-Benzylpyrrolidin-3-yl)benzyl)-*N*-(2-oxo-2-((2′-oxo-1,1′,2′,3-tetrahydrospiro[indene-2,3′-pyrrolo[2,3-*b*]pyridin]-5-yl)amino)ethyl)pivalamide (**9F**)

**9C** (50 mg, 0.12 mmol), **9D** (30 mg, 0.12 mmol), and HATU (54 mg, 0.14 mmol) were dissolved in dry DMF (2.5 mL). *N*-Methylmorpholine (0.25 mL) was added, and the mixture was stirred at room temperature for 10 min. The mixture was diluted with ethyl acetate and washed with brine, dried over magnesium sulfate, filtered, and the filtrate was evaporated. The residue was purified via flash silica chromatography (70–100% ethyl acetate/petrol ether) to provide **9F** (64 mg, 83%) as a colorless glass. ^1^H NMR (CDCl_3_, 400 MHz) δ 1.30 (s, 9H), 1.76–1.85 (m, 1H), 2.30–2.39 (m, 1H), 2.62–2.79 (m, 3H), 2.91 (t, *J* = 8.6 Hz, 1H), 3.03 (dd, *J* = 15.8, 6.2 Hz, 2H), 3.45–3.51 (m, 1H), 3.56–3.73 (m, 4H), 4.03 (s, 2H), 4.93 (s, 2H), 6.80 (dd, *J* = 7.1, 5.5 Hz, 1H), 7.02–7.07 (m, 2H), 7.15–7.37 (m, 8H), 7.48 (d, *J* = 7.6 Hz, 1H), 7.52 (s, 1H), 8.11 (d, *J* = 5.3, 1.5 Hz, 1H), 8.43 (s, 1H). UPLC-MS (short basic) ^*t*^R 0.91 (642 [M + H]^+^), 99% pure.

#### *N*-(2-Oxo-2-((2′-oxo-1,1′,2′,3-tetrahydrospiro[indene-2,3′-pyrrolo[2,3-*b*]pyridin]-5-yl)amino)ethyl)-*N*-(2-(pyrrolidin-3-yl)benzyl)pivalamide (**9**)

**9F** (59 mg, 0.09 mmol) and Pd/C (10 mg) were dissolved in methanol (5 mL) followed by the addition of NH_4_COOH (57 mg, 0.9 mmol), and the mixture was refluxed for 4 h. The reaction mixture was diluted with ethyl acetate and filtered. The solvent was evaporated under reduced pressure, and the residue was purified (500 mg SCX-2 MeOH to ammonia in MeOH) to provide **9** (28 mg, 55%) as a colorless solid. ^1^H NMR (CD_3_OD, 300 MHz) δ 1.33 (s, 9H), 2.02–2.15 (m, 1H), 2.32–2.43 (m, 1H), 3.00–3.12 (m, 3H), 3.14–3.25 (m, 1H), 3.30–3.38 (m, 1H), 3.40–3.59 (m, 3H), 3.61–3.76 (m, 2H), 4.17 (s, 2H), 4.90 (br s, 2H), 6.87 (d, *J* = 7.3, 5.4 Hz, 1H), 7.12 (dd, *J* = 7.4, 1.3 Hz, 1H), 7.17–7.24 (m, 2H), 7.28–7.38 (m, 3H), 7.40–7.45 (m, 1H), 7.51–7.55 (m, 1H), 8.02 (dd, *J* = 5.3, 1.4 Hz, 1H). UPLC-MS (short basic) ^*t*^R 0.66 (552 [M + H]^+^), 97% pure.

#### Ethyl 2-((3-(((*tert*-Butoxycarbonyl)amino)methyl)benzyl)amino)acetate (**14B**)

**14A** (150 mg, 0.635 mmol) was dissolved in THF (4 mL), and triethylamine (0.13 mL, 0.825 mmol) and ethyl bromoacetate (63 μL, 0.571 mmol) were added. The mixture was stirred at rt for 2 h. The mixture was poured into saturated sodium bicarbonate, and the aqueous layer was extracted three times with ethyl acetate. The combined organic layers were dried over magnesium sulfate, filtered, and evaporated. The residue was purified via reverse phase chromatography (SP4 30 g, C18 cartridge acetonitrile/pH 10 buffer with ammonium bicarbonate) to provide **14B** (90 mg, 44%). ^1^H NMR (CD_3_OD, 300 MHz) δ 1.24 (t, *J* = 7.1 Hz, 3H), 1.44 (s, 9H), 3.33 (s, 2H), 3.74 (s, 2H), 4.16 (q, *J* = 6.9 Hz, 2H), 4.21 (s, 2H), 7.15–7.31 (m, 4H). UPLC-MS (short basic) ^*t*^R 0.73 (323 [M + H]^+^).

#### Ethyl 2-(*N*-(3-(((*tert*-Butoxycarbonyl)amino)methyl)benzyl)pivalamido)acetate

**14B** (90 mg, 0.279 mmol) was dissolved in dichloromethane (2 mL) under an argon atmosphere and *N*,*N*-diisopropylethylamine (0.72 μL, 0.42 mmol) was added, and the mixture was stirred at 5 °C (ice/water). Trimethylacetyl chloride (34 μL, 0.28 mmol) was added dropwise, and the mixture was stirred at rt over the weekend. The reaction mixture was diluted in dichloromethane, washed with brine and saturated sodium bicarbonate, dried over magnesium sulfate, filtered, and the filtrate was evaporated. The residue was purified via flash silica chromatography (1:1 heptane/EtOAc) to provide ethyl 2-(*N*-(3-(((*tert*-butoxycarbonyl)amino)methyl)benzyl)pivalamido)acetate (95 mg, 84%). LC-MS ^*t*^R 2.19 (407 [M + H]^+^).

#### 2-(*N*-(3-(((*tert*-Butoxycarbonyl)amino)methyl)benzyl)pivalamido)acetic Acid (**14C**)

Ethyl 2-(*N*-(3-(((*tert*-butoxycarbonyl)amino)methyl)benzyl)pivalamido)acetate (95 mg, 0.234 mmol) was dissolved in THF (1 mL) and methanol (1 mL), and lithium hydroxide monohydrate (15 mg, 0.351 mmol) was added and the mixture was stirred at rt overnight. The pH was adjusted carefully to 4 by the addition of 2 M HCl, and the product was extracted with dichloromethane. The volatiles were removed to provide **14C** (76 mg, 80%). ^1^H NMR (CDCl_3_, 300 MHz) δ 1.32 (s, 9H), 1.44 (s, 9H), 3.91 (s, br, 2H), 4.20–4.33 (m, 2H), 4.80 (s, 2H), 4.97–5.06 (m, 1H), 7.06–7.10 (m, 2H), 7.15–7.23 (d, 1H), 7.27–7.35 (m, 1H). LC-MS ^*t*^R 1.54 (379 [M + H]^+^).

#### *tert*-Butyl 3-((*N*-(2-Oxo-2-((2′-oxo-1,1′,2′,3-tetrahydrospiro[indene-2,3′-pyrrolo[2,3-*b*]pyridin]-5-yl)amino)ethyl)pivalamido)methyl)benzylcarbamate (**14E**)

**14C** (71 mg, 0.188 mmol), EDCI·HCl (43 mg, 0.226 mmol), and HOAt (30 mg, 0.226 mmol) were dissolved in dry DMF (2 mL). *N*,*N*-Diisopropylethylamine (0.11 mL, 0.678 mmol) and **14D** (47 mg, 0.188 mmol) were added, and the mixture was stirred at rt overnight. The mixture was diluted with ethyl acetate and washed with saturated sodium bicarbonate. The aqueous layer was extracted twice with ethyl acetate. The combined organics were washed three times with water and then with brine, dried over magnesium sulfate, filtered, and the filtrate was evaporated. The residue was purified via flash silica chromatography (5% methanol/dichloromethane) to provide **14E** (60 mg, 52%). ^1^H NMR (CDCl_3_, 400 MHz) δ 1.37 (s, 9H), 1.45 (s, 9H), 3.03 (dd, *J* = 15.7, 5.7 Hz, 2H), 3.60 (dd, *J* = 15.8, 6.0 Hz, 2H), 3.99 (s, 2H), 4.28 (d, *J* = 5.5 Hz, 2H), 4.81–4.91 (m, 2H), 6.81 (dd, *J* = 7.3, 5.3 Hz, 1H), 7.06 (dd, *J* = 7.3, 1.3 Hz, 1H), 7.10–7.23 (m, 5H), 7.32 (t, *J* = 7.8 Hz, 1H), 7.52 (s, 1H), 8.11 (d, *J* = 5.3, 1.5 Hz, 1H), 8.40–8.46 (m, 2H). UPLC-MS (long basic) ^*t*^R 2.36 (612 [M + H]^+^), 96% pure.

#### *N*-(3-(Aminomethyl)benzyl)-*N*-(2-oxo-2-((2′-oxo-1,1′,2′,3-tetrahydrospiro[indene-2,3′-pyrrolo[2,3-*b*]pyridin]-5-yl)amino)ethyl)pivalamide (**14**)

**14E** (30 mg, 0.049 mmol) was dissolved in methanol (2 mL), and *p*-toluene sulfonic acid monohydrate (19 mg, 0.10 mmol) was added. The mixture was stirred at 50 °C for 3.5 h and poured into saturated sodium bicarbonate. The aqueous layer was extracted with ethyl acetate. The organic extract was washed with brine, dried over magnesium sulfate, filtered, and the filtrate was evaporated. The residue was purified via SPE (2 g SiO_2_, EtOAc and then 10% MeOH in DCM) to provide **14** (5 mg, 20%). ^1^H NMR (CD_3_OD, 300 MHz) δ 1.34 (s, 9H), 3.05 (dd, *J* = 15.9, 3.7 Hz, 2H), 3.49 (dd, *J* = 15.6, 7.5 Hz, 2H), 3.88 (s, 2H), 4.06 (s, br, 2H), 4.88 (s, br, 2H), 6.87 (dd, *J* = 7.1, 1.5 Hz, 1H), 7.12 (d, *J* = 7.3 Hz, 1H), 7.21 (d, *J* = 8.0 Hz, 2H), 7.26–7.39 (m, 4H), 7.52 (s, 1H), 8.03 (d, *J* = 5.5 Hz, 1H). LC-MS ^*t*^R 4.98 (512 [M + H]^+^), 95% pure.

#### Methyl 2-(((1*H*-Indazol-4-yl)methyl)amino)acetate (**10B**)

**10A** (124 mg, 0.85 mmol) was dissolved in methanol (2.5 mL), and then methyl glycinate hydrochloride (320 mg, 2.52 mmol) and sodium cyanoborohydride (80 mg, 1.27 mmol) were added and the mixture was stirred at rt over the weekend. The reaction mixture was poured into water, and the pH was adjusted to 4 with 2 M HCl and washed twice with dichloromethane. The aqueous layer was basified with sodium carbonate and extracted twice with dichloromethane. This organic extract was dried over magnesium sulfate, filtered, and evaporated. The residue was purified by Isolera (acetonitrile/NH_4_COOH buffer pH = 10) to provide **10B** (35 mg, 19%). UPLC-MS (short basic) ^*t*^R 1.46 (220 [M + H]^+^).

#### Methyl 2-(*N*-((1*H*-Indazol-4-yl)methyl)pivalamido)acetate

**10B** (35 mg, 0.15 mmol) was dissolved in dichloromethane (2 mL) and tetrahydrofuran (2 mL) under an argon atmosphere, and *N*,*N*-diisopropylethylamine (0.08 mL, 0.5 mmol) was added and the mixture was stirred at 5 °C (ice/water). Trimethylacetyl chloride (20 μL, 0.16 mmol) was added dropwise, and the mixture was stirred at rt over the weekend. The reaction mixture was diluted in dichloromethane, washed with brine and saturated ammonium chloride, dried over magnesium sulfate, filtered, and the filtrate was evaporated to provide crude methyl 2-(*N*-((1*H*-indazol-4-yl)methyl)pivalamido)acetate (60 mg, 124%) that was used directly in the next step. UPLC-MS (short basic) ^*t*^R 0.66 (302 [M + H]^+^).

#### 2-(*N*-((1*H*-Indazol-4-yl)methyl)pivalamido)acetic Acid

Methyl 2-(*N*-((1*H*-indazol-4-yl)methyl)pivalamido)acetate (60 mg, 0.20 mmol) was dissolved in methanol (2.2 mL), and 2.5 M sodium hydroxide (0.12 mL, 0.30 mmol) was added and the mixture was stirred at rt over the weekend. The volatiles were removed, and the residue was dissolved in water. The pH was adjusted carefully to 4 by the addition of 2 M HCl and extracted with ethyl acetate. The aqueous pH was again adjusted to 4 and extracted with ethyl acetate. The organics were washed with brine, dried over magnesium sulfate, filtered, and the filtrate was evaporated to provide **10C** (49 mg, 86%) that was used directly in the next step. UPLC-MS (short basic) ^*t*^R 0.73 (323 [M + H]^+^). UPLC-MS (short basic) ^*t*^R 0.49 (290 [M + H]^+^).

#### *N*-((1*H*-Indazol-4-yl)methyl)-*N*-(2-oxo-2-((2′-oxo-1,1′,2′,3-tetrahydrospiro[indene-2,3′-pyrrolo[2,3-*b*]pyridin]-5-yl)amino)ethyl)pivalamide (**10**)

**10C** (35 mg, 0.12 mmol), EDCI·HCl (32 mg, 0.16 mmol), and HOAt (27 mg, 0.20 mmol) were dissolved in dry DMF (1.1 mL). *N*,*N*-Diisopropylethylamine (62 μL, 0.63 mmol) and **10D** (29 mg, 0.12 mmol) were added, and the mixture was stirred at rt overnight. The mixture was poured into saturated ammonium chloride, and the aqueous layer was extracted twice with ethyl acetate. The organic extract was dried over sodium sulfate, filtered, and the filtrate was evaporated. The crude was directly purified using a Biotage Isolera (12 g, C18 Ultra cartridge, 20–40% acetonitrile/water with pH 10 buffer) to provide crude **10** (31 mg, 49%) as a colorless solid. ^1^H NMR (CD_3_OD, 300 MHz) δ 1.24 (s, 9H), 3.03 (dd, *J* = 16.0, 10.7 Hz, 2H), 3.30 (dd, *J* = 16.0, 9.2 Hz, 2H), 4.04 (s, 2H), 5.02 (s, 2H), 6.82 (dd, *J* = 7.3, 5.3 Hz, 1H), 6.90 (d, *J* = 6.9 Hz, 1H), 7.10–7.55 (m, 7H), 8.00–8.05 (m, 2H), 9.75 (s, 1H), 10.94 (s, 1H). UPLC-MS (long basic) ^*t*^R 1.81 (523 [M + H]^+^), 100% pure.

#### Methyl 2-((2-(Pyridin-3-yl)benzyl)amino)acetate (**20B**)

**20A** (629 mg, 3.34 mmol) was dissolved in methanol (9.8 mL), and then methyl glycinate hydrochloride (1.3 g, 10.3 mmol) and sodium cyanoborohydride (324 mg, 5.1 mmol) were added and the mixture was stirred at rt overnight. The reaction mixture was poured into water and the pH was adjusted to 4 with 2 M HCl and then washed twice with dichloromethane. The aqueous layer was basified with sodium carbonate and extracted twice with dichloromethane. This organic extract was dried over magnesium sulfate, filtered, and evaporated to provide **20B** (410 mg, 48%) as a colorless oil. UPLC-MS (short basic) ^*t*^R 0.62 (257 [M + H]^+^), 96% pure.

#### Methyl 2-(*N*-(2-(Pyridin-3-yl)benzyl)pivalamido)acetate

**20B** (97 mg, 0.38 mmol) was dissolved in dichloromethane (4 mL) under an argon atmosphere, and *N*,*N*-diisopropylethylamine (0.2 mL, 1.1 mmol) was added and the mixture was stirred at 5 °C (ice/water). Trimethylacetyl chloride (56 μL, 0.45 mmol) was added dropwise, and the mixture was stirred at rt overnight. The reaction mixture was diluted in dichloromethane, washed with brine and saturated sodium bicarbonate, dried over magnesium sulfate, filtered, and the filtrate was evaporated. The residue was purified via flash silica chromatography (0:1–1:10 MeOH/EtOAc) to provide methyl 2-((2-(pyridin-3-yl)benzyl)amino)acetate (22 mg, 17%). UPLC-MS (short basic) ^*t*^R 0.74 (341 [M + H]^+^).

#### 2-(*N*-(2-(Pyridin-3-yl)benzyl)pivalamido)acetic Acid (**20C**)

Methyl 2-((2-(pyridin-3-yl)benzyl)amino)acetate (22 mg, 0.06 mmol) was dissolved in methanol (1 mL), and 2.5 M sodium hydroxide (0.2 mL, 0.50 mmol) was added and the mixture was stirred at rt overnight. The volatiles were removed, and then the residue was dissolved in water. The pH was adjusted carefully to 4 by the addition of 2 M HCl and extracted with ethyl acetate. The aqueous pH was again adjusted to 4. The aqueous layer was extracted with dichloromethane (repeated three times). The organics were washed with brine, dried over magnesium sulfate, filtered, and the filtrate was evaporated to provide **20C** as a yellow oil that was used directly in the next step (8 mg, 38%). UPLC-MS (short basic) ^*t*^R 0.49 (327 [M + H]^+^).

#### *N*-(2-Oxo-2-((2′-oxo-1,1′,2′,3-tetrahydrospiro[indene-2,3′-pyrrolo[2,3-*b*]pyridin]-5-yl)amino)ethyl)-*N*-(2-(pyridin-3-yl)benzyl)pivalamide (**20**)

**20C** (8 mg, 0.02 mmol), EDCI·HCl (7 mg, 0.03 mmol), and HOAt (5 mg, 0.08 mmol) were dissolved in dry DMF (0.5 mL). *N*,*N*-Diisopropylethylamine (13 μL, 0.07 mmol) and **20D** (7 mg, 0.03 mmol) were added, and the mixture was stirred at rt overnight. The crude was directly purified via MDAP (XBridge C18 19 × 150, 30–60% acetonitrile water with 0.1% ammonium hydroxide) to provide **20** (8 mg, 38%) as a pale yellow solid. ^1^H NMR (CDCl_3_, 300 MHz) δ 1.30 (s, 9H), 3.01 (dd, *J* = 15.8, 2.0 Hz, 2H), 3.59 (dd, *J* = 15.8, 2.8 Hz, 2H), 4.00 (s, 2H), 4.78 (s, 2H), 6.80 (dd, *J* = 7.3, 5.3 Hz, 1H), 7.05 (dd, *J* = 7.4, 1.5 Hz, 1H), 7.14–7.17 (m, 2H), 7.24–7.31 (m, 2H), 7.36–7.47 (m, 4H), 7.68 (dt, *J* = 7.9, 1.9 Hz, 1H), 8.11 (dd, *J* = 5.3, 1.5 Hz, 1H), 8.42 (br, 1H), 8.57 (d, *J* = 1.6 Hz, 1H), 8.63 (dd, *J* = 4.9, 1.6 Hz, 1H), 8.84 (br, 1H). UPLC-MS (short basic) ^*t*^R 2.04 (560 [M + H]^+^).

#### *N*-(2-Bromobenzyl)-*N*-(2-oxo-2-((2′-oxo-1,1′,2′,3-tetrahydrospiro[indene-2,3′-pyrrolo[2,3-*b*]pyridin]-5-yl)amino)ethyl)pivalamide (**21E**)

**21C** (3.11 g, 9.48 mmol), EDCI.HCl (2.5 g, 13.27 mmol), and HOAt (1.8 g, 13.27 mmol) were dissolved in dry DMF (60 mL). *N*,*N*-Diisopropylethylamine (5.0 mL, 28.44 mmol) and **21D** (2.38 g, 9.48 mmol) were added, and the mixture was stirred at rt for 18 h. The mixture was diluted with ethyl acetate (250 mL) and washed with saturated sodium bicarbonate and three times with brine. The organic layer was dried over magnesium sulfate, filtered, and the filtrate was evaporated. The residue was purified via flash silica chromatography (0–100% EtOAc in DCM) to provide **21E** (4.38 g, 83%) as a pale yellow solid. ^1^H NMR (CDCl_3_, 300 MHz) δ 1.31 (s, 9H), 3.04 (dd, *J* = 15.7, 6.4 Hz, 2H), 3.61 (dd, *J* = 15.8, 6.0 Hz, 2H), 4.07 (s, 2H), 4.91 (s, 2H), 6.81 (dd, *J* = 7.2, 5.4 Hz, 1H), 7.07 (d, *J* = 7.1 Hz, 1H), 7.12–7.24 (m, 3H), 7.34 (t, *J* = 7.5 Hz, 1H), 7.55–7.62 (m, 2H), 8.12 (dd, *J* = 5.2 Hz, 1H), 8.49 (s, 1H), 9.29 (s, 1H). UPLC-MS (short basic) ^*t*^R 0.84 (561, 563 [M + H]^+^).

#### *N*-(2-Cyanobenzyl)-*N*-(2-oxo-2-((2′-oxo-1,1′,2′,3-tetrahydrospiro[indene-2,3′-pyrrolo[2,3-*b*]pyridin]-5-yl)amino)ethyl)pivalamide (**21**)

**21E** (4.40 g, 7.84 mmol) was dissolved in dry DMF (88 mL) and was degassed by bubbling argon through the solution. Zinc(II) cyanide (1.66 g, 14.12 mmol) and tetrakis(triphenylphosphine)palladium(0) (1.8 g, 1.57 mmol) were added, and the mixture was stirred at 130 °C for 2 h. UPLC-MS indicated complete conversion. The heat was removed, and the mixture was stirred at rt for 18 h. The mixture was then diluted with ethyl acetate (400 mL) and washed twice with saturated sodium bicarbonate and three times with brine. The organic layer was dried over magnesium sulfate, filtered, and the filtrate was evaporated. The residue was triturated with diethyl ether to provide **21** (3.85 g, 96%) as an off-white solid. ^1^H NMR (CD_3_OD, 300 MHz) δ 1.24 (s, 9H), 3.03 (dd, *J* = 16.0, 10.7 Hz, 2H), 3.30 (dd, *J* = 16.0, 9.2 Hz, 2H), 4.04 (s, 2H), 5.02 (s, 2H), 6.82 (dd, *J* = 7.3, 5.3 Hz, 1H), 6.90 (d, *J* = 6.9 Hz, 1H), 7.10–7.55 (m, 7H), 8.00–8.05 (m, 2H), 9.75 (s, 1H), 10.94 (s, 1H).

#### *N*-(2-(Aminomethyl)benzyl)-*N*-(2-oxo-2-((2′-oxo-1,1′,2′,3-tetrahydrospiro[indene-2,3′-pyrrolo[2,3-*b*]pyridin]-5-yl)amino)ethyl)pivalamide (**12**)

**21** (2.3 g, 4.53 mmol) was dissolved in 15% ammonia in methanol (180 mL) under an argon atmosphere in an autoclave. Raney nickel (250 mg, 0.45 mmol) was added, and hydrogen was introduced to 500 psi. The vessel was stirred at 60 °C for 6 h and then at rt for 18 h. UPLC-MS showed 20% conversion, so extra Raney nickel (400 mg, 0.72 mmol) was added and hydrogen was reintroduced to 500 psi. The vessel was stirred at 60 °C for 6.5 h. UPLC-MS analysis showed 58% conversion. The mixture was decanted (from the nickel solids) and filtered through celite, washing with 15% ammonia in methanol, and the filtrate was evaporated. The residue was dissolved in 15% ammonia in methanol (180 mL) under an argon atmosphere in an autoclave. Raney nickel (400 mg, 0.72 mmol) was added, and hydrogen was reintroduced to 500 psi. The vessel was stirred at 50 °C for 6 h, then rt for 18 h, 55 °C for 6 h, rt for 42 h, and 55 °C for 8 h. The mixture was decanted (from the nickel solids) and filtered through celite, washing with 15% ammonia in methanol, and the filtrate was evaporated. The residue was purified via flash silica chromatography (EtOAc and then 5% MeOH in DCM, then 10–15% MeOH with ammonia in DCM) to provide **12** (240 mg, 10%) as a white powder after freeze-drying from an aqueous solution. ^1^H NMR (CD_3_OD, 300 MHz) δ 1.32 (s, 9H), 3.04 (dd, *J* = 15.9, 5.4 Hz, 2H), 3.49 (dd, *J* = 15.8, 9.8 Hz, 2H), 3.81 (s, 2H), 4.06 (br s, 2H), 4.93 (br s, 2H), 6.86 (dd, *J* = 7.3, 5.4 Hz, 1H), 7.09–7.41 (m, 7H), 7.53 (s, br, 1H), 8.03 (dd, *J* = 5.3, 1.5 Hz, 1H). UPLC-MS (long basic) ^*t*^R 1.79 (512 [M + H]^+^), 84% pure—contains 6% mono-*N-*methyl and 3% di-*N*-methylamine byproducts.

#### 2-((*N*-(2-Oxo-2-((2′-oxo-1,1′,2′,3-tetrahydrospiro[indene-2,3′-pyrrolo[2,3-*b*]pyridin]-5-yl)amino)ethyl)pivalamido)methyl)benzamide (**15**)

**21** (17 mg, 0.033 mmol) was dissolved in DMSO (1 mL). Water (0.17 mL) was added, followed by hydrogen peroxide solution (3 drops) and NaOH (2.8 mg, 0.07 mmol), and the mixture was stirred at rt for 2 h. The reaction mixture was quenched with ethyl acetate and water. The aqueous layer was extracted with ethyl acetate (repeated twice). The organics were washed with brine, dried over magnesium sulfate, filtered, and the filtrate was evaporated. The residue was purified twice via column chromatography (1:0 ethyl acetate/methanol to 15:1 ethyl acetate/methanol) to provide **15** (10.6 mg, 60%) as a pale yellow solid. ^1^H NMR (CD_3_OD, 300 MHz) δ 1.31 (s, 9H), 3.04 (dd, *J* = 15.8, 5.2 Hz, 2H), 3.48 (dd, *J* = 15.9, 7.5 Hz, 2H), 4.11 (s, 2H), 5.06 (s, 2H), 6.86 (dd, *J* = 7.3, 5.3 Hz, 1H), 7.11 (dd, *J* = 7.3, 1.6 Hz, 1H), 7.20 (d, *J* = 8.2 Hz, 2H), 7.30–7.39 (m, 3H), 7.44–7.55 (m, 3H), 8.03 (dd, *J* = 5.4, 1.6 Hz, 1H). UPLC-MS (short basic) ^*t*^R 1.65 (526 [M + H]^+^).

#### Methyl 2-((2-(2-(Diallylamino)ethyl)benzyl)amino)acetate (**16B**)

**16A** (116 mg, 0.516 mmol) was dissolved in methanol (2 mL), and then methyl glycinate hydrochloride (191 mg, 1.52 mmol) and sodium cyanoborohydride (55 mg, 0.88 mmol) were added and the mixture was stirred at rt for 18 h. The reaction mixture was poured into water, and the pH was adjusted to 4 with 2 M HCl before the mixture was washed twice with dichloromethane. The aqueous layer was basified with sodium carbonate and extracted twice with dichloromethane. This organic extract was dried over magnesium sulfate, filtered, and evaporated to provide **16B** (53 mg, 35%) as a colorless oil. ^1^H NMR (CDCl_3_, 300 MHz) δ 2.63–2.88 (m, 4H), 3.19 (d, *J* = 6.5 Hz, 4H), 3.44 (s, 2H), 3.73 (s, 3H), 3.79 (s, 2H), 5.11–5.24 (m, 4H), 5.80–5.95 (m, 2H), 7.12–7.32 (m, 4H). UPLC-MS (short basic) ^*t*^R 0.84 (303 [M + H]^+^), 80% pure.

#### Methyl 2-(*N*-(2-(2-(Diallylamino)ethyl)benzyl)pivalamido)acetate

**16B** (54 mg, 0.18 mmol) was dissolved in dichloromethane (1 mL) under an argon atmosphere, and *N*,*N*-diisopropylethylamine (93 μL, 0.53 mmol) was added. Trimethylacetyl chloride (26 μL, 0.21 mmol) was added dropwise, and the mixture was stirred at rt for 4 days. The mixture was poured into saturated sodium bicarbonate and extracted three times with dichloromethane. The organic extracts were evaporated to provide methyl 2-(*N*-(2-(2-(diallylamino)ethyl)benzyl)pivalamido)acetate (68 mg, 99%) as a colorless oil. UPLC-MS (short basic) ^*t*^R 0.96 (387 [M + H]^+^), 89% pure.

#### 2-(*N*-(2-(2-(Diallylamino)ethyl)benzyl)pivalamido)acetic Acid (**16C**)

Methyl 2-(*N*-(2-(2-(diallylamino)ethyl)benzyl)pivalamido)acetate (68 mg, 0.176 mmol) was dissolved in methanol (1 mL), and then 2.5 M sodium hydroxide (0.22 mL, 0.55 mmol) was added and the mixture was stirred at rt for 18 h. The volatiles were removed, the material was diluted with water, and the pH was adjusted to 5 with 2 M HCl. This was then concentrated to dryness to provide **16C** (assume 0.176 mmol) as a glass that was used directly in the next step.

#### *N*-(2-(2-(Diallylamino)ethyl)benzyl)-*N*-(2-oxo-2-((2′-oxo-1,1′,2′,3-tetrahydrospiro[indene-2,3′-pyrrolo[2,3-*b*]pyridin]-5-yl)amino)ethyl)pivalamide (**16E**)

**16C** (∼0.176 mmol), EDCI.HCl (53 mg, 0.28 mmol), and HOAt (38 mg, 0.28 mmol) were dissolved in dry DMF (1 mL). *N*,*N*-Diisopropylethylamine (0.11 mL, 0.64 mmol) and **16D** (44.5 mg, 0.177 mmol) were added, and the mixture was stirred at rt for 18 h. The mixture was poured into saturated sodium bicarbonate and extracted three times with ethyl acetate. The combined organic layers were washed with brine, dried over magnesium sulfate, filtered, and the filtrate was evaporated. The residue was purified via flash silica chromatography (EtOAc) to provide **16E** (77 mg, 72%) as a pale yellow glass. ^1^H NMR (CDCl_3_, 400 MHz) δ 1.32 (s, 9H), 2.61–2.79 (m, 4H), 3.03 (dd, *J* = 15.8, 8.8 Hz, 2H), 3.17 (d, *J* = 6.1 Hz, 4H), 3.61 (dd, *J* = 15.5, 8.3 Hz, 2H), 4.02 (br s, 2H), 4.90 (s, 2H), 5.07–5.23 (m, 4H), 5.78–5.90 (m, 2H), 6.80 (dd, *J* = 7.3, 5.4 Hz, 1H), 7.03–7.27 (m, 7H), 7.56 (s, 1H), 8.15 (br s, 1H), 8.45 (s, 1H). UPLC-MS (long basic) ^*t*^R 2.65 (606 [M + H]^+^), 98% pure.

#### *N*-(2-(2-Aminoethyl)benzyl)-*N*-(2-oxo-2-((2′-oxo-1,1′,2′,3-tetrahydrospiro[indene-2,3′-pyrrolo[2,3-*b*]pyridin]-5-yl)amino)ethyl)pivalamide (**16**)

**16E** (77 mg, 0.127 mmol) and *N*,*N*′-dimethylbarbituric acid (125 mg, 0.801 mmol) were dissolved in dry degassed dichloromethane (2 mL) and degassed again. Tetrakis(triphenylphosphine)palladium(0) (11.4 mg, 0.010 mmol) was added, and the mixture was stirred at 35 °C for 2 h and at rt for 18 h. UPLC-MS analysis showed incomplete reaction. Tetrakis(triphenylphosphine)palladium(0) (13 mg, 0.011 mmol) was added, and the mixture was stirred at 35 °C for 3.5 h. UPLC-MS still showed incomplete conversion. The mixture was diluted with dichloromethane and saturated sodium bicarbonate, and layers were separated. The aqueous layer was extracted with dichloromethane. The combined organic layers were dried over magnesium sulfate, filtered, and the filtrate was evaporated. The aqueous layer was back-extracted twice with ethyl acetate, the combined organic layers were dried over magnesium sulfate, filtered, and the filtrate was evaporated. The extracted residues were combined and shown to contain monoallyl byproduct. The product was still in the aqueous layer, which was evaporated and purified using a Biotage Isolera (18 g, C18 Ultra cartridge, 60–80% acetonitrile/water with pH 10 buffer) to provide crude **16**. This was further purified via MDAP (XBridge C18 19 × 150, 35–50% acetonitrile water with 0.1% ammonium hydroxide) to provide **16** (19.4 mg, 29%) as a pale yellow glass. ^1^H NMR (CDCl_3_, 300 MHz) δ 1.33 (s, 9H), 2.78 (t, *J* = 6.9 Hz, 2H), 2.93–3.07 (m, 4H), 3.59 (d, *J* = 15.5 Hz, 2H), 4.05 (br s, 2H), 4.97 (br s, 2H), 6.80 (dd, *J* = 7.3, 5.3 Hz, 1H), 7.03–7.26 (m, 8H), 7.53 (s, 1H), 8.11 (dd, *J* = 5.3, 1.4 Hz, 1H), 8.62 (s, 1H). UPLC-MS (long basic) ^*t*^R 1.79 (526 [M + H]^+^), 94% pure.

#### *tert*-Butyl 8-(((2-Methoxy-2-oxoethyl)amino)methyl)-3,4-dihydroisoquinoline-2(1*H*)-carboxylate (**17B**)

**17A** (80 mg, 0.306 mmol) was dissolved in dichloromethane (5 mL), and *N*,*N*-diisopropylethylamine (0.20 mL, 1.22 mmol) and glycine methyl ester hydrochloride (115 mg, 0.918 mmol) were added, followed by magnesium sulfate. The mixture was stirred at rt for 4 h. Sodium triacetoxyborohydride (97 mg, 0.46 mmol) was added, and stirring was continued at rt for 72 h. The reaction mixture was poured into saturated sodium bicarbonate and extracted with dichloromethane. The organic extract was dried over sodium sulfate, filtered, and evaporated. UPLC-MS indicated a 1:1 mixture of imine and amine. Repeating conditions with sodium triacetoxyborohydride in dichloromethane did not improve the ratio. The residue was dissolved in methanol (10 mL), cooled on ice/water, and sodium borohydride (7 mg, 0.18 mmol) was added, and the mixture was stirred at rt for 1.5 h. The mixture was diluted with ethyl acetate and washed with saturated sodium bicarbonate. The aqueous layer was extracted with ethyl acetate, and the organic extracts were washed with water, dried over sodium sulfate, filtered, and evaporated to provide **17B** (150 mg, quantitative yield) as a yellow oil that was used directly in the next step. UPLC-MS (short basic) ^*t*^R 0.83 (335 [M + H]^+^).

#### *tert*-Butyl 8-((*N*-(2-Methoxy-2-oxoethyl)pivalamido)methyl)-3,4-dihydroisoquinoline-2(1*H*)-carboxylate

**17B** (148 mg, ∼0.407 mmol) was dissolved in dichloromethane (3 mL) under an argon atmosphere, and *N*,*N*-diisopropylethylamine (140 μL, 0.80 mmol) was added. Trimethylacetyl chloride (50 μL, 0.40 mmol) was added dropwise, and the mixture was stirred at rt for 3 h after which time UPLC-MS indicated that amine had been completely consumed. The mixture was poured into saturated sodium bicarbonate and extracted three times with dichloromethane. The organic extracts were dried over sodium sulfate, filtered, and the filtrate was evaporated. The residue was purified via flash silica SPE (5 g SiO_2_ SPE, 15% EtOAc in DCM) to provide *tert*-butyl 8-((*N*-(2-methoxy-2-oxoethyl)pivalamido)methyl)-3,4-dihydroisoquinoline-2(1*H*)-carboxylate (35 mg, 20%) as a colorless gum. UPLC-MS (short basic) ^*t*^R 0.93 (419 [M + H]^+^), 80% pure.

#### 2-(*N*-((2-(*tert*-Butoxycarbonyl)-1,2,3,4-tetrahydroisoquinolin-8-yl)methyl)pivalamido)-acetic Acid (**17C**)

*tert*-Butyl 8-((*N*-(2-methoxy-2-oxoethyl)pivalamido)methyl)-3,4-dihydroisoquinoline-2(1*H*)-carboxylate (35 mg, 0.084 mmol) was dissolved in methanol (3 mL), and 2.5 M sodium hydroxide (50 μL, 0.125 mmol) was added and the mixture was stirred at rt for 18 h. UPLC-MS indicated incomplete hydrolysis, so a further 2.5 M sodium hydroxide (50 μL, 0.125 mmol) was added, and the mixture was stirred at rt for 72 h. The reaction was diluted with ethyl acetate and washed with saturated ammonium chloride. The aqueous layer was extracted twice with ethyl acetate. The organic extracts were dried over sodium sulfate, filtered, and the filtrate was evaporated to provide **17C** (∼0.084 mmol) as a glass, which was used directly in the next step. UPLC-MS (short basic) ^*t*^R 0.59 (405 [M + H]^+^).

#### *tert*-Butyl 8-((*N*-(2-Oxo-2-((2′-oxo-1,1′,2′,3-tetrahydrospiro[indene-2,3′-pyrrolo[2,3-*b*]pyridin]-5-yl)amino)ethyl)pivalamido)methyl)-3,4-dihydroisoquinoline-2(1*H*)-carboxylate (**17E**)

**17C** (35 mg, 0.084 mmol), EDCI.HCl (19 mg, 0.101 mmol), and HOAt (14 mg, 0.101 mmol) were dissolved in dry DMF (2 mL). *N*,*N*-Diisopropylethylamine (35 μL, 0.20 mmol) and **17D** (21 mg, 0.084 mmol) were added, and the mixture was stirred at rt for 18 h. The mixture was poured into saturated sodium bicarbonate, and the aqueous layer was extracted three times with ethyl acetate. The organic extract was washed three times with water, dried over sodium sulfate, filtered, and the filtrate was evaporated. The residue was purified via SPE (2 g SiO_2_ EtOAc) to provide **17E** (30 mg, 56%) as a colorless glass. UPLC-MS (short basic) ^*t*^R 0.86 (638 [M + H]^+^).

#### *N*-(2-Oxo-2-((2′-oxo-1,1′,2′,3-tetrahydrospiro[indene-2,3′-pyrrolo[2,3-*b*]pyridin]-5-yl)amino)ethyl)-*N*-((1,2,3,4-tetrahydroisoquinolin-8-yl)methyl)pivalamide (**17**)

**17E** (30 mg, 0.047 mmol) was dissolved in dichloromethane (3 mL). Trifluoroacetic acid (0.3 mL) was added, and the solution was stirred at rt for 45 min. The mixture was poured into saturated sodium bicarbonate, and the aqueous layer was extracted three times with dichloromethane. The organic extract was dried over sodium sulfate, filtered, and the filtrate was evaporated. The residue was purified via SPE (2 g SiO_2_ 10% MeOH in EtOAc and then 10–20% MeOH in DCM) to provide **17** (12 mg, 48%) as a colorless glass. ^1^H NMR (CD_3_OD, 300 MHz) δ 1.31 (s, 9H), 2.88 (t, *J* = 5.9 Hz, 2H), 3.01–3.12 (m, 4H), 3.49 (dd, *J* = 15.8, 7.6 Hz, 2H), 3.94 (s, 2H), 4.10 (br s, 2H), 4.74 (br s, 2H), 6.86 (dd, *J* = 7.3, 5.4 Hz, 1H), 6.99 (d, *J* = 7.0 Hz, 1H), 7.04–7.23 (m, 4H), 7.34 (dd, *J* = 8.2, 1.5 Hz, 1H), 7.52 (s, 1H), 8.03 (dd, *J* = 5.3, 1.6 Hz, 1H). UPLC-MS (short basic) ^*t*^R 0.66 (538 [M + H]^+^), 99% pure.

#### Ethyl 2-((2-((*tert*-Butoxycarbonyl)amino)benzyl)amino)acetate (**11B**)

**11A** (100 mg, 0.45 mmol), ethyl bromoacetate (38 μL, 0.34 mmol), and *N*,*N*-diisopropylethylamine (157 μL, 0.90 mmol) were mixed in DMF (1 mL) and stirred at rt for 2 h, after which the reaction was complete by UPLC-MS. The mixture was diluted with ethyl acetate and washed with water. The aqueous layer was extracted with ethyl acetate. The organics were washed with brine, dried over magnesium sulfate, filtered, and the filtrate was evaporated to provide **11B** (119 mg, 86%) as a yellow gum. ^1^H NMR (CDCl_3_, 300 MHz) δ 1.26 (t, *J* = 4.6 Hz, 3H), 1.52 (s, 9H), 3.36 (s, 2H), 3.84 (s, 2H), 4.21 (q, *J* = 7.2 Hz, 2H), 6.93 (dt, *J* = 5.8, 1.4 Hz, 1H), 7.06 (dd, *J* = 7.7, 1.6 Hz, 1H), 7.23–7.31 (m, 1H), 7.98 (br d, 1H), 9.14 (br s, 1H).

#### Ethyl 2-(*N*-(2-((*tert*-Butoxycarbonyl)amino)benzyl)pivalamido)acetate

**11B** (119 mg, 0.39 mmol) was dissolved in dichloromethane (5 mL), and *N*,*N*-diisopropylethylamine (204 μL, 1.17 mmol) and trimethylacetyl chloride (58 μL, 0.47 mmol) were added and the mixture was stirred at rt for 2 h. UPLC-MS showed little reaction, so further *N*,*N*-diisopropylethylamine (204 μL, 1.17 mmol) and trimethylacetyl chloride (58 μL, 0.47 mmol) were added. After an additional 2 h, UPLC-MS showed complete reaction. The mixture was poured into water, and the aqueous layer was extracted with dichloromethane. The organics were dried over magnesium sulfate, filtered, and evaporated. The residue was purified via flash chromatography (4:1 heptane/ethyl acetate) to provide the intermediate of **11C** (99 mg, 65%) as a colorless oil. ^1^H NMR (CDCl_3_, 300 MHz) δ 1.25 (t, *J* = 7.1 Hz, 3H), 1.30 (s, 9H), 1.51 (s, 9H), 4.00 (s, 2H), 4.18 (q, *J* = 7.1 Hz, 2H), 4.72 (s, 2H), 6.96–7.10 (m, 2H), 7.26–7.33 (m, 1H), 7.99 (br s, 1H). UPLC-MS (short CSH 2–50%) ^*t*^R 1.50 (415 [M + Na]^+^), 95% pure.

#### 2-(*N*-(2-((*tert*-Butoxycarbonyl)amino)benzyl)pivalamido)acetic Acid **11C**

An intermediate of **7C** (99 mg, 0.25 mmol) was dissolved in methanol (1.5 mL), and 2.5 M sodium hydroxide (0.25 mL, 0.625 mmol) was added and the mixture was heated at reflux for 2 h. The mixture was poured into water, and the pH was adjusted carefully to 4 by the addition of 2 M HCl, and the mixture was extracted with ethyl acetate. The aqueous pH was again adjusted to 4, and the mixture was extracted with ethyl acetate. The organics were washed with brine, dried over magnesium sulfate, filtered, and the filtrate was evaporated to provide **11C** (80 mg, 88%) as a colorless solid. ^1^H NMR (CDCl_3_, 300 MHz) δ 1.31 (s, 9H), 1.50 (s, 9H), 4.03 (s, 2H), 4.75 (s, 2H), 7.05–7.14 (m, 2H), 7.26–7.34 (m, 2H), 7.82 (br s, 1H). UPLC-MS (short CSH 2–50%) ^*t*^R 1.28 (363 [M + Na]^+^), 95% pure.

#### *tert*-Butyl (2-((*N*-(2-Oxo-2-((2′-oxo-1,1′,2′,3-tetrahydrospiro[indene-2,3′-pyrrolo[2,3-*b*]pyridin]-5-yl)amino)ethyl)pivalamido)methyl)phenyl)carbamate (**11E**)

**11C** (80 mg, 0.22 mmol), EDCI.HCl (50 mg, 0.26 mmol), and HOAt (35 mg, 0.26 mmol) were dissolved in dry DMF (4 mL). *N*,*N*-Diisopropylethylamine (115 μL, 0.66 mmol) and **11D** (55 mg, 0.22 mmol) were added, and the mixture was stirred at rt for 4 h. The mixture was diluted with ethyl acetate and washed with saturated sodium bicarbonate. The aqueous layer was extracted twice with ethyl acetate. The combined organics were washed with brine, dried over magnesium sulfate, filtered, and the filtrate was evaporated. The residue was purified via flash silica chromatography (1:1 heptane/acetone) to provide **11E** (110 mg, 84%) as a colorless solid. ^1^H NMR (CDCl_3_, 300 MHz) δ 1.36 (s, 9H), 1.51 (s, 9H), 3.03 (dd, *J* = 15.8, 2.3 Hz, 2H), 3.60 (dd, *J* = 16.0, 3.6 Hz, 2H), 4.06 (br s, 2H), 4.83 (s, 2H), 6.81 (dd, *J* = 7.4, 5.3 Hz, 1H), 7.04–7.21 (m, 5H), 7.26–7.34 (m, 1H), 7.51 (s, 1H), 7.75 (br, s, 1H), 8.12 (dd, *J* = 5.3, 1.6 Hz, 2H). UPLC-MS (short CSH 2–50%) ^*t*^R 1.31 (498 [M-Boc + H]^+^), 88% pure.

#### *N*-(2-Aminobenzyl)-*N*-(2-oxo-2-((2′-oxo-1,1′,2′,3-tetrahydrospiro[indene-2,3′-pyrrolo[2,3-*b*]pyridin]-5-yl)amino)ethyl)pivalamide (**11**)

**11E** (20 mg, 0.033 mmol) was dissolved in dichloromethane (1 mL). Trifluoroacetic acid (0.05 mL) was added, and the solution was stirred at rt for 7 h. The mixture was poured into water, and the aqueous layer was extracted three times with dichloromethane. The organic extract was dried over magnesium sulfate, filtered, and the filtrate was evaporated. The residue was purified via prep-HPLC (HP C18, ID 22 mm, length 150 mm, flow rate 16 mL/min: 5–50% MeCN/water/0.1% trifluoroacetyl (TFA) over 20 min) to provide **11** (10.3 mg, 48%) as a colorless glass (TFA salt). ^1^H NMR (CD_3_OD, 400 MHz) δ 1.34 (s, 9H), 3.09 (dd, *J* = 15.8, 6.6 Hz, 2H), 3.53 (dd, *J* = 15.8, 2.8 Hz, 2H), 4.17 (br s, 2H), 4.61 (br s, 1H), 4.75 (br s, 2H), 6.72 (t, *J* = 7.3 Hz, 1H), 6.77 (d, *J* = 7.9 Hz, 1H), 6.91 (dd, *J* = 7.4, 5.4 Hz, 1H), 7.00 (d, *J* = 7.5 Hz, 1H), 7.06–7.11 (m, 1H), 7.16 (dd, *J* = 7.4, 1.5 Hz, 1H), 7.25 (d, *J* = 8.2 Hz, 1H), 7.39 (dd, *J* = 8.1, 1.6 Hz, 1H), 7.57 (br s, 1H), 8.07 (dd, *J* = 5.3, 1.4 Hz, 1H). HPLC (25 min acidic) ^*t*^R 12.28, 99% pure. MS 498 [M + H]^+^.

#### *N*-(2-(Guanidinomethyl)benzyl)-*N*-(2-oxo-2-((2′-oxo-1,1′,2′,3-tetrahydrospiro[indene-2,3′-pyrrolo[2,3-*b*]pyridin]-5-yl)amino)ethyl)pivalamide (**24**)

**12** (15 mg, 0.03 mmol) and 4-benzyl-3,5-dimethyl-1*H*-pyrazole-1-carboximidamide hydrochloride (prepared according to the literature;^47^ 30 mg, 0.117 mmol) and triethylamine (15 mg, 0.15 mmol) were added to tetrahydrofuran (0.3 mL) and acetonitrile (0.3 mL), and the mixture was heated at 90 °C under microwave irradiation for 1 h. The mixture was diluted with methanol and purified directly by prep-HPLC (HP C18, ID 22 mm, length 150 mm, flow 16 mL/min: 5–45% MeCN water/acetonitrile 0.1% TFA over 20 min) to provide the desired **24** (8.9 mg, 55%) as a colorless glass (TFA salt). ^1^H NMR (CD_3_OD, 400 MHz) δ 1.36 (s, 9H), 3.11 (dd, *J* = 15.9, 2.8 Hz, 2H), 3.53 (dd, *J* = 15.9, 8.9 Hz, 2H), 4.29 (br s, 2H), 4.45–4.50 (m, 2H), 4.85 (br s, 2H), 6.93 (dd, *J* = 7.3, 5.4 Hz, 1H), 7.19 (dd, *J* = 7.4, 1.5 Hz, 1H), 7.24 (d, *J* = 8.2 Hz, 1H), 7.28–7.42 (m, 5H), 7.52 (br s, 1H), 7.80–7.85 (m, 1H), 8.08 (dd, *J* = 5.4, 1.5 Hz, 1H). HPLC: 98% pure. MS: 554 [M + H]^+^.

#### 2-(1,1-Dimethoxyethyl)benzaldehyde (**29E**)

**29A** (830 mg, 3.39 mmol) was dissolved in dry tetrahydrofuran (10 mL) under an argon atmosphere and cooled on dry ice/acetone. To this was added a solution of *n*-butyllithium (2.04 mL, 5.09 mmol, 2.5 M in hexanes) dropwise so that the internal temperature stayed below −60 °C (10 min addition). The reaction was stirred on dry ice/acetone for 60 min. To this was added DMF (0.525 mL, 6.78 mmol) in one portion. The mixture was stirred on dry ice/acetone for 60 min before being allowed to warm to rt over 18 h. Water was added, and the mixture was extracted three times with ethyl acetate. The combined organic extracts were washed with brine, dried over magnesium sulfate, filtered, and the filtrate was evaporated to provide **29E** (634 mg, 96%) as a straw-colored oil. ^1^H NMR (CDCl_3_, 300 MHz) δ 1.70 (s, 3H), 3.23 (s, 6H), 7.36–7.44 (m, 1H), 7.50–7.57 (m, 1H), 7.64 (dd, *J* = 7.9, 1.3 Hz, 1H), 7.86 (dd, *J* = 7.7, 1.4 Hz, 1H), 10.64 (s, 1H).

#### Methyl 2-((2-(1,1-Dimethoxyethyl)benzyl)amino)acetate (**29B**)

**29E** (634 mg, 3.26 mmol) was dissolved in dichloromethane (25 mL) under an argon atmosphere. *N*,*N*-Diisopropylethylamine (1.14 mL, 6.52 mmol) was added followed by methyl glycinate hydrochloride (777 mg, 6.19 mmol) and magnesium sulfate (excess). The mixture was stirred at rt for 1 h. Sodium triacetoxyborohydride (1.1 g, 5.2 mmol) was added, and the mixture was stirred at rt for 18 h. The mixture was poured into water, and the aqueous layer was extracted three times with dichloromethane. The combined organic extracts were washed with brine, dried over magnesium sulfate, filtered, and the filtrate was evaporated to provide **29B** (717 g, 82%) as a pale straw-colored gum. ^1^H NMR (CDCl_3_, 300 MHz) δ 1.58 (s, 3H), 3.23 (s, 6H), 3.50 (s, 2H), 3.72 (s, 3H), 3.98 (s, 2H), 7.26–7.30 (m, 2H), 7.37–7.42 (m, 1H), 7.52–7.57 (m, 1H).

#### Methyl 2-(*N*-(2-(1,1-Dimethoxyethyl)benzyl)pivalamido)acetate

**29B** (685 mg, 2.56 mmol) was dissolved in dichloromethane (40 mL) under an argon atmosphere, and *N*,*N*-diisopropylethylamine (1.34 mL, 7.68 mmol) was added. Trimethylacetyl chloride (0.38 mL, 3.07 mmol) was added dropwise. The mixture was stirred at rt for 3 h after which the reaction had reached completion, as judged by TLC analysis. The mixture was poured into water, and the aqueous layer was extracted three times with dichloromethane. The combined organic extracts were washed with brine, dried over magnesium sulfate, filtered, and the filtrate was evaporated to provide methyl 2-(*N*-(2-(1-aminoethyl)benzyl)pivalamido)acetate (1.012 g, quantitative yield) as a yellow gum. ^1^H NMR (CDCl_3_, 300 MHz) δ 1.32 (s, 9H), 1.52 (s, 3H), 3.20 (s, 6H), 3.72 (s, 3H), 5.02 (br s, 2H), 7.25–7.32 (m, 3H), 7.60 (dd, *J* = 7.0, 2.2 Hz, 1H), one signal collapsed, not visible.

#### 2-(*N*-(2-(1,1-Dimethoxyethyl)benzyl)pivalamido)acetic Acid (**29C**)

Methyl 2-(*N*-(2-(1-aminoethyl)benzyl)pivalamido)acetate (500 mg, 1.40 mmol) was dissolved in methanol (5 mL), and 2.5 M sodium hydroxide (0.84 mL, 2.1 mmol) was added. The mixture was stirred at rt for 3 h, after which the reaction had reached completion, as judged by TLC analysis. The mixture was diluted with water, and the pH was adjusted very carefully to pH 4 with 10% potassium hydrogen sulfate. Once at pH 4, the aqueous layer was extracted twice with ethyl acetate. The combined organic extracts were washed with brine, dried over magnesium sulfate, filtered, and evaporated carefully (30 °C water bath, not to dryness). **29C** was used directly in the next step as the compound is not stable.

#### *N*-(2-(1,1-Dimethoxyethyl)benzyl)-*N*-(2-oxo-2-((2′-oxo-1,1′,2′,3-tetrahydrospiro[indene-2,3′-pyrrolo[2,3-*b*]pyridin]-5-yl)amino)ethyl)pivalamide (**29F**)

**29C** (∼1.40 mmol) was dissolved in DMF (15 mL) under an argon atmosphere, and *N*,*N*-diisopropylethylamine (0.73 mL, 4.2 mmol) was added. EDCI.HCl (322 mg, 1.68 mmol) and HOAt (229 mg, 1.68 mmol) were added, followed by **29D** (387 mg, 1.54 mmol). The mixture was stirred at rt for 3 days. The mixture was poured into saturated sodium bicarbonate. The aqueous layer was extracted three times with ethyl acetate. The combined organic extracts were washed three times with water, dried over sodium sulfate, filtered, and the filtrate was evaporated. The residue was purified via column chromatography (300 mL silica, 2:1 heptane/acetone) to provide **29F** (247 mg, 31%) as a colorless glass. ^1^H NMR (CDCl_3_, 300 MHz) δ 1.34 (s, 9H), 1.59 (s, 3H), 2.94–3.08 (m, 2H), 3.22 (s, 6H), 3.53–3.67 (m, 4H), 5.10 (br s, 2H), 6.58–6.63 (m, 1H), 6.75–6.84 (m, 1H), 7.02–7.07 (m, 2H), 7.15–7.23 (m, 1H), 7.26–7.37 (m, 3H), 8.05 (br s, 1H), 8.07–8.12 (m, 1H), 8.62 (br s, 1H).

#### *N*-(2-Acetylbenzyl)-*N*-(2-oxo-2-((2′-oxo-1,1′,2′,3-tetrahydrospiro[indene-2,3′-pyrrolo[2,3-*b*]pyridin]-5-yl)amino)ethyl)pivalamide (**29G**)

**29F** (247 mg, 0.43 mmol) was dissolved in acetone (15 mL), and *p*-toluene sulfonic acid monohydrate (89 mg, 0.47 mmol) was added. The mixture was stirred at rt for 4 h, at which point further *p*-toluene sulfonic acid monohydrate (33 mg, 0.17 mmol) was added and the reaction was stirred for a further 1 h. The mixture was poured into saturated sodium bicarbonate. The aqueous layer was extracted three times with ethyl acetate. The combined organic extracts were washed with brine, dried over sodium sulfate, filtered, and the filtrate was evaporated to provide **29G** (89 mg, 39%) as a colorless solid. ^1^H NMR (CDCl_3_, 300 MHz) δ 1.31 (s, 9H), 2.64 (s, 3H), 3.04 (dd, *J* = 15.8, 7.4, 2H), 3.62 (dd, *J* = 15.7, 6.2 Hz, 2H), 4.05 (s, 2H), 5.23 (s, 2H), 6.82 (dd, *J* = 7.3, 5.3 Hz, 1H), 7.07 (dd, *J* = 7.3, 1.6 Hz, 1H), 7.16–7.31 (m, 3H), 7.42 (t, *J* = 7.2 Hz, 1H), 7.52–7.56 (m, 2H), 7.89 (d, *J* = 7.5 Hz, 1H), 8.11 (dd, *J* = 5.3, 1.5 Hz, 1H), 8.63 (br s, 1H). UPLC-MS ^*t*^R 0.75 (524 [M + H]^+^), 90% pure.

#### *N*-(2-(1-Aminoethyl)benzyl)-*N*-(2-oxo-2-((2′-oxo-1,1′,2′,3-tetrahydrospiro[indene-2,3′-pyrrolo[2,3-*b*]pyridin]-5-yl)amino)ethyl)pivalamide (**29**)

**Scheme 5:**
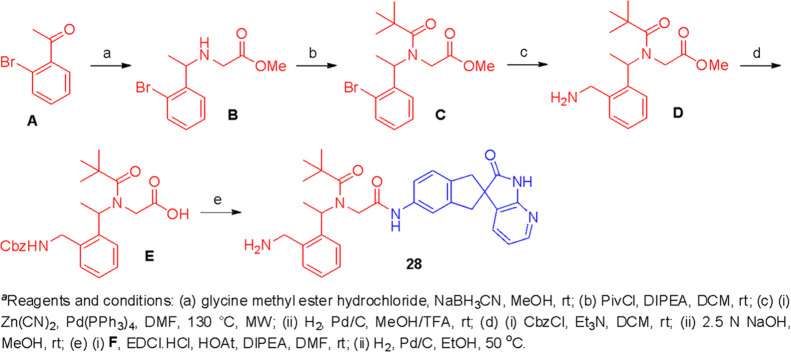
Synthesis of **28**

**29G** (83 mg, 0.17 mmol) was dissolved in methanol (3.5 mL), and ammonium acetate (131 mg, 1.7 mmol) and sodium cyanoborohydride (21 mg, 0.34 mmol) were added. The mixture was stirred at reflux for 18 h. Extra ammonium acetate (131 mg, 1.7 mmol) and sodium cyanoborohydride (21 mg, 0.34 mmol) were added, and the mixture was stirred at 50 °C for 72 h. The mixture was poured into water, and the aqueous layer was extracted with dichloromethane. The organic extract was evaporated, and the residue was purified via prep-HPLC (XBridge C18, ID 19 mm, length 150 mm, flow rate 20 mL/min: 40–60% MeCN in pH 10 [NH_4_HCO_3_ with NH_4_OH] over 8 min) to provide **29** (15 mg, 17%) as a colorless solid. ^1^H NMR (CD_3_OD, 300 MHz) δ 1.29–1.37 (m, 12H), 3.05 (dd, *J* = 15.6, 6.1 Hz, 2H), 3.49 (dd, *J* = 15.5, 10.6 Hz, 2H), 3.38–4.37 (m, 2H), 4.86–5.00 (m, 2H), 6.84–6.89 (m, 1H), 7.09–7.16 (m, 2H), 7.18–7.27 (m, 2H), 7.29–7.35 (m, 2H), 7.49–7.56 (m, 2H), 8.01–8.05 (m, 1H). UPLC-MS (long run) ^*t*^R 1.86 (526 [M + H]^+^), 99% pure (Scheme 5).

#### Methyl 2-((1-(2-Bromophenyl)ethyl)amino)acetate (**28B**)

**28A** (5 g, 25 mmol) was dissolved in methanol (9.8 mL), and then methyl glycinate hydrochloride (15.69 g, 125 mmol) and sodium cyanoborohydride (3.14 g, 62.84 mmol) were added and the mixture was stirred at rt over the weekend. The reaction mixture was poured into water, and the pH was adjusted to 4 with 2 M HCl before the mixture was washed twice with dichloromethane. The aqueous layer was basified with sodium carbonate and extracted twice with dichloromethane. This organic extract was dried over magnesium sulfate, filtered, and evaporated. The residue was purified by Isolera (acetonitrile/NH_4_COOH buffer pH =10) to provide **28B** (1.857 g, 27%). UPLC-MS (short basic) ^*t*^R 0.88 (273 [M + H]^+^).

#### Methyl 2-(*N*-(1-(2-Bromophenyl)ethyl)pivalamido)acetate **28C**

**28B** (1.86 g, 6.8 mmol) was dissolved in dichloromethane (50 mL) under an argon atmosphere, and *N*,*N*-diisopropylethylamine (3.55 mL, 20.4 mmol) was added and the mixture was stirred at 5 °C (ice/water). Trimethylacetyl chloride (1 mL, 8.16 mmol) was added dropwise, and the mixture was stirred at rt for 4 h. The reaction mixture was diluted in dichloromethane, washed with brine and saturated sodium bicarbonate, dried over magnesium sulfate, filtered, and the filtrate was evaporated. The residue was purified by flash chromatography eluting with heptane/acetone = 4:1 to provide the intermediate of **28C** (2.2 g, 91%). UPLC-MS (short basic) ^*t*^R 0.98 (357 [M + H]^+^).

#### Methyl 2-(*N*-(1-(2-Cyanophenyl)ethyl)pivalamido)acetate

**28C** (100 mg, 0.28 mmol) was dissolved in dry DMF (3 mL) and was degassed by bubbling argon through the solution. Zinc(II) cyanide (59 mg, 0.5 mmol) and tetrakis(triphenylphosphine)palladium(0) (65 mg, 0.056 mmol) were added, and the mixture was stirred at 130 °C for 1 h. The mixture was diluted with ethyl acetate and washed twice with saturated sodium bicarbonate and three times with brine. The organic layer was dried over magnesium sulfate, filtered, and the filtrate was evaporated. The crude was directly purified using a Biotage Isolera (C18 Ultra cartridge, 0–20% acetone/heptane) to provide the intermediate of **28D** (67 mg, 79%). ^1^H NMR (CDCl_3_, 300 MHz) δ 1.24 (s, 9H), 1.68 (s, 3H), 3.74 (s, 3H), 4.52 (d, *J* = 17.3 Hz, 1H), 7.31–7.45 (m, 2H), 7.58 (t, *J* = 7.6 Hz, 1H), 7.67 (d, *J* = 7.9 Hz, 1H). UPLC-MS (short basic) ^*t*^R 0.79 (303.2 [M + H]^+^).

#### Methyl 2-(*N*-(1-(2-(Aminomethyl)phenyl)ethyl)pivalamido)acetate (**28D**)

An intermediate of **28D** (67 mg, 0.22 mmol) was dissolved in a mixture of methanol (4.5 mL) and TFA (0.5 mL). Palladium-on-carbon (10% wet, 30 mg) was added, the vessel was sealed, and an atmosphere of hydrogen was introduced using a balloon. The mixture was stirred at rt for 5 h. The reaction was filtered through celite, washed with methanol, and the filtrate was evaporated to provide **28D**. UPLC-MS (long basic) ^*t*^R 0.68 (307 [M + H]^+^), 84% pure.

#### Methyl 2-(*N*-(1-(2-((((Benzyloxy)carbonyl)amino)methyl)phenyl)ethyl)pivalamido)acetate

**28D** (67 mg, 0.22 mmol) in dichloromethane (2 mL) and triethylamine (92 μL, 0.66 mmol) was added to benzyl chloroformate (34 μL, 0.24 mmol). The mixture was stirred at rt for 4 h, quenched by adding water, and extracted with dichloromethane. The combined organic phases were dried over sodium sulfate. After filtration and concentration, the residue was purified by silica gel column chromatography, eluting with heptane/acetone = 4:1 to provide the intermediate of **28E** (62 mg, 64% yield). ^1^H NMR (CDCl_3_, 300 MHz) δ 1.21 (s, 9H), 1.42 (d, *J* = 6.5 Hz, 3H), 3.34 (s, 3H), 4.01 (d, *J* = 18.0 Hz, 1H), 4.20 (d, *J* = 18.0 Hz), 4.39 (d, *J* = 5.5 Hz, 2H), 5.12 (s, 2H), 5.95 (s, 1H), 6.18 (s, 1H), 7.23–7.38 (m, 9H). UPLC-MS (short basic) ^*t*^R 0.89 (441 [M + H]^+^).

#### 2-(*N*-(1-(2-((((Benzyloxy)carbonyl)amino)methyl)phenyl)ethyl)pivalamido)acetic Acid (**28E**)

An intermediate of **28E** (62 mg, 0.14 mmol) was dissolved in methanol (1 mL), and 2.5 M sodium hydroxide (168 μL, 0.42 mmol) was added and the mixture was stirred at rt over the weekend. The volatiles were removed, and the residue was dissolved in water. The pH was adjusted carefully to 4 by the addition of 2 M HCl, and the mixture was extracted with ethyl acetate. The aqueous pH was again adjusted to 4, and the mixture was extracted with ethyl acetate. The organics were washed with brine, dried over magnesium sulfate, filtered, and the filtrate was evaporated to provide **28E** (68 mg, quantitative yield) that was used directly in the next step. UPLC-MS (short basic) ^*t*^R 0.51 (427 [M + H]^+^).

#### Benzyl 2-(1-(*N*-(2-Oxo-2-((2′-oxo-1,1′,2′,3-tetrahydrospiro[indene-2,3′-pyrrolo[2,3-*b*]pyridin]-5-yl)amino)ethyl)pivalamido)ethyl)benzylcarbamate

**28E** (60 mg, 0.14 mmol) was dissolved in DMF (1.5 mL) under an argon atmosphere, and *N*,*N*-diisopropylethylamine (73 μL, 0.42 mmol) was added. EDCI·HCl (33 mg, 0.17 mmol) and HOAt (23 mg, 0.17 mmol) were added followed by **28F** (38 mg, 0.15 mmol). The mixture was stirred at rt overnight. The reaction mixture was poured into saturated sodium bicarbonate and extracted three times with ethyl acetate and brine, dried over magnesium sulfate, filtered, and the filtrate was evaporated. The residue was purified by column chromatography (4:1 heptane/acetone) to provide benzyl 2-(1-(*N*-(2-oxo-2-((2′-oxo-1,1′,2′,3-tetrahydrospiro[indene-2,3′-pyrrolo[2,3-*b*]pyridin]-5-yl)amino)ethyl)pivalamido)ethyl)benzylcarbamate (90 mg, 97%). UPLC-MS (short basic) ^*t*^R 0.80 (658 [M + H]^+^).

#### *N*-(1-(2-(Aminomethyl)phenyl)ethyl)-*N*-(2-oxo-2-((2′-oxo-1,1′,2′,3-tetrahydrospiro[indene-2,3′-pyrrolo[2,3-*b*]pyridin]-5-yl)amino)ethyl)pivalamide

**Scheme 6:**
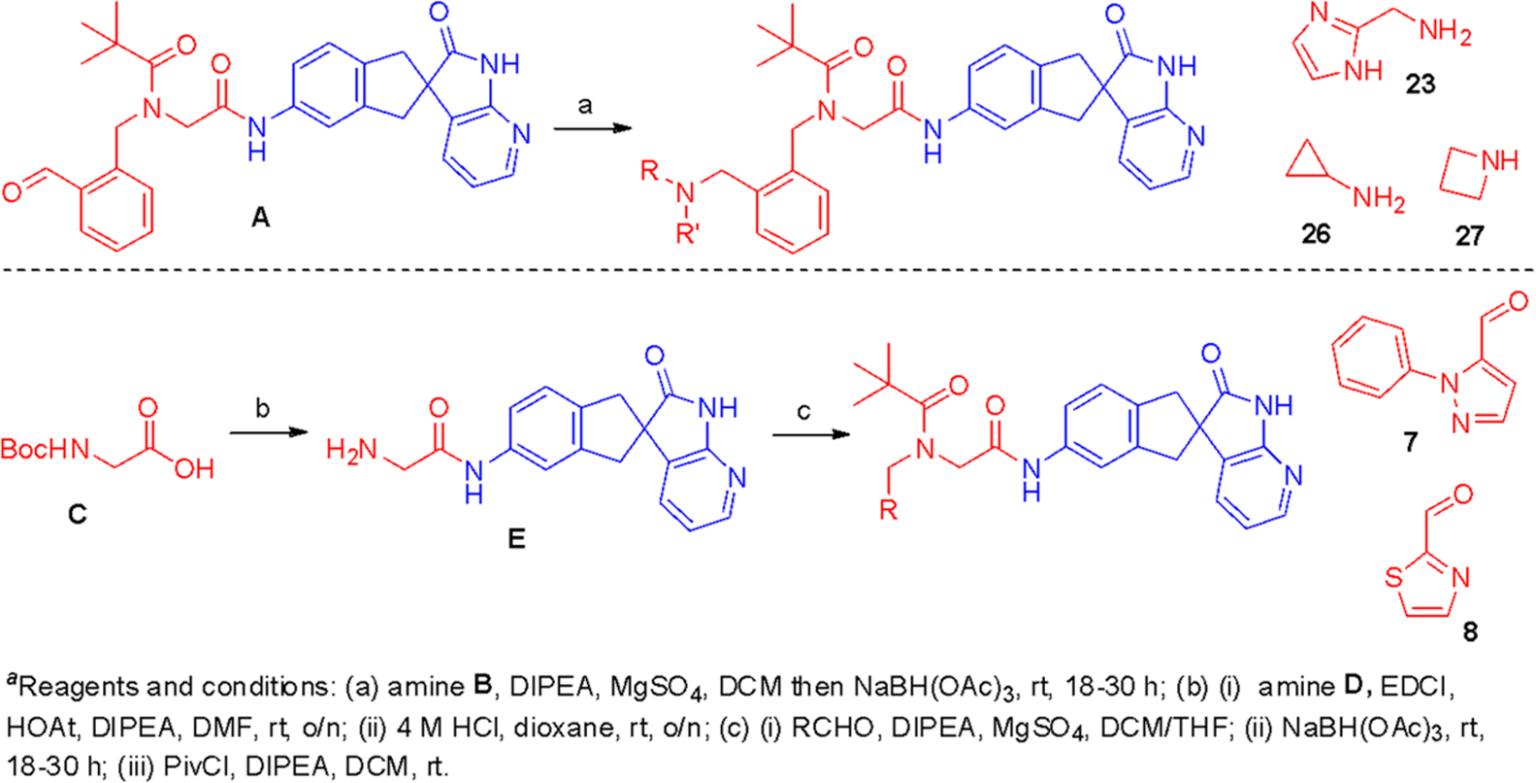
Synthesis of **7**, **8**, **23**, **26**, and **27**

To a solution of benzyl 2-(1-(*N*-(2-oxo-2-((2′-oxo-1,1′,2′,3-tetrahydrospiro[indene-2,3′-pyrrolo[2,3-*b*]pyridin]-5-yl)amino)ethyl)pivalamido)ethyl)benzylcarbamate (90 mg, 0.14 mmol) in ethanol (2 mL) was added 10% Pd/C (9 mg) under a nitrogen atmosphere. The suspension was degassed under vacuum and purged with hydrogen three times. The resulting mixture was stirred under 700 psi of hydrogen pressure at 50 °C for 9 h. The mixture was filtered through celite, and the filter liquid was concentrated and purified by preparative HPLC (acetonitrile/NH_4_COOH buffer over 8 min) to provide **28** (33 mg, 45% yield). ^1^H NMR (CDCl_3_, 300 MHz) δ 1.28 (s, 9H), 1.49 (d, *J* = 6.7 Hz, 3H), 3.03 (s, 2H), 3.47 (dd, *J* = 15.5, 4.0 Hz, 2H), 3.82 (d, *J* = 14.1 Hz, 1H), 3.99 (d, *J* = 14.1 Hz, 1H), 4.28 (s, 1H), 6.10 (s, 1H), 6.83–6.91 (m, 1H), 7.03–7.48 (m, 8H), 8.02–8.05 (m, 1H). UPLC-MS (short basic) ^*t*^R 1.80 (526 [M + H]^+^)(Scheme 6).

#### *N*-(2-((((1*H*-Imidazol-2-yl)methyl)amino)methyl)benzyl)-*N*-(2-oxo-2-((2′-oxo-1,1′,2′,3-tetrahydrospiro[indene-2,3′-pyrrolo[2,3-*b*]pyridine]-5-yl)amino)ethyl)pivalamide (**23**)

**23A** (30 mg, 0.059 mmol) was dissolved in dichloromethane (3 mL), and **23B** (12 mg, 0.076 mmol) was added. *N*,*N*-Diisopropylethylamine (0.028 mL, 0.15 mmol) and magnesium sulfate were added, and the mixture was stirred at rt. After 20 h, sodium triacetoxyborohydride (20 mg, 0.094 mmol) was added, and the reaction was stirred at room temperature, monitoring by UPLC-MS. Extra sodium triacetoxyborohydride (20 mg, 0.094 mmol) was added as required. Once complete, the reaction was poured into saturated sodium bicarbonate and the mixture was extracted three times with dichloromethane. The combined organics were dried over sodium sulfate, filtered, and evaporated. The crude was purified via SPE (2 g STMAd MeOH and then NH_3_ in MeOH, followed by 2 g SiO_2_ 0–10% MeOH in EtOAc and then 10% MeOH in DCM) to provide **23** (13 mg, 37%) as a pale yellow solid. ^1^H NMR (CD_3_OD, 400 MHz) δ 1.29 (s, 9H), 3.04 (dd, *J* = 15.9, 4.1 Hz, 2H), 3.48 (dd, *J* = 15.9, 9.2 Hz, 2H), 3.63 (br d, 1H), 3.70 (br d, 1H), 3.78 (s, 2H), 3.90 (s, 2H), 4.97 (br s, 2H), 6.84 (dd, *J* = 7.4, 5.4 Hz, 1H), 6.98 (s, 2H), 7.09 (d, 1H), 7.20 (dd, *J* = 7.4, 1.5 Hz, 1H), 7.17–7.37 (m, 5H), 7.52 (s, 1H), 8.03 (d, *J* = 5.3, 1.5 Hz, 1H). UPLC-MS ^*t*^R 0.64 (592 [M + H]^+^), 92% pure.

#### *N*-(2-((Cyclopropylamino)methyl)benzyl)-*N*-(2-oxo-2-((2′-oxo-1,1′,2′,3-tetrahydrospiro[indene-2,3′-pyrrolo[2,3-*b*]pyridin]-5-yl)amino)ethyl)pivalamide (**26**)

**26A** (30 mg, 0.059 mmol) was dissolved in dichloromethane (3 mL), and **26B** (8.3 μL, 0.12 mmol) was added. *N*,*N*-Diisopropylethylamine (0.028 mL, 0.15 mmol) and magnesium sulfate were added, and the mixture was stirred at rt. After 6 h, sodium triacetoxyborohydride (20 mg, 0.094 mmol) was added and the reaction was stirred at rt for 48 h. The reaction was poured into saturated sodium bicarbonate and extracted three times with dichloromethane. The combined organics were dried over sodium sulfate, filtered, and evaporated. The crude was purified via SPE (2 g SiO_2_ 0–12% MeOH in EtOAc) and trituration in diethyl ether to provide **26** (9 mg, 28%) as a colorless solid. ^1^H NMR (CDCl_3_, 300 MHz) δ 0.27–0.33 (m, 2H), 0.40–0.46 (m, 2H), 1.30 (s, 9H), 2.14–2.22 (m, 1H), 3.05 (dd, *J* = 15.9, 7.1 Hz, 2H), 3.61 (dd, *J* = 16.0, 6.8 Hz, 2H), 3.85 (s, 2H), 4.08 (br s, 2H), 5.05 (br s, 2H), 6.82 (dd, *J* = 7.3, 5.3 Hz, 1H), 7.05–7.13 (m, 2H), 7.27–7.33 (m, 5H), 7.55 (s, 1H), 8.11 (d, *J* = 5.3, 1.6 Hz, 1H), 8.20 (br s, 1H), 8.58 (br s, 1H). UPLC-MS ^*t*^R 0.78 (552 [M + H]^+^), 95% pure.

#### *N*-(2-(Azetidin-1-ylmethyl)benzyl)-*N*-(2-oxo-2-((2′-oxo-1,1′,2′,3-tetrahydrospiro[indene-2,3′-pyrrolo[2,3-*b*]pyridine]-5-yl)amino)ethyl)pivalamide (**27**)

**27A** (30 mg, 0.059 mmol) was dissolved in dichloromethane (3 mL), and **27B** (11 mg, 0.12 mmol) was added. *N*,*N*-Diisopropylethylamine (0.028 mL, 0.15 mmol) and magnesium sulfate were added, and the mixture was stirred at rt. After 6 h, sodium triacetoxyborohydride (20 mg, 0.094 mmol) was added and the reaction was stirred at rt for 30 h. The reaction was poured into saturated sodium bicarbonate and extracted three times with dichloromethane. The combined organics were dried over sodium sulfate, filtered, and evaporated. The crude was purified via SPE (2 g SiO_2_ 0–10% MeOH in EtOAc and then 10% MeOH in DCM) to provide **27** (21 mg, 65%) as a colorless glass. ^1^H NMR (CDCl_3_, 300 MHz) δ 1.33 (s, 9H), 1.97–2.10 (m, 2H), 3.02 (dd, *J* = 15.8, 3.5 Hz, 2H), 3.08–3.20 (m, 4H), 3.52–3.66 (m, 4H), 4.07–4.17 (m, 2H), 5.11 (br s, 2H), 6.81 (dd, *J* = 7.3, 5.3 Hz, 1H), 7.02–7.30 (m, 7H), 7.53 (br s, 1H), 8.13 (d, *J* = 5.3, 1.4 Hz, 1H), 8.68 (br d, 2H). UPLC-MS ^*t*^R 0.78 (552 [M + H]^+^), 95% pure.

#### *tert*-Butyl (2-Oxo-2-((2′-oxo-1,1′,2′,3-tetrahydrospiro[indene-2,3′-pyrrolo[2,3-*b*]pyridine]-5-yl)amino)ethyl)carbamate (**7E**/**8E**)

*N*,*N*-Diisopropylethylamine (6.24 mL, 35.8 mmol) was added to a solution of Boc-glycine-OH (2.4 g, 13.7 mmol), EDCI.HCl (2.52 g, 13.2 mmol), and HOAt (1.8 g, 13.2 mmol) in DMF (25 mL) under an argon atmosphere. Amine **D** (3.0 g, 11.9 mmol) was added, washing in with DMF (10 mL). The mixture was stirred at rt for 18 h, after which time the reaction was complete as assessed by UPLC-MS. The mixture was poured into saturated sodium bicarbonate. The aqueous layer was extracted three times with ethyl acetate. The combined organic extracts were washed three times with water, 20% aqueous citric acid, three more times with water, dried over magnesium sulfate, filtered, and the filtrate was evaporated to provide the intermediate of **7E**/**8E** (4.84 mg, 99%) as a pale yellow glass. ^1^H NMR (CD_3_OD, 300 MHz) δ 1.45 (s, 9H), 3.06 (dd, *J* = 15.7, 6.1 Hz, 2H), 3.50 (dd, *J* = 16.0, 8.2 Hz, 2H), 3.84 (br s, 2H), 6.87 (dd, *J* = 7.3, 5.3 Hz, 1H), 7.13 (d, *J* = 7.9 Hz, 1H), 7.22 (d, *J* = 7.5 Hz, 1H), 7.38 (d, *J* = 7.8 Hz, 1H), 7.55 (s, 1H), 8.03 (d, *J* = 3.8 Hz, 1H). UPLC-MS (CSH 2–50%) ^*t*^R 0.93 (409 [M + H]^+^).

#### 2-Amino-*N*-(2′-oxo-1,1′,2′,3-tetrahydrospiro[indene-2,3′-pyrrolo[2,3-*b*]pyridine]-5-yl)acetamide Dihydrochloride (**7E**/**8E**)

An intermediate of **7E**/**8E** (4.84 mL, 11.9 mmol) was triturated in 3 M HCl in cyclopentyl methyl ether (20 mL, 60 mmol) until a flowing suspension was obtained. The mixture was stirred at rt for 3 h after which the reaction was judged complete by UPLC-MS. The solid was isolated by decanting the solvent and then washed and decanted three times with diethyl ether. The solid was dried to provide **7E**/**8E** (4.62 mg, quantitative yield) as a beige powder. UPLC-MS (short basic) ^*t*^R 0.43 (309 [M + H]^+^), 93% pure.

#### *N*-(2-Oxo-2-((2′-oxo-1,1′,2′,3-tetrahydrospiro[indene-2,3′-pyrrolo[2,3-*b*]pyridin]-5-yl)amino)ethyl)-*N*-((1-phenyl-1*H*-pyrazol-5-yl)methyl)pivalamide (**7**)

**7E** (52 mg, 0.14 mmol) was dissolved in dichloromethane (1.4 mL) and tetrahydrofuran (1.4 mL) and then *N*,*N*-diisopropylethylamine (0.065 mL, 0.37 mmol). 1-Phenyl-1*H*-pyrazole-5-carbaldehyde (27 mg, 0.16 mmol), sodium triacetoxyborohydride (100 mg, 0.47 mmol), and magnesium sulfate were added, and the mixture was stirred at rt overnight. The reaction mixture was filtered, poured into water, and the pH was adjusted to 4 with 2 M HCl and washed twice with dichloromethane. The aqueous layer was basified with sodium carbonate and extracted twice with dichloromethane. This organic extract was dried over magnesium sulfate, filtered, and evaporated to provide the intermediate of **7**. This compound was dissolved in dichloromethane (2 mL) under an argon atmosphere, and *N*,*N*-diisopropylethylamine (70 μL, 0.41 mmol) was added and the mixture was stirred at 5 °C (ice/water). Trimethylacetyl chloride (258 μL, 0.21 mmol) was added dropwise, and the mixture was stirred at rt overnight. The reaction mixture was diluted in dichloromethane, washed with brine and saturated sodium bicarbonate, dried over magnesium sulfate, filtered, and the filtrate was evaporated. The residue was purified via MDAP (XBridge C18 19 × 150, 30–60% acetonitrile water with 0.1% ammonium hydroxide) to provide **7** (11 mg, 14%). ^1^H NMR (CD_3_OD, 300 MHz) δ 1.21 (s, 9H), 3.05 (dd, *J* = 15.9, 2.0 Hz, 2H), 3.48 (dd, *J* = 15.8, 5.8 Hz, 2H), 4.15 (s, 2H), 4.82 (s, 2H), 6.40 (s, br, 1H), 6.87 (dd, *J* = 7.1, 5.1 Hz, 1H), 7.12 (dd, *J* = 7.3, 1.5 Hz, 1H), 7.20 (d, *J* = 8.1 Hz, 1H), 7.29 (dd, *J* = 8.1, 1.7 Hz, 1H), 7.41–7.54 (m, 6H), 7.66 (s, 1H), 8.03 (dd, *J* = 5.4, 1.5 Hz, 1H). UPLC-MS (short basic) ^*t*^R 1.99 (549 [M + H]^+^).

#### *N*-(2-Oxo-2-((2′-oxo-1,1′,2′,3-tetrahydrospiro[indene-2,3′-pyrrolo[2,3-*b*]pyridin]-5-yl)amino)ethyl)-*N*-(thiazol-2-ylmethyl)pivalamide (**8**)

**8E** (52 mg, 0.14 mmol) was dissolved in dichloromethane (1.4 mL) and tetrahydrofuran (1.4 mL) and *N*,*N*-diisopropylethylamine (0.065 mL, 0.37 mmol). Thiazole-2-carbaldehyde (15 mg, 0.14 mmol), sodium triacetoxyborohydride (100 mg, 0.47 mmol), and magnesium sulfate were added, and the mixture was stirred at rt overnight. The reaction mixture was filtered, poured into water, and the pH was adjusted to 4 with 2 M HCl before being washed twice with dichloromethane. The aqueous layer was basified with sodium carbonate and extracted twice with dichloromethane. This organic extract was dried over magnesium sulfate, filtered, and evaporated to provide an intermediate of **8**. This compound was dissolved in dichloromethane (2.5 mL) under an argon atmosphere, and *N*,*N*-diisopropylethylamine (70 μL, 0.41 mmol) was added and the mixture was stirred at 5 °C (ice/water). Trimethylacetyl chloride (221 μL, 0.18 mmol) was added dropwise, and the mixture was stirred at rt overnight. The reaction mixture was diluted in dichloromethane, washed with brine and saturated sodium bicarbonate, dried over magnesium sulfate, filtered, and the filtrate was evaporated. The residue was purified via MDAP (XBridge C18 19 × 150, 30–60% acetonitrile water with 0.1% ammonium hydroxide) to provide **8** (14 mg, 20%). ^1^H NMR (CD_3_OD, 300 MHz) δ 1.29 (s, 9H), 3.10 (dd, *J* = 15.8, 6.5 Hz, 2H), 3.51 (dd, *J* = 15.9, 8.2 Hz, 2H), 4.31 (s, 2H), 5.06 (s, 2H), 6.87 (dd, *J* = 7.4, 5.5 Hz, 1H), 7.12 (dd, *J* = 7.4, 1.9 Hz, 1H), 7.20–7.25 (m, 1H), 7.35–7.40 (m, 1H), 7.55–7.59 (m, 2H), 7.72–7.75 (m, 1H), 8.03 (dd, *J* = 5.5, 1.9 Hz, 1H). UPLC-MS (short basic) ^*t*^R 1.80 (490 [M + H]^+^).

### Synthesis of **30** (See Scheme 2)

#### (*R*)-1′-(*tert*-Butyl)-5-(dibenzylamino)-1,3-dihydrospiro[indene-2,3′-pyrrolo[2,3-*b*]pyridin]-2′(1*′H*)-one (**33**)

To a solution of sodium hydroxide (72 g) in water (60 mL) at room temperature were added toluene (130 mL) and [2-(chloromethyl)-4-(dibenzylamino)phenyl]methanol hydrochloride (4.7 g, 12.1 mmol). The reaction mixture was stirred at room temperature, while bubbling with argon, for 5 min. Methyl 1-*tert*-butyl-2-hydroxy-1*H*-pyrrolo[2,3-*b*]pyridine-3-carboxylate (3.0 g, 12.1 mmol) was added in three portions over 10 min. Argon continued to be bubbled through the stirring solution for 15 min, and (9*R*)-1-[3,5-bis(trifluoromethyl)benzyl]cinchonan-1-ium-9-ol bromide (0.7 g, 1.2 mmol) was added in one portion at room temperature. This mixture was stirred at room temperature for 3 h under bubbling argon. Water (∼300 mL) was added [note: exothermic reaction], and the mixture was stirred for ∼15 min while warming to room temperature. The two layers were separated, and the aqueous layer was extracted by ethyl acetate. The combined extracts were washed with water, dried over magnesium sulfate, filtered, and evaporated to give the crude product of ∼90% purity, 83% *ee*. This product was dissolved in toluene (60 mL) at 60 °C. Once totally dissolved, the mixture was warmed to room temperature and methanol (180 mL) was added. The mixture was stirred at room temperature for 16 h, and the resulting crystals were collected by filtration and washed with methanol to give **33** (61%, 96% *ee*). The product was further recrystallized using toluene (50 mL) and methanol (120 mL) to give 3.1 g (52%, >99% *ee*) of the product. ^1^H NMR (CD_3_OD, 400 MHz) δ 1.82 (s, 9H), 2.88 (dd, *J* = 15.2, 11.8 Hz, 2H), 3.48 (t, *J* = 15.3 Hz, 2H), 4.67 (s, br, 4H), 6.67 (s, br, 1H), 6.78 (dd, *J* = 7.1, 5.3 Hz, 1H), 7.01–7.14 (m, 2H), 7.25–7.40 (m, 11H), 8.15 (dd, *J* = 5.2, 1.7 Hz, 1H); LC-MS (488.27 [M + H]^+^). Chiral HPLC: Phenomenex Lux 3μ Cellulose-1 column; ^*n*^hexane/isopropanol, 95:5; flow rate = 1.0 mL/min; detection at 254 nm.

#### (*R*)-5-Amino-1,3-dihydrospiro[indene-2,3′-pyrrolo[2,3-*b*]pyridin]-2′(1*′H*)-one (**30**)

To a solution of **33** (3.1 g, 6.36 mmol) in methanol (120 mL) was added methanesulfonic acid (11 mL) at room temperature. The mixture was stirred at reflux for 4 h. The methanol was removed under vacuum, and water (∼100 mL) was added to the mixture, and pH was adjusted to ∼ 10 by adding a 50% aqueous solution of sodium hydroxide. The aqueous layer was extracted with ethyl acetate, and the combined extracts were dried over magnesium sulfate, filtered, and evaporated to give the crude product. The crude product was dissolved in methanol (∼80 mL), and Pd/C (0.1 g) was added to the solution followed by concentrated HCl (7 mL). The mixture was stirred at room temperature under a balloon of H_2_ overnight. Volatiles were removed to dryness, and the crude material was then dissolved in dichloromethane. Water followed by saturated aqueous potassium carbonate was added to pH ∼ 10. The mixture was extracted by dichloromethane, dried over magnesium sulfate, filtered, and evaporated to give 1.2 g (77%, >99% *ee*) of the desired product **30**. This compound was used directly in the next step without further purification. ^1^H NMR (CD_3_OD, 400 MHz) δ 2.94 (dd, *J* = 15.3, 4.4 Hz, 2H), 3.46 (t, *J* = 14.0 Hz, 2H), 6.65 (d, *J* = 8.1 Hz, 1H), 6.69 (s, br, 1H), 6.88 (dd, *J* = 8.9, 3.8 Hz, 1H), 7.02 (d, *J* = 8.1 Hz, 1H), 7.13 (d, *J* = 7.3 Hz, 1H), 8.02–8.06 (m, 1H); LC-MS (252.11 [M + H]^+^); [α]_D_^22^ = +63.6 (*c* 1.1, MeOH). Chiral HPLC**:** Phenomenex Lux 3μ Cellulose-1 column; ^*n*^hexane/isopropanol, 40:60; flow rate = 0.5 mL/min; detection at 220 nm.

### Synthesis of (*R*)-**25**

This compound was synthesized according to the experimental procedure described for **25** using **30** instead of D (see Scheme 4).^34^ Analytical data for (*R*)**-25**: ^1^H NMR (CD_3_OD, 300 MHz) δ 1.32 (s, 9H), 2.41 (s, 3H), 3.04 (dd, *J* = 15.9, 3.9 Hz, 2H), 3.50 (dd, *J* = 15.8, 7.4 Hz, 2H), 3.72 (s, 2H), 4.11 (br s, 2H), 4.96 (br s, 2H), 6.86 (dd, *J* = 7.4, 5.4 Hz, 1H), 7.08–7.14 (m, 1H), 7.17–7.23 (m, 2H), 7.26–7.37 (m, 4H), 7.52 (s, 1H), 8.03 (d, *J* = 5.4, 1.6 Hz, 1H); [α]_D_^22^ = +40.8 (*c* 1.0, MeOH).

### Cell Lines and Culture Conditions

All cell lines were purchased from ATCC, Cell Applications, Inc., or DiscoverX with proof of authentication, unless stated. All cell lines were maintained at 37 °C in a humidified atmosphere with 5% CO_2_. Human breast cancer cells MDA-MB-231 (ATCC, HTB-26) were cultured in RPMI 1640 medium, GlutaMAX supplement (Thermo Fisher Scientific, 61870-036) containing 10% (v/v) fetal bovine serum (FBS, Thermo Fisher Scientific, 10500-064), and 1% (v/v) penicillin–streptomycin (Sigma-Aldrich, P4333). CGRP (95-0164C6) receptor- and AM_2_ (95-0169C6) receptor-overexpressing cell lines were obtained from DiscoverX and cultured in AssayComplete Cell Culture Kit 105 (92-3105G) supplemented with 800 μg/mL G418 and 2.5 μg/mL puromycin. The receptor component expression of these cells was validated in-house previously.^34^

### Time-Resolved Fluorescence Resonance Energy-Transfer (TR-FRET) cAMP Accumulation

The ability of the compounds to inhibit cAMP production induced by an EC_50_ concentration of the maximum agonist activation (information previously published^34^) in GPCR/RAMP-overexpressing cells (i.e., AM_2_, CGRP cells) was evaluated using cAMP accumulation assays. Each compound was tested at 8 full-log concentrations (10^–11^–10^–5^ M) including a negative control (blank). The total cAMP was measured using the TR-FRET LANCE cAMP detection kit (PerkinElmer, AD0264), as described previously.^34^ Briefly, frozen cells (2 × 10^6^ in each well) were thawed and prepared in warm stimulation buffer (1× HBSS, 5 mM *N*-(2-hydroxyethyl)piperazine-*N*′-ethanesulfonic acid (HEPES), 0.5 mM IBMX, and 0.1% bovine serum albumin (BSA)). Alexa Fluor conjugated anti-cAMP (1:100 concentration) was then added to the cell suspension, and cells were plated (2500 cells, 6 μL) in a 384-well white opaque microtiter plate (OptiPlates, PerkinElmer, 6007299). Cells were first preincubated with serial dilutions (3 μL) of the antagonists for 30 min at room temperature prior to their stimulation with the EC_50_ value of agonist (3 μL) for 15 min at room temperature. Subsequently, a 12 μL detection mix (Europium-Chelate streptavidin/biotinylated cAMP) was added to stop the reaction and induce cell lysis. TR-FRET was detected after an hour of incubation by an EnSight multimode Plate reader (PerkinElmer) at 320/340 nm excitation and 615/665 nm emission. Data were normalized to agonist only and blank (stimulation buffer only) wells as 0 and 100% cAMP inhibition, respectively.

The final DMSO concentration was below 0.5%, and this was kept consistent in all of the wells, including agonist alone and blank. The same methodology including the number of cells was used for all cell lines. Concentration–response curves were analyzed using three-parameter logistic curve fitting to determine IC_50_ values (Graphpad Prism 7 and 8). No further constraints in any parameters of the curves were used.

### Real-Time-Glo MT Viability Assay

Cell viability in human breast cancer cells (MDA-MB-231) was quantified using Real-Time-Glo MT Cell Viability Assay (Promega, G9712) as previously described.^34^ Cells (2000 cells) were seeded into 96-well white clear-bottom plates (Corning, 3903) in full serum media overnight before washing and changing to suboptimal media (RPMI + 5% FBS + 1% P/S) containing Real-Time-Glo reagents according to the Promega protocol. A baseline luminescence read (prior to treatment) was taken using an EnSight Multimode Plate Reader (PerkinElmer) after an hour of incubation at 37 °C. Cells were then treated with compounds or vehicle control (PBS + 0.05% DMSO) daily. Results were normalized to vehicle-treated controls as 100% viable (Graphpad Prism 7 and 8).

### Ethical Statement for *In Vivo* Studies

The *in vivo* study plans were assessed by a local research ethics committee before submission for Home Office approval. All *in vivo* experiments were carried out under the authority of project and personal licenses granted by the U.K. Home Office under the U.K. Animals (Scientific Procedures) Act 1986 (ASPA).

### *In Vivo* Efficacy Model

The study was performed using 6–7 week old BALB/c nude female mice, with a weight range of 15–20 g. Animals were provided by Envigo Corporation (Cambridgeshire, U.K.) or Charles River Laboratories (Massachusetts) depending on availability. Each experiment started with 10 mice (experimental units) in each experimental/control group. Subsequent analysis (tumor growth and histology) was only performed in animals where tumors had established and were palpable within 3 days of implantation. This was in accordance with the power calculation performed to ensure robust statistical analysis by the University of Sheffield Statistical Service. The animals were housed in individually ventilated cages (with the appropriate bedding and flooring conditions) in environmentally controlled conditions with a 12 h light/dark cycles at ∼26 °C. Mice had access to an adequate amount of water and a 2018 Teklad Global 18% Protein Rodent Diet containing 1.01% calcium (Harlan Laboratories, U.K.). The day-to-day care of the animals was carried out by the technicians in the Biological Services (The University of Sheffield, U.K.). All scientific procedures on animals were carried under the U.K. Home Office Project Licenses (40/3499) and Procedure Individual Licenses.

### Compound Preparation for *In Vivo* Studies

Compounds were dissolved in DMSO (Sigma-Aldrich, D4540) and sonicated at 37 °C for 10 min. The appropriate volume of solvent (Kolliphor HS15 (1 part, grams), Kollisolv PEG E 400 (3 parts, mL), and PBS (6 parts, mL)) was then added to yield a 6% DMSO/94% solvent solution. These working stocks (8 mg/mL) were further sonicated at 37 °C for 10 min before storing at −20 °C. To make treatment aliquots, equal amounts of the working stock (or vehicle control) and solvent were mixed and sonicated at 37 °C for 10 min (4 mg/mL, equivalent to 20 mg/kg, 3% DMSO/97% solvent). Vehicle control and compounds were sonicated at 37 °C for 10 min prior to ip injections (200 μL per mouse).

### Cell Preparation and Tumor Inoculation

Cells were prepared according to standard cell culture techniques. Cell pellets were resuspended in 50% PBS/50% Matrigel (Corning, 354234). Matrigel/PBS cell suspension, needles (25G), and syringes (1 mL) were kept on ice before and during tumor inoculation into mice. Cell suspension (100 μL, 5 × 10^6^ cells) was injected subcutaneously into the left flank of 6–7 weeks old female immunodeficient nude athymic mice (BALB/c nude). Once the tumors became palpable (around 100 mm^3^), mice were randomized into treatment groups. Mice were treated daily at the same time of the day with an ip injection of 20 mg/kg of compound or vehicle control (200 μL per mouse) until humane end point. Mice were observed for at least 30 min post treatment to detect any acute adverse effects. Tumor size and mouse weights were measured twice a week. At the end of each study, the animals were euthanized following the appropriate procedures listed in the ASPA Act 1986. Vital organs and tumors were stored in 10% neutral-buffered formalin for further histological analysis. The primary experimental outcome was tumor volume. The percentage tumor volume was calculated by normalizing measured tumor volumes to the initial tumor volume prior to the start of treatment on day 5. Simple linear regression was done to compare the line of best fit between the growth curves. Blinding was not used for the *in vivo* studies.

## Supporting Information Available

AM_2_ hybrid model (PDB)
Telcagepant docking (PDB)
Compound **1** docking (PDB)
Compound (*R*)-**25** docking (PDB)
Molecular formula strings (CSV)
X-ray crystal structure analysis of (*R*)-CLR-binding unit **30** and key compounds **12**, **14**, (±)-**25**, and (*R*)-**25** chemical data (^1^H NMR, HPLC, LC-MS) (PDF)

## Supplementary Material

jm0c02191_si_001.pdb

jm0c02191_si_002.pdb

jm0c02191_si_003.pdb

jm0c02191_si_004.pdb

## Notes

All data generated or analyzed during this study are either included in this published article (and its Supporting Information) or are available from the corresponding authors on reasonable request.

## Abbreviations

AM: adrenomedullin
AM_1_: adrenomedullin-1
AM_2_: adrenomedullin-2
CLR: calcitonin receptor-like receptor
CSH: charged surface hybrid
CTR: calcitonin receptor
DIPEA: *N*,*N*-diisopropylethylamine
EDCI.HCl: 1-ethyl-3-(3-dimethylaminopropyl)carbodiimide hydrochloride salt
HATU: hexafluorophosphate azabenzotriazole tetramethyl uranium
HBSS: Hanks balanced salt solution
HEPES: 4-(2-hydroxyethyl)-1-piperazineethanesulfonic acid
HOAt: 1-hydroxy-7-azabenzotriazole
IBMX: 3-isobutyl-1-methylxanthine
LC-MS: liquid chromatography-mass spectrometry
MDAP: mass directed autopurification
MW: microwave
NMM: *N*-methylmorpholine
PDA: photodiode array
*p*TSA: *p*-toluene sulfonic acid
QDA: quadrupole dalton
RAMP: receptor activity-modifying proteins
^*t*^R: retention time
rt: room temperature
SCX2: strong cation-exchange 2 (SPE from Biotage)
SEM: trimethylsilylethoxymethyl
SPE: solid-phase extraction
UPLC: ultraperformance liquid chromatography

## References

[ref1] Alexander S. P. H.; Christopoulos A.; Davenport A. P.; Kelly E.; Mathie A.; Peters J. A.; Veale E. L.; Armstrong J. F.; Faccenda E.; Harding S. D.; Pawson A. J.; Sharman J. L.; Southan C.; Davies J. A.; Arumugam T. V.; Bennett A.; Sjogren B.; Sobey C.; Wong S. S.; Abbracchio M. P.; Alexander W.; Al-hosaini K.; Back M.; Beaulieu J. M.; Bernstein K. E.; Bettler B.; Birdsall N. J. M.; Blaho V.; Bousquet C.; Brauner-Osborne H.; Burnstock G.; Calo G.; Castano J. P.; Catt K. J.; Ceruti S.; Chazot P.; Chiang N.; Chun J.; Cianciulli A.; Clapp L. H.; Couture R.; Csaba Z.; Dent G.; Singh K. D.; Douglas S. D.; Dournaud P.; Eguchi S.; Escher E.; Filardo E.; Fong T. M.; Fumagalli M.; Gainetdinov R. R.; de Gasparo M.; Gershengorn M.; Gobeil F.; Goodfriend T. L.; Goudet C.; Gregory K. J.; Gundlach A. L.; Hamann J.; Hanson J.; Hauger R. L.; Hay D.; Heinemann A.; Hollenberg M. D.; Holliday N. D.; Horiuchi M.; Hoyer D.; Hunyady L.; Husain A.; Ijzerman A. P.; Inagami T.; Jacobson K. A.; Jensen R. T.; Jockers R.; Jonnalagadda D.; Karnik S.; Kaupmann K.; Kemp J.; Kennedy C.; Kihara Y.; Kozielewicz P.; Kreienkamp H. J.; Kukkonen J. P.; Langenhan T.; Leach K.; Lecca D.; Lee J. D.; Leeman S. E.; Leprince J.; Lolait S. J.; Lupp A.; Macrae R.; Maguire J.; Mazella J.; McArdle C. A.; Melmed S.; Michel M. C.; Miller L.; Mitolo V.; Mouillac B.; Murphy P. M.; Nahon J. L.; Norel X.; Nyimanu D.; O’Carroll A. M.; Offermanns S.; Panaro M. A.; Pertwee R. G.; Pin J. P.; Prossnitz E.; Ramachandran R.; Reinscheid R. K.; Rondard P.; Rovati G. E.; Ruzza C.; Sanger G.; Schoneberg T.; Schulte G.; Schulz S.; Segaloff D. L.; Serhan C. N.; Stoddart L. A.; Sugimoto Y.; Summers R.; Tan V.; Thomas W.; Timmermans P.; Tirupula K.; Tulipano G.; Unal H.; Unger T.; Vanderheyden P.; Vaudry D.; Vaudry H.; Vilardaga J. P.; Walker C. S.; Ward D. T.; Wester H. J.; Willars G. B.; Williams T. L.; Woodruff T. M.; Yao C. C.; Aldrich R. W.; Becirovic E.; Biel M.; Catterall W. A.; Conner A. C.; Davies P.; Delling M.; Di Virgilio F.; Falzoni S.; George C.; Goldstein S. A. N.; Grissmer S.; Ha K.; Hammelmann V.; Hanukoglu I.; Jarvis M.; Jensen A. A.; Kaczmarek L. K.; Kellenberger S.; King B.; Lynch J. W.; Perez-Reyes E.; Plant L. D.; Rash L. D.; Ren D. J.; Sivilotti L. G.; Smart T. G.; Snutch T. P.; Tian J. B.; Van den Eynde C.; Vriens J.; Wei A. D.; Winn B. T.; Wulff H.; Xu H. X.; Yue L. X.; Zhang X. L.; Zhu M.; Coons L.; Fuller P.; Korach K. S.; Young M.; Bryant C.; Farndale R. W.; Hobbs A.; Jarvis G. E.; MacEwan D.; Monie T. P.; Waldman S.; Beuve A.; Boison D.; Brouckaert P.; Burnett J. C.; Burns K.; Dessauer C.; Friebe A.; Garthwaite J.; Gertsch J.; Helsby N.; Izzo A. A.; Koesling D.; Kuhn M.; Ostrom R.; Papapetropoulos A.; Potter L. R.; Pyne N. J.; Pyne S.; Russwurm M.; Schmidt H.; Seifert R.; Stasch J. P.; Szabo C.; van der Stelt M.; van der Vliet A.; Watts V.; Anderson C. M. H.; Broer S.; Dawson P.; Hagenbuch B.; Hammond J. R.; Hancox J.; Inui K.; Kanai Y.; Kemp S.; Thwaites D. T.; Verri T.; The concise guide to pharmacology 2019/20: G protein-coupled receptors. Br. J. Pharmacol. 2019, 176, S21–S141. 10.1111/bph.14748. 31710717 PMC6844580

[ref2] Sriram K.; Insel P. A. G protein-coupled receptors as targets for approved drugs: how many targets and how many drugs?. Mol. Pharmacol. 2018, 93, 251–258. 10.1124/mol.117.111062. 29298813 PMC5820538

[ref3] Weston C.; Winfield I.; Harris M.; Hodgson R.; Shah A.; Dowell S. J.; Mobarec J. C.; Woodlock D. A.; Reynolds C. A.; Poyner D. R.; Watkins H. A.; Ladds G. Receptor activity-modifying protein-directed G protein signaling specificity for the calcitonin gene-related peptide family of receptors. J. Biol. Chem. 2016, 291, 21925–21944. 10.1074/jbc.M116.751362. 27566546 PMC5063977

[ref4] Hay D. L.; Pioszak A. A. Receptor activity-modifying proteins (RAMPs): new insights and roles. Annu. Rev. Pharmacol. Toxicol. 2016, 56, 469–487. 10.1146/annurev-pharmtox-010715-103120. 26514202 PMC5559101

[ref5] Hay D. L.; Garelja M. L.; Poyner D. R.; Walker C. S. Update on the pharmacology of calcitonin/CGRP family of peptides: IUPHAR Review 25. Br. J. Pharmacol. 2018, 175, 3–17. 10.1111/bph.14075. 29059473 PMC5740251

[ref6] Bortolato A.; Dore A. S.; Hollenstein K.; Tehan B. G.; Mason J. S.; Marshall F. H. Structure of class B GPCRs: new horizons for drug discovery. Br. J. Pharmacol. 2014, 171, 3132–3145. 10.1111/bph.12689. 24628305 PMC4080969

[ref7] de Graaf C.; Song G. J.; Cao C.; Zhao Q.; Wang M. W.; Wu B. L.; Stevens R. C. Extending the structural view of class B GPCRs. Trends Biochem. Sci. 2017, 42, 946–960. 10.1016/j.tibs.2017.10.003. 29132948

[ref8] Liang Y.-L.; Khoshouei M.; Deganutti G.; Glukhova A.; Koole C.; Peat T. S.; Radjainia M.; Plitzko J. M.; Baumeister W.; Miller L. J.; Hay D. L.; Christopoulos A.; Reynolds C. A.; Wootten D.; Sexton P. M. Cryo-EM structure of the active, G(s)- protein complexed, human CGRP receptor. Nature 2018, 561, 492–497. 10.1038/s41586-018-0535-y. 30209400 PMC6166790

[ref9] Liang Y.-L.; Belousoff M. J.; Fletcher M. M.; Zhang X.; Khoshouei M.; Deganutti G.; Koole C.; Furness S. G. B.; Miller L. J.; Hay D. L.; Christopoulos A.; Reynolds C. A.; Danev R.; Wootten D.; Sexton P. M. Structure and dynamics of adrenomedullin receptors AM1 and AM2 reveal key mechanisms in the control of receptor phenotype by receptor activity-modifying proteins. ACS Pharmacol. Transl. Sci. 2020, 3, 263–284. 10.1021/acsptsci.9b00080. 32296767 PMC7155201

[ref10] Garelja M. L.; Au M.; Brimble M. A.; Gingell J. J.; Hendrikse E. R.; Lovell A.; Prodan N.; Sexton P. M.; Siow A.; Walker C. S.; Watkins H. A.; Williams G. M.; Wootten D.; Yang S. H.; Harris P. W. R.; Hay D. L. Molecular mechanisms of class B GPCR activation: insights from adrenomedullin receptors. ACS Pharmacol. Transl. Sci. 2020, 3, 246–262. 10.1021/acsptsci.9b00083. 32296766 PMC7155197

[ref11] Hoare S. R. J.; Grigoriadis D. E. Non-peptide translation to CRF-receptor antagonists:allosterism, kinetics’ and efficacy in human disease. Curr. Mol. Pharmacol. 2017, 10, 282–295. 10.2174/1874467210666170110124539. 28103785

[ref12] Thal D. M.; Glukhova A.; Sexton P. M.; Christopoulos A. Structural insights into G-protein-coupled receptor allostery. Nature 2018, 559, 45–53. 10.1038/s41586-018-0259-z. 29973731

[ref13] Hendrikse E. R.; Liew L. P.; Bower R. L.; Bonnet M.; Jamaluddin M. A.; Prodan N.; Richards K. D.; Walker C. S.; Pairaudeau G.; Smith D. M.; Rujan R.-M.; Sudra R.; Reynolds C. A.; Booe J. M.; Pioszak A. A.; Flanagan J. U.; Hay M. P.; Hay D. L. Identification of small-molecule positive modulators of calcitonin-like receptor-based receptors. ACS Pharmacol. Transl. Sci. 2020, 3, 305–320. 10.1021/acsptsci.9b00108. 32296770 PMC7155196

[ref14] Hewitt D. J.; Aurora S. K.; Dodick D. W.; Goadsby P. J.; Ge Y.; Bachman R.; Taraborelli D.; Fan X. Y.; Assaid C.; Lines C.; Ho T. W. Randomized controlled trial of the CGRP receptor antagonist MK-3207 in the acute treatment of migraine. Cephalalgia 2011, 31, 712–722. 10.1177/0333102411398399. 21383045

[ref15] Ho T. W.; Connor K. M.; Zhang Y.; Pearlman E.; Koppenhaver J.; Fan X. Y.; Lines C.; Edvinsson L.; Goadsby P. J.; Michelson D. Randomized controlled trial of the CGRP receptor antagonist telcagepant for migraine prevention. Neurology 2014, 83, 958–966. 10.1212/WNL.0000000000000771. 25107879

[ref16] Bell I. M. Calcitonin gene-related peptide receptor antagonists: new therapeutic agents for migraine. J. Med. Chem. 2014, 57, 7838–7858. 10.1021/jm500364u. 24960305

[ref17] Karsan N.; Goadsby P. J. Calcitonin gene-related peptide and migraine. Curr. Opin. Neurol. 2015, 28, 250–254. 10.1097/WCO.0000000000000191. 25887765

[ref18] Ho T. W.; Ho A. P.; Ge Y.; Assaid C.; Gottwald R.; MacGregor E. A.; Mannix L. K.; van Oosterhout W. P. J.; Koppenhaver J.; Lines C.; Ferrari M. D.; Michelson D. Randomized controlled trial of the CGRP receptor antagonist telcagepant for prevention of headache in women with perimenstrual migraine. Cephalalgia 2016, 36, 148–161. 10.1177/0333102415584308. 25926620

[ref19] Croop R.; Goadsby P. J.; Stock D. A.; Conway C. M.; Forshaw M.; Stock E. G.; Coric V.; Lipton R. B. Efficacy, safety, and tolerability of rimegepant orally disintegrating tablet for the acute treatment of migraine: a randomised, phase 3, double-blind, placebo-controlled trial. Lancet 2019, 394, 737–745. 10.1016/S0140-6736(19)31606-X. 31311674

[ref20] Scott L. J. Ubrogepant: first approval. Drugs 2020, 80, 323–328. 10.1007/s40265-020-01264-5. 32020557 PMC7062659

[ref21] Ornello R.; Casalena A.; Frattale I.; Gabriele A.; Affaitati G.; Giamberardino M. A.; Assetta M.; Maddestra M.; Marzoli F.; Viola S.; Cerone D.; Marini C.; Pistoia F.; Sacco S. Real-life data on the efficacy and safety of erenumab in the Abruzzo region, central Italy. J. Headache Pain 2020, 21, 32 10.1186/s10194-020-01102-9. 32264820 PMC7137484

[ref22] Lipton R. B.; Goadsby P. J.; Smith J.; Schaeffler B. A.; Biondi D. M.; Hirman J.; Pederson S.; Allan B.; Cady R. Efficacy and safety of eptinezumab in patients with chronic migraine: PROMISE-2. Neurology 2020, 94, E1365–E1377. 10.1212/WNL.0000000000009169. 32209650 PMC7274916

[ref23] Bangs M. E.; Kudrow D.; Wang S.; Oakes T. M.; Terwindt G. M.; Magis D.; Yunes-Medina L.; Stauffer V. L. Safety and tolerability of monthly galcanezumab injections in patients with migraine: integrated results from migraine clinical studies. BMC Neurol. 2020, 20, 25 10.1186/s12883-020-1609-7. 31952501 PMC6966798

[ref24] Archbold J. K.; Flanagan J. U.; Watkins H. A.; Gingell J. J.; Hay D. L. Structural insights into RAMP modification of secretin family G protein-coupled receptors: implications for drug development. Trends Pharmacol. Sci. 2011, 32, 591–600. 10.1016/j.tips.2011.05.007. 21722971

[ref25] Brain S. D.; Grant A. D. Vascular actions of calcitonin gene-related peptide and adrenomedullin. Physiol. Rev. 2004, 84, 903–934. 10.1152/physrev.00037.2003. 15269340

[ref26] Shindo T.; Sakurai T.; Kamiyoshi A.; Ichikawa-Shindo Y.; Shimoyama N.; Iinuma N.; Arai T.; Miyagawa S. Regulation of adrenomedullin and its family peptide by RAMP system - lessons from genetically engineered mice. Curr. Protein Pept. Sci. 2013, 14, 347–357. 10.2174/13892037113149990052. 23745699

[ref27] Larráyoz I. M.; Martínez-Herrero S.; García-Sanmartín J.; Ochoa-Callejero L.; Martínez A. Adrenomedullin and tumour microenvironment. J. Transl. Med. 2014, 12, 339 10.1186/s12967-014-0339-2. 25475159 PMC4272513

[ref28] Hay D. L.; Walker C. S.; Poyner D. R. Adrenomedullin and calcitonin gene-related peptide receptors in endocrine-related cancers: opportunities and challenges. Endocr.-Relat. Cancer 2011, 18, C1–C14. 10.1677/ERC-10-0244. 21051558

[ref29] Qiao F.; Fang J.; Xu J.; Zhao W.; Ni Y.; Akuo B. A.; Zhang W.; Liu Y.; Ding F.; Li G.; Liu B.; Wang H.; Shao S. The role of adrenomedullin in the pathogenesis of gastric cancer. Oncotarget 2017, 8, 88464–88474. 10.18632/oncotarget.18881. 29179449 PMC5687619

[ref30] Dai K.; Tanaka M.; Kamiyoshi A.; Sakurai T.; Ichikawa-Shindo Y.; Kawate H.; Cui N.; Wei Y.; Tanaka M.; Kakihara S.; Matsui S.; Shindo T. Deficiency of the adrenomedullin-RAMP3 system suppresses metastasis through the modification of cancer-associated fibroblasts. Oncogene 2020, 39, 1914–1930. 10.1038/s41388-019-1112-z. 31754214

[ref31] Greillier L.; Tounsi A.; Berenguer-Daize C.; Dussault N.; Delfino C.; Benyahia Z.; Cayol M.; Mabrouk K.; Garcia S.; Martin P. M.; Barlesi F.; Ouafik L. Functional analysis of the adrenomedullin pathway in malignant pleural mesothelioma. J. Thorac. Oncol. 2016, 11, 94–107. 10.1016/j.jtho.2015.09.004. 26762744

[ref32] Brekhman V.; Lugassie J.; Zaffryar-Eilot S.; Sabo E.; Kessler O.; Smith V.; Golding H.; Neufeld G. Receptor activity modifying protein-3 mediates the protumorigenic activity of lysyl oxidase-like protein-2. FASEB J. 2011, 25, 55–65. 10.1096/fj.10-162677. 20802105

[ref33] Siclari V. A.; Mohammad K. S.; Tompkins D. R.; Davis H.; McKenna C. R.; Peng X.; Wessner L. L.; Niewolna M.; Guise T. A.; Suvannasankha A.; Chirgwin J. M. Tumor-expressed adrenomedullin accelerates breast cancer bone metastasis. Breast Cancer Res. 2014, 16, 458 10.1186/s13058-014-0458-y. 25439669 PMC4303191

[ref34] Avgoustou P.; Jailani A. B. A.; Zirimwabagabo J.-O.; Tozer M. J.; Gibson K. R.; Glossop P. A.; Mills J. E. J.; Porter R. A.; Blaney P.; Bungay P.; Wang N.; Shaw A.; Bigos K. J. A.; Holmes J. L.; Warrington J. I.; Skerry T. M.; Harrity J. P. A.; Richards G. O. Discovery of a first-in-class potent small molecule antagonist against the adrenomedullin-2 receptor. ACS Pharmacol. Transl. Sci. 2020, 3, 706–719. 10.1021/acsptsci.0c00032. 32832872 PMC7432679

[ref35] Kusano S.; Kukimoto-Niino M.; Hino N.; Ohsawa N.; Okuda K.-i.; Sakamoto K.; Shirouzu M.; Shindo T.; Yokoyama S. Structural basis for extracellular interactions between calcitonin receptor-like receptor and receptor activity-modifying protein 2 for adrenomedullin-specific binding. Protein Sci. 2012, 21, 199–210. 10.1002/pro.2003. 22102369 PMC3324764

[ref36] ter Haar E. Crystal structure of the ectodomain complex of the CGRP receptor, a Class-B GPCR, reveals the site of drug antagonism. Structure 2010, 18, 1083–1093. 10.1016/j.str.2010.05.014. 20826335

[ref37] O’Boyle N. M.; Banck M.; James C. A.; Morley C.; Vandermeersch T.; Hutchison G. R. Open babel: an open chemical toolbox. J. Cheminf. 2011, 3, 33 10.1186/1758-2946-3-33. PMC3198950 21982300

[ref38] Crowley B. M.; Stump C. A.; Nguyen D. N.; Potteiger C. M.; McWherter M. A.; Paone D. V.; Quigley A. G.; Bruno J. G.; Cui D.; Culberson J. C.; Danziger A.; Fandozzi C.; Gauvreau D.; Kemmerer A. L.; Menzel K.; Moore E. L.; Mosser S. D.; Reddy V.; White R. B.; Salvatore C. A.; Kane S. A.; Bell I. M.; Selnick H. G.; Fraley M. E.; Burgey C. S. Novel oxazolidinone calcitonin gene-related peptide (CGRP) receptor antagonists for the acute treatment of migraine. Bioorg. Med. Chem. Lett. 2015, 25, 4777–4781. 10.1016/j.bmcl.2015.07.021. 26231160

[ref39] Neese F. The ORCA program system. Wiley Interdiscip. Rev.: Comput. Mol. Sci. 2012, 2, 73–78. 10.1002/wcms.81.

[ref40] Jones G.; Willett P.; Glen R. C.; Leach A. R.; Taylor R. Development and validation of a genetic algorithm for flexible docking. J. Mol. Biol. 1997, 267, 727–748. 10.1006/jmbi.1996.0897. 9126849

[ref41] Wood M. R.; Schirripa K. M.; Kim J. J.; Quigley A. G.; Stump C. A.; Bell I. M.; Bednar R. A.; Fay J. F.; Bruno J. G.; Moore E. L.; Mosser S. D.; Roller S.; Salvatore C. A.; Kane S. A.; Vacca J. P.; Selnick H. G. Novel CGRP receptor antagonists through a design strategy of target simplification with addition of molecular flexibility. Bioorg. Med. Chem. Lett. 2009, 19, 5787–5790. 10.1016/j.bmcl.2009.07.134. 19703767

[ref42] Booe J. M.; Walker C. S.; Barwell J.; Kuteyi G.; Simms J.; Jamaluddin M. A.; Warner M. L.; Bill R. M.; Harris P. W.; Brimble M. A.; Poyner D. R.; Hay D. L.; Pioszak A. A. Structural basis for receptor activity-modifying protein-dependent selective peptide recognition by a G protein-coupled receptor. Mol. Cell 2015, 58, 1040–1052. 10.1016/j.molcel.2015.04.018. 25982113 PMC4504005

[ref43] Bell I. M.; Gallicchio S. N.; Wood M. R.; Quigley A. G.; Stump C. A.; Zartman C. B.; Fay J. F.; Li C. C.; Lynch J. J.; Moore E. L.; Mosser S. D.; Prueksaritanont T.; Regan C. P.; Roller S.; Salvatore C. A.; Kane S. A.; Vacca J. P.; Selnick H. G. Discovery of MK-3207: a highly potent, orally bioavailable CGRP receptor antagonist. ACS Med. Chem. Lett. 2010, 1, 24–29. 10.1021/ml900016y. 24900170 PMC4007836

[ref44] Rudolf K.; Eberlein W.; Engel W.; Pieper H.; Entzeroth M.; Hallermayer G.; Doods H. Development of human calcitonin gene-related peptide (CGRP) receptor antagonists. 1. Potent and selective small molecule CGRP antagonists. 1-[*N*^2^-[3,5-Dibromo-*N*-[[4-(3,4-dihydro-2(1*H*)-oxoquinazolin-3-yl)-1-piperidinyl]carbonyl]-D-tyrosyl]-L-lysyl]-4-(4-pyridinyl)piperazine: the first CGRP antagonist for clinical trials in acute migraine. J. Med. Chem. 2005, 48, 5921–5931. 10.1021/jm0490641. 16161996

[ref45] Belyk K. M.; Cleator E.; Kuo S.-C.; Maligres P. E.; Xiang B.; Yasuda N.; Yin J. Process for Making CGRP Receptor Antagonists. U.S. Patent US9,487,523, 2016.

[ref46] Xiang B.; Belyk K. M.; Reamer R. A.; Yasuda N. Discovery and application of doubly quaternized cinchona-alkaloid-based phase-transfer catalysts. Angew. Chem., Int. Ed. 2014, 53, 8375–8378. 10.1002/anie.201404084. 24961909

[ref47] Dräger G.; Solodenko W.; Messinger J.; Schon U.; Kirschning A. A new reagent and its polymer-supported variant for the amidination of amines. Tetrahedron Lett. 2002, 43, 1401–1403. 10.1016/S0040-4039(01)02403-0.

