# Supplementary material for: Discovery of a First-In-Class Small Molecule Antagonist against the Adrenomedullin-2 Receptor: Structure–Activity Relationships and Optimization
Source: J Med Chem. 2021 Mar 5;64(6):3299–319. doi: 10.1021/acs.jmedchem.0c02191 (PMC8006142; doi:10.1021/acs.jmedchem.0c02191)
Supplement: Supplementary file 6 — jm0c02191_si_006.pdf [file jm0c02191_si_006.pdf]

Supporting Information

Discovery of a First-in-Class Small Molecule Antagonist Against the Adrenomedullin-2 Receptor: Structure-Activity Relationships and Optimization

Jean-Olivier Zirimwabagabo[1#], Ameera B. A. Jailani[2#], Paris Avgoustou[2#], Matthew J. Tozer[3], Karl R. Gibson[4], Paul A. Glossop[4], James E. J. Mills[4], Roderick A. Porter[5], Paul Blaney[6], Ning Wang[2], Timothy M. Skerry[2#], Gareth O. Richards[2#] and Joseph P. A. Harrity[1*,#]

1. Department of Chemistry, University of Sheffield, S10 2TN, UK
2. Department of Oncology and Metabolism, University of Sheffield, S10 2TN, UK
3. Matt Tozer Consultancy, Bognor Regis, PO21 1DY, UK
4. Sandexis Medicinal Chemistry Ltd, Sandwich, Kent, CT13 9ND, UK
5. Rod Porter Consultancy, Ashwell, Hertfordshire, SG7 5PG, UK
6. Concept Life Sciences, High Peak, SK23 0PG, UK

[#] These authors contributed equally to the work

*Correspondence to Joseph P. A. Harrity
Email: j.harrity@sheffield.ac.uk

Table of Contents

1. X-Ray crystal structure analysis of (*R*)-CLR binding unit **30**……………………………..…S2

2. ^{1}H NMR /LCMS/HPLC Traces of Compound **12**………………………………………….S11

3. ^{1}H NMR /LCMS/HPLC Traces of Compound **14**………………………………………….S14

4. ^{1}H NMR /LCMS/HPLC Traces of Compound (±)-**25**……………………………………...S16

5. ^{1}H NMR /LCMS/HPLC Traces of Compound (*R*)-**25**……………………………………..S18

X-Ray crystal structure analysis of (*R*)-CLR binding unit 30

This compound was crystallised from methanol through slow evaporation at room temperature. The requisite CIF has been deposited with the **CCDC 2027033** contains the supplementary crystallographic data for this paper. These data are provided free of charge by the joint Cambridge Crystallographic Data Centre and Fachinformationszentrum Karlsruhe Access Structures service www.ccdc.cam.ac.uk/structures.

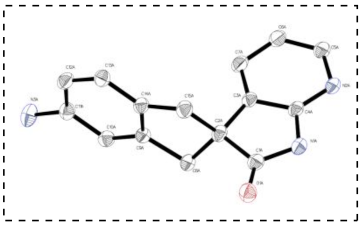

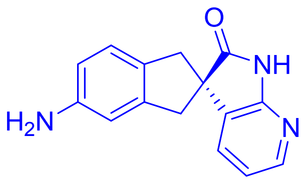

Table 1 Crystal data and structure refinement for 2018ncs0092s.

Identification code	2018ncs0092s
Empirical formula	$C_{15}H_{13}N_3O$
Formula weight	251.28
Temperature/K	99.99(10)
Crystal system	monoclinic
Space group	$P2_1$
a/Å	8.71373(14)
b/Å	15.1311(2)
c/Å	9.8448(2)
α/°	90
β/°	113.968(2)
γ/°	90
Volume/Å^3	1186.09(4)
Z	4

ρ_{calc}g/cm^3	1.407
μ/mm^{-1}	0.735
F(000)	528.0
Crystal size/mm^3	0.19 × 0.05 × 0.02
Radiation	CuKα (λ = 1.54178)
2Θ range for data collection/°	9.832 to 136.488
Index ranges	-10 ≤ h ≤ 10, -18 ≤ k ≤ 18, -11 ≤ l ≤ 11
Reflections collected	15858
Independent reflections	4251 [R_{int} = 0.0388, R_{sigma} = 0.0337]
Data/restraints/parameters	4251/1/367
Goodness-of-fit on F^2	1.133
Final R indexes [I>=2σ (I)]	R_1 = 0.0370, wR_2 = 0.1018
Final R indexes [all data]	R_1 = 0.0394, wR_2 = 0.1036
Largest diff. peak/hole / e Å^{-3}	0.15/-0.19
Flack parameter	0.01(17)

Table 2 Fractional Atomic Coordinates (×10^4) and Equivalent Isotropic Displacement Parameters (Å^2×10^3) for 2018ncs0092s. U_{eq} is defined as 1/3 of of the trace of the orthogonalised U_{IJ} tensor.

Atom	*x*	*y*	*z*	U(eq)
O1A	-804(3)	7015.7(16)	7338(3)	30.0(6)
N1A	-1450(3)	5967(2)	8720(3)	23.9(6)
N2A	-1494(3)	4654.8(18)	10065(3)	22.8(6)
N3A	3022(3)	3642(2)	3543(3)	32.8(6)
C1A	-722(4)	6264(2)	7804(3)	23.0(7)

C2A	244(4)	5491(2)	7508(3)	22.3(7)
C3A	-27(3)	4765(2)	8429(3)	21.0(7)
C4A	-1027(3)	5090(2)	9126(3)	21.5(7)
C5A	-919(4)	3808(2)	10333(3)	24.2(7)
C6A	40(4)	3419(2)	9687(4)	27.1(8)
C7A	505(4)	3903(2)	8699(4)	25.9(8)
C8A	-384(4)	5247(2)	5837(4)	23.1(7)
C9A	1174(4)	4880(2)	5726(4)	21.8(7)
C10A	1287(4)	4373(2)	4600(3)	23.3(7)
C11A	2880(4)	4126(2)	4693(3)	23.7(7)
C12A	4312(4)	4394(2)	5915(4)	27.2(8)
C13A	4180(4)	4911(2)	7029(4)	25.5(8)
C14A	2600(4)	5151(2)	6935(3)	22.7(7)
C15A	2136(4)	5713(2)	7969(3)	25.9(8)
O1B	5965(3)	4516.4(16)	2856(3)	28.6(5)
N1B	6371(3)	5526(2)	1286(3)	23.8(6)
N2B	6375(3)	6839.8(18)	-73(3)	23.7(6)
N3B	-1043(3)	7369.4(18)	3878(3)	29.9(6)
C1B	5741(4)	5248(2)	2293(3)	22.8(7)
C2B	4744(3)	6013(2)	2567(3)	22.3(7)
C3B	4983(3)	6735(2)	1620(3)	21.6(7)
C4B	5930(3)	6404(2)	881(3)	21.2(7)
C5B	5827(4)	7687(2)	-327(3)	24.3(7)
C6B	4916(4)	8086(2)	361(4)	26.2(8)
C7B	4472(4)	7602(2)	1364(4)	24.8(7)
C8B	2845(3)	5787(2)	2080(3)	23.0(7)

C9B	2355(4)	6358(2)	3094(3)	21.7(7)
C10B	760(4)	6606(2)	2930(4)	22.4(7)
C11B	565(3)	7131(2)	4011(3)	22.5(7)
C12B	1986(4)	7394(2)	5257(4)	26.8(8)
C13B	3579(4)	7124(2)	5425(3)	25.6(7)
C14B	3776(4)	6623(2)	4326(3)	22.6(7)
C15B	5348(4)	6263(2)	4242(3)	23.7(7)

Table 3 Anisotropic Displacement Parameters (Å^2×10^3) for 2018ncs0092s. The Anisotropic displacement factor exponent takes the form: -2π^2[h^{2}a*^{2}U$_{11}$+2hka*b*U$_{12}$+…].

Atom	U_{11}	U_{22}	U_{33}	U_{23}	U_{13}	U_{12}
O1A	33.6(12)	25.4(14)	35.8(13)	1.5(10)	19.2(10)	0.5(10)
N1A	21.4(13)	25.1(15)	26.6(14)	-1.8(12)	11.0(11)	2.6(11)
N2A	15.9(12)	26.2(16)	26.1(13)	-1.0(12)	8.3(10)	0.4(11)
N3A	30.3(13)	33.2(14)	40.8(15)	-11.2(12)	20.6(12)	-3.0(12)
C1A	19.2(15)	24.6(18)	24.0(16)	-1.5(14)	7.6(12)	-1.3(13)
C2A	17.3(13)	25.9(18)	23.7(15)	-2.7(13)	8.2(12)	-1.4(13)
C3A	12.7(13)	26.9(19)	21.6(15)	-0.8(13)	5.1(12)	-0.5(12)
C4A	14.7(12)	25.9(19)	22.0(15)	-2.3(14)	5.6(11)	0.3(13)
C5A	18.7(14)	27(2)	26.1(16)	0.4(15)	8.3(13)	-0.9(13)
C6A	22.7(15)	26.4(19)	32.3(19)	2.6(14)	11.3(14)	3.5(13)
C7A	19.3(15)	30(2)	27.7(16)	-0.5(14)	9.1(13)	4.1(13)
C8A	18.6(14)	26(2)	25.6(16)	0.8(14)	10.2(13)	-0.1(13)
C9A	19.4(14)	23.7(18)	22.9(16)	2.4(13)	9.2(12)	1.3(12)

C10A	20.9(14)	23.1(18)	25.1(16)	0.7(14)	8.5(12)	-1.0(12)
C11A	26.1(15)	21.3(16)	27.2(16)	-1.0(14)	14.3(13)	-0.2(13)
C12A	19.7(14)	28(2)	35.5(18)	5.8(15)	13.2(14)	3.2(13)
C13A	19.6(14)	31(2)	26.8(18)	2.2(14)	10.3(13)	-1.5(13)
C14A	19.2(14)	23.7(19)	26.3(16)	2.7(14)	10.2(12)	-0.5(13)
C15A	19.8(15)	32(2)	26.2(17)	-1.8(15)	9.4(13)	-2.5(14)
O1B	26.2(11)	27.8(14)	34.5(13)	4.3(11)	15(1)	2.5(10)
N1B	20.3(12)	25.2(15)	28.9(14)	1.6(12)	13.0(11)	1.6(11)
N2B	19.3(12)	27.7(17)	24.5(14)	-1.0(12)	9.3(10)	-1.5(11)
N3B	21.0(12)	36.8(15)	32.7(14)	-2.8(12)	11.7(11)	4.7(10)
C1B	15.5(13)	29(2)	24.0(16)	-1.9(14)	8.4(12)	-1.2(13)
C2B	16.2(14)	25.9(19)	26.2(16)	0.4(14)	10.2(13)	0.3(13)
C3B	12.6(13)	28.4(19)	22.0(16)	-3.4(13)	5.1(12)	-0.1(13)
C4B	13.2(13)	23.1(18)	24.0(15)	-2.4(14)	4.3(12)	-0.8(12)
C5B	20.5(16)	27(2)	24.7(17)	2.1(14)	8.8(13)	-1.3(13)
C6B	24.0(14)	25.1(19)	28.7(19)	-0.7(14)	9.9(14)	1.4(14)
C7B	18.7(15)	30(2)	26.6(16)	-1.8(14)	9.9(13)	2.2(13)
C8B	17.2(15)	29(2)	24.1(16)	-3.3(14)	9.4(13)	-2.1(13)
C9B	19.9(14)	24.8(19)	20.6(15)	0.2(13)	8.4(12)	-2.0(13)
C10B	16.3(14)	25.3(19)	24.3(17)	-0.6(13)	6.9(12)	-1.8(12)
C11B	20.4(14)	24.4(19)	25.2(16)	1.9(14)	11.8(12)	0.6(12)
C12B	27.0(15)	27.4(19)	29.8(17)	-1.9(15)	15.3(14)	-1.0(14)
C13B	22.4(14)	32(2)	22.1(15)	-4.4(15)	8.4(12)	-4.5(13)
C14B	16.9(14)	27.0(19)	22.6(17)	0.6(13)	6.7(12)	-3.2(13)
C15B	18.6(15)	32(2)	20.1(16)	-0.9(14)	7.2(12)	-1.7(14)

Table 4 Bond Lengths for 2018ncs0092s.

Atom	Atom	Length/Å	Atom	Atom	Length/Å
O1A	C1A	1.218(4)	O1B	C1B	1.218(4)
N1A	C1A	1.371(4)	N1B	C1B	1.379(4)
N1A	C4A	1.393(4)	N1B	C4B	1.397(4)
N2A	C4A	1.326(4)	N2B	C4B	1.327(4)
N2A	C5A	1.362(4)	N2B	C5B	1.356(4)
N3A	C11A	1.396(4)	N3B	C11B	1.400(4)
C1A	C2A	1.536(4)	C1B	C2B	1.535(4)
C2A	C3A	1.503(5)	C2B	C3B	1.505(5)
C2A	C8A	1.552(4)	C2B	C8B	1.562(4)
C2A	C15A	1.558(4)	C2B	C15B	1.560(4)
C3A	C4A	1.399(4)	C3B	C4B	1.396(4)
C3A	C7A	1.374(5)	C3B	C7B	1.375(5)
C5A	C6A	1.372(4)	C5B	C6B	1.375(4)
C6A	C7A	1.402(5)	C6B	C7B	1.404(4)
C8A	C9A	1.511(4)	C8B	C9B	1.507(4)
C9A	C10A	1.384(4)	C9B	C10B	1.385(4)
C9A	C14A	1.388(4)	C9B	C14B	1.395(4)
C10A	C11A	1.404(4)	C10B	C11B	1.392(4)
C11A	C12A	1.396(4)	C11B	C12B	1.401(4)
C12A	C13A	1.389(5)	C12B	C13B	1.391(4)
C13A	C14A	1.390(4)	C13B	C14B	1.388(4)
C14A	C15A	1.502(4)	C14B	C15B	1.507(4)

Table 5 Bond Angles for 2018ncs0092s.

Atom	Atom	Atom	Angle/˚	Atom	Atom	Atom	Angle/˚
C1A	N1A	C4A	111.2(3)	C1B	N1B	C4B	110.6(3)
C4A	N2A	C5A	114.2(3)	C4B	N2B	C5B	114.7(3)
O1A	C1A	N1A	125.5(3)	O1B	C1B	N1B	124.5(3)
O1A	C1A	C2A	126.3(3)	O1B	C1B	C2B	127.1(3)
N1A	C1A	C2A	108.2(3)	N1B	C1B	C2B	108.4(3)
C1A	C2A	C8A	113.4(3)	C1B	C2B	C8B	112.7(3)
C1A	C2A	C15A	112.2(3)	C1B	C2B	C15B	113.4(3)
C3A	C2A	C1A	102.0(2)	C3B	C2B	C1B	102.0(2)
C3A	C2A	C8A	113.0(3)	C3B	C2B	C8B	111.4(2)
C3A	C2A	C15A	112.9(3)	C3B	C2B	C15B	114.0(3)
C8A	C2A	C15A	103.7(2)	C15B	C2B	C8B	103.8(2)
C4A	C3A	C2A	108.7(3)	C4B	C3B	C2B	108.8(3)
C7A	C3A	C2A	133.4(3)	C7B	C3B	C2B	133.7(3)
C7A	C3A	C4A	117.8(3)	C7B	C3B	C4B	117.5(3)
N1A	C4A	C3A	109.8(3)	N2B	C4B	N1B	123.3(3)
N2A	C4A	N1A	123.6(3)	N2B	C4B	C3B	126.6(3)
N2A	C4A	C3A	126.6(3)	C3B	C4B	N1B	110.1(3)
N2A	C5A	C6A	124.0(3)	N2B	C5B	C6B	123.7(3)
C5A	C6A	C7A	120.0(3)	C5B	C6B	C7B	119.8(3)
C3A	C7A	C6A	117.4(3)	C3B	C7B	C6B	117.7(3)
C9A	C8A	C2A	102.9(2)	C9B	C8B	C2B	102.8(2)
C10A	C9A	C8A	128.3(3)	C10B	C9B	C8B	128.3(3)
C10A	C9A	C14A	121.3(3)	C10B	C9B	C14B	121.2(3)
C14A	C9A	C8A	110.3(3)	C14B	C9B	C8B	110.5(3)

C9A	C10A	C11A	119.1(3)	C9B	C10B	C11B	119.6(3)
N3A	C11A	C10A	119.9(3)	N3B	C11B	C12B	120.2(3)
N3A	C11A	C12A	120.5(3)	C10B	C11B	N3B	120.2(3)
C12A	C11A	C10A	119.5(3)	C10B	C11B	C12B	119.5(3)
C13A	C12A	C11A	120.8(3)	C13B	C12B	C11B	120.3(3)
C12A	C13A	C14A	119.5(3)	C14B	C13B	C12B	120.1(3)
C9A	C14A	C13A	119.8(3)	C9B	C14B	C15B	110.7(3)
C9A	C14A	C15A	110.8(3)	C13B	C14B	C9B	119.2(3)
C13A	C14A	C15A	129.4(3)	C13B	C14B	C15B	130.1(3)
C14A	C15A	C2A	103.0(2)	C14B	C15B	C2B	102.8(2)

Table 6 Hydrogen Atom Coordinates (Å×10^4) and Isotropic Displacement Parameters (Å^2×10^3) for 2018ncs0092s.

Atom	*x*	*y*	*z*	U(eq)
H5A	-1194	3473	10995	29
H6A	382	2835	9904	33
H7A	1149	3650	8245	31
H8AA	-1269	4807	5558	28
H8AB	-796	5764	5211	28
H10A	322	4199	3793	28
H12A	5367	4225	5984	33
H13A	5141	5095	7831	31
H15A	2293	6336	7828	31
H15B	2798	5557	8998	31
H5B	6082	8018	-1005	29

H6B	4595	8675	163	31
H7B	3853	7858	1839	30
H8BA	2199	5937	1045	28
H8BB	2689	5165	2227	28
H10B	-176	6424	2103	27
H12B	1864	7750	5977	32
H13B	4513	7280	6274	31
H15C	5759	5749	4875	28
H15D	6226	6707	4527	28
H3AA	4100(50)	3490(30)	3630(40)	46(10)
H3BA	-1910(50)	7370(30)	2980(50)	51(11)
H3BB	-1110(50)	7810(30)	4470(40)	52(12)
H3AB	2360(50)	3190(30)	3210(50)	56(12)
H1B	7060(50)	5170(30)	950(50)	49(12)
H1A	-2150(50)	6300(30)	9040(40)	51(12)

Crystal structure determination of 2018ncs0092s

Crystal Data for $C_{15}H_{13}N_3O$ (M =251.28 g/mol): monoclinic, space group $P2_1$ (no. 4), a = 8.71373(14) Å, b = 15.1311(2) Å, c = 9.8448(2) Å, β = 113.968(2)°, V = 1186.09(4) Å^3, Z = 4, T = 99.99(10) K, μ(CuKα) = 0.735 mm^{-1}, $Dcalc$ = 1.407 g/cm^3, 15858 reflections measured (9.832° ≤ 2Θ ≤ 136.488°), 4251 unique (R_{int} = 0.0388, R_{sigma} = 0.0337) which were used in all calculations. The final R_1 was 0.0370 (I > 2σ(I)) and wR_2 was 0.1036 (all data).

2. ^{1}H NMR /LCMS/HPLC Traces of Compound 12

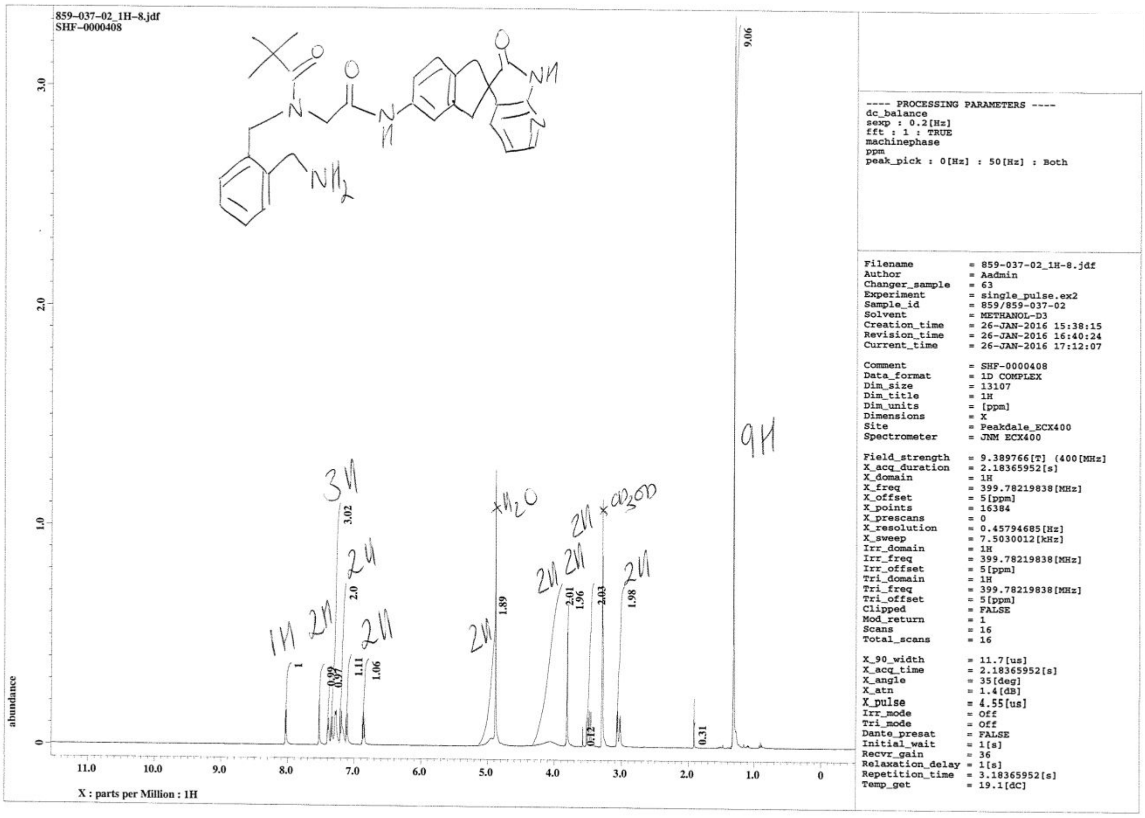

Peakdale Molecular Limited
Openlynx Report UPLC2 - XBC18 LONG BASE 2to98 METHOD Keith Statham

XBridge C18 2.5um
2.1 x 50mm column

C: 10mM NH4HCO3 @ pH10
B: MeCN

2-98% B in 4min hold @ 98% B
to 4.6min

Batch:Keith Statham240-1
Date:26-Jan-2016
Description:SHF-0000408

ID:859-037-02
Time:15:26:12

Vial:2:27
Method:C:\MassLynx\Long basic 2to98.olp

Printed: Tue Jan 26 15:32:46 2016

Sample Report:

Sample 1 Vial 2:27 ID 859-037-02 File Keith Statham240-1 Date 26-Jan-2016 Time 15:26:12 Description SHF-0000408 Method C:\MassLyn

3: UV Detector: TIC

3.307e+1
Range: 3.555e+1

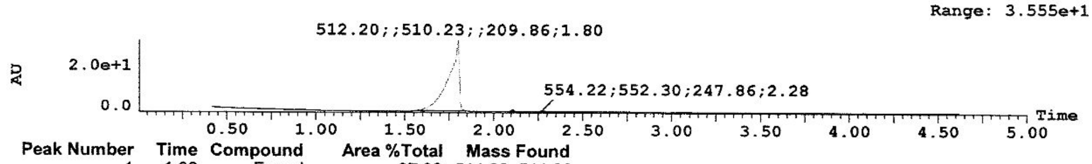

Peak Number	Time	Compound	Area %Total	Mass Found
1	1.80	Found	97.80	511.26, 511.26
3	2.11		0.75	
4	2.28		0.78	
5	2.46	Found	0.55	511.26
6	2.61		0.12	

1: MS ES+ :TIC

2.2e+008

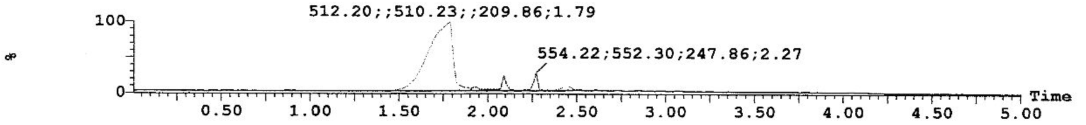

1: MS ES+ :512.26 1.0000Da

1.1e+008

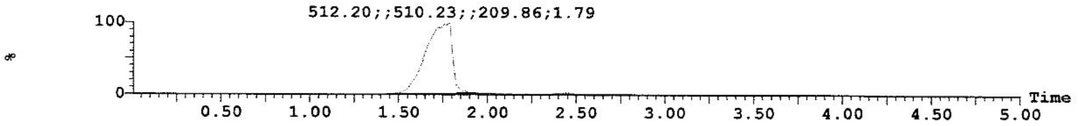

2: MS ES- :TIC

4.1e+006

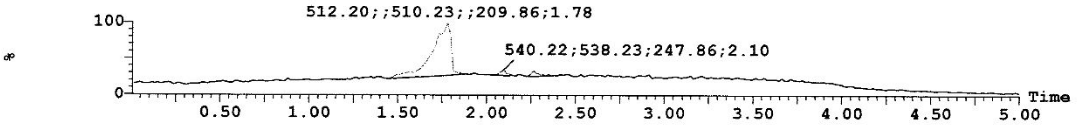

2: MS ES- :510.26 1.0000Da

2.1e+006

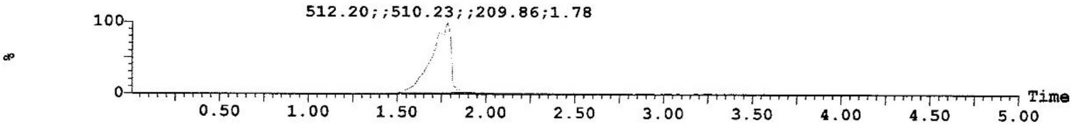

Peakdale Molecular Limited
Openlynx Report UPLC2 - XBC18 LONG BASE 2to98 METHOD Keith Statham

XBridge C18 2.5um<br>2.1 x 50mm column	C: 10mM NH4HCO3 @ pH10<br>B: MeCN	2-98% B in 4min hold @ 98% B<br>to 4.6min
Batch:Keith Statham240-1<br>Date:26-Jan-2016<br>Description:SHF-0000408	ID:859-037-02<br>Time:15:26:12	Vial:2:27<br>Method:C:\MassLynx\Long basic 2to98.olp

Printed: Tue Jan 26 15:32:46 2016

Sample Report (continued):

Time	Mass Found	Peak ID	Compound
1.79	512.26	1	Found

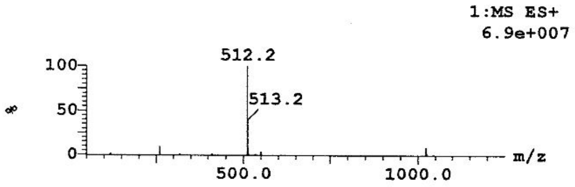

Time	Mass Found	Peak ID	Compound
1.79	510.26	1	Found

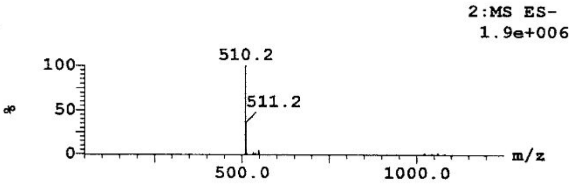

Time	Mass Found	Peak ID	Compound
1.94		2	

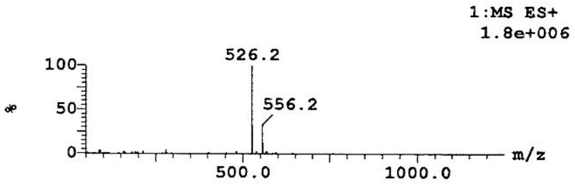

Time	Mass Found	Peak ID	Compound
2.09		3	

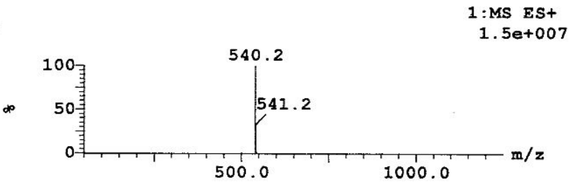

Time	Mass Found	Peak ID	Compound
2.27		4	

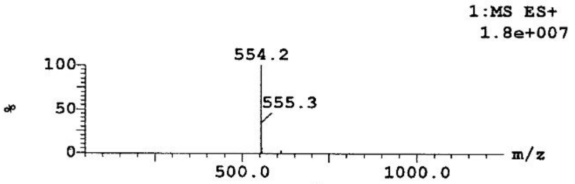

Time	Mass Found	Peak ID	Compound
2.44	512.26	5	Found

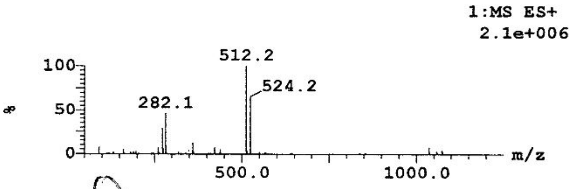

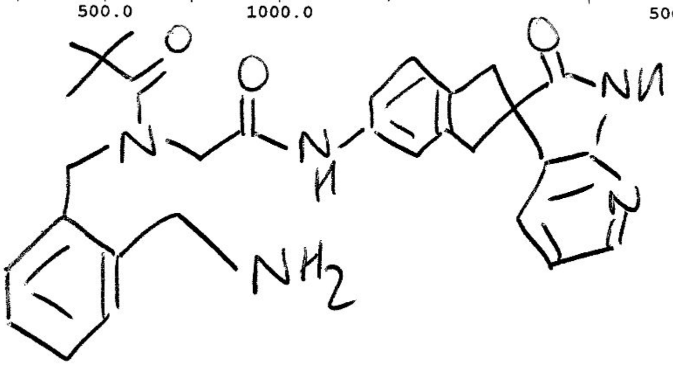

5

3. ^{1}H NMR /LCMS/HPLC Traces of Compound 14

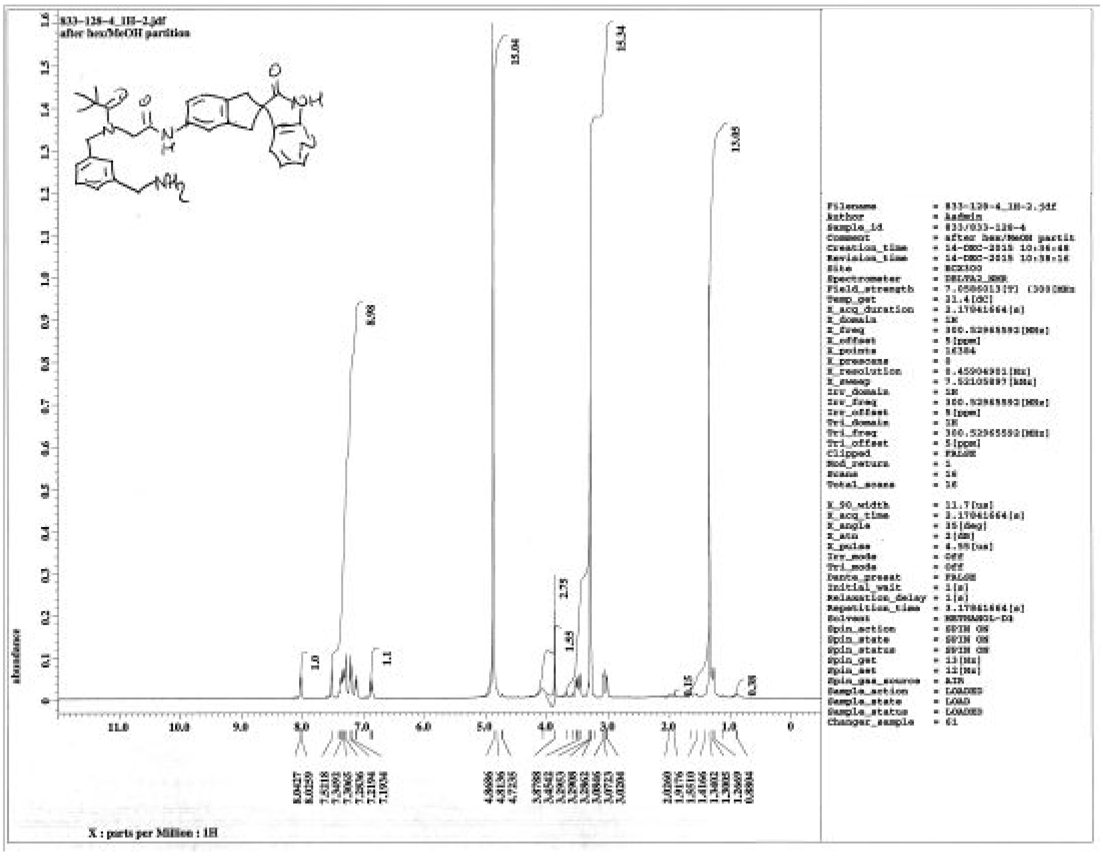

79985

RFA 79985
Method: 11m 1ml 5to95%C with D2
Sample Set: 79985b
Acquisition date: 12/14/2015 1:16:51 PM GMT
Column Type: XBC18 3x150 5um
Project Name: Yellowforms_Dec_15
Solvent D line:

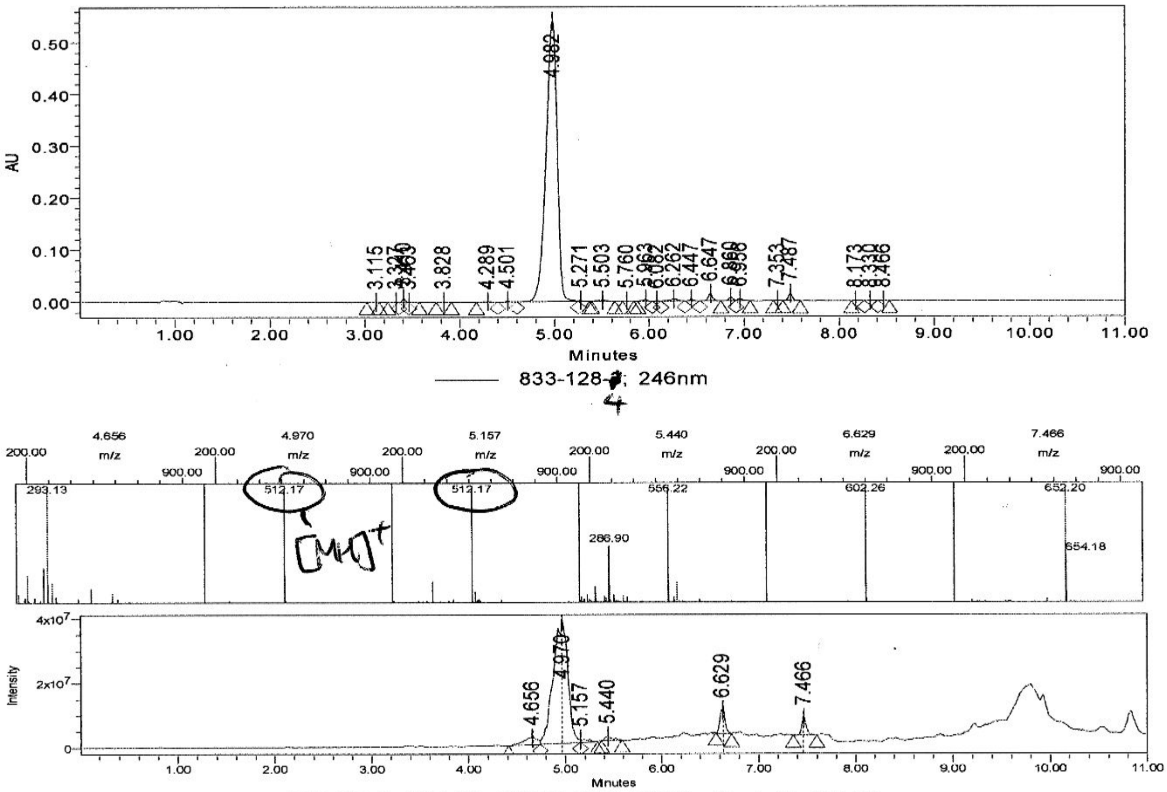

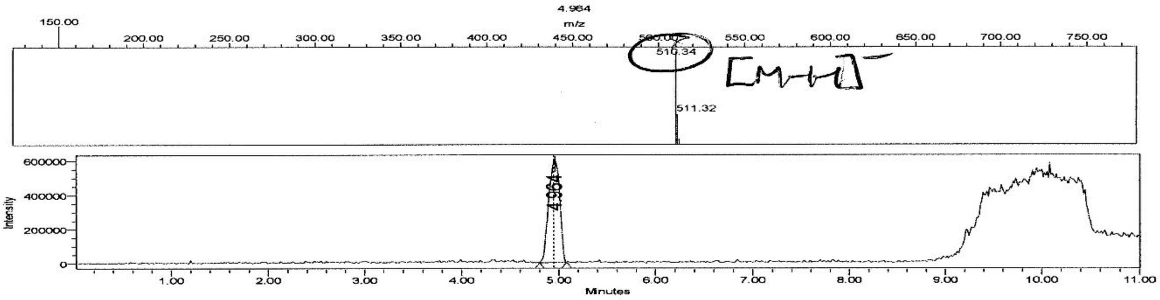

833-128-3; MS TIC; 100.00-1000.00 ES-, Centroid, CV=40

	Retention Time (min)	% Area
1	3.115	0.03
2	3.327	0.04
3	3.410	0.35
4	3.463	0.03
5	3.828	0.04
6	4.289	0.11
7	4.501	0.13
8	4.982	94.84
9	5.271	0.12
10	5.503	0.30
11	5.760	0.03
12	5.963	0.27
13	6.082	0.06
14	6.262	0.54
15	6.447	0.24
16	6.647	0.96
17	6.860	0.46
18	6.956	0.34
19	7.353	0.07
20	7.487	0.89
21	8.173	0.04
22	8.330	0.07
23	8.466	0.05

4. ^{1}H NMR /LCMS/HPLC Traces of Compound (±)-25

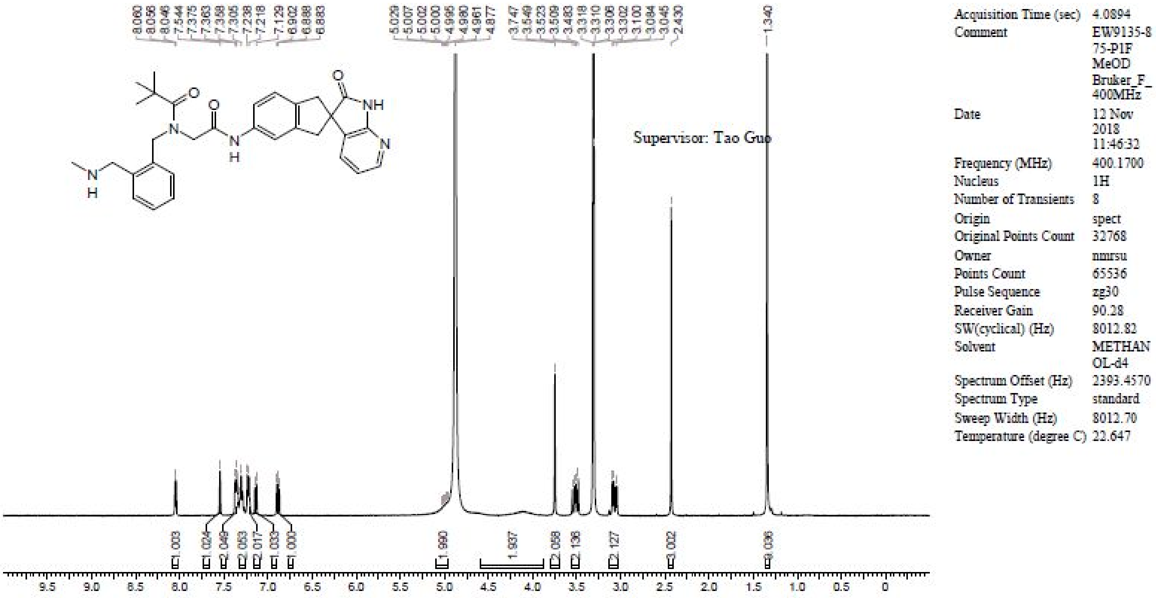

LCMS REPORT

Compound ID : SHF-638
Sample ID : EW9135-875-P1F2
Injection Vol : 1ul
Location : vial55
Acq Method : d:\method\0-60AB_4MIN_220&254.lcm
Org DataFile : D:\DATA\1811\181108\EW9135-875-P1F2.lcd
Injection Date : 2018-11-08 18:06:11
Instrument : LCMS-A 15-105

Chromatogram

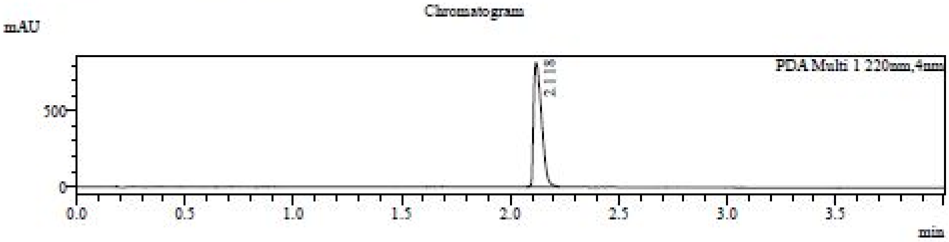

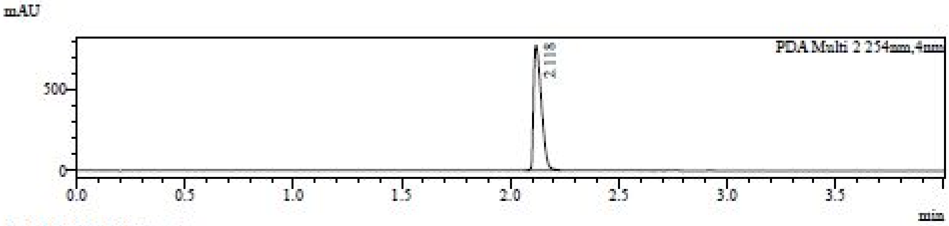

1 PDA Multi 1 / 220nm,4nm
2 PDA Multi 2 / 254nm,4nm

MS Chromatogram

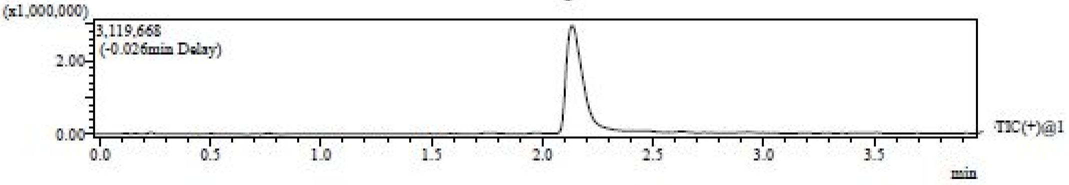

Integration Result

Peak Table

PDA Ch1 220nm

Peak#	Ret. Time	Height	Height%	USP Width	Area	Area%
1	2.118	818049	100.000	0.069	2103246	100.000

Peak Table

PDA Ch2 254nm

Peak#	Ret. Time	Height	Height%	USP Width	Area	Area%
1	2.118	770206	100.000	0.068	1917202	100.000

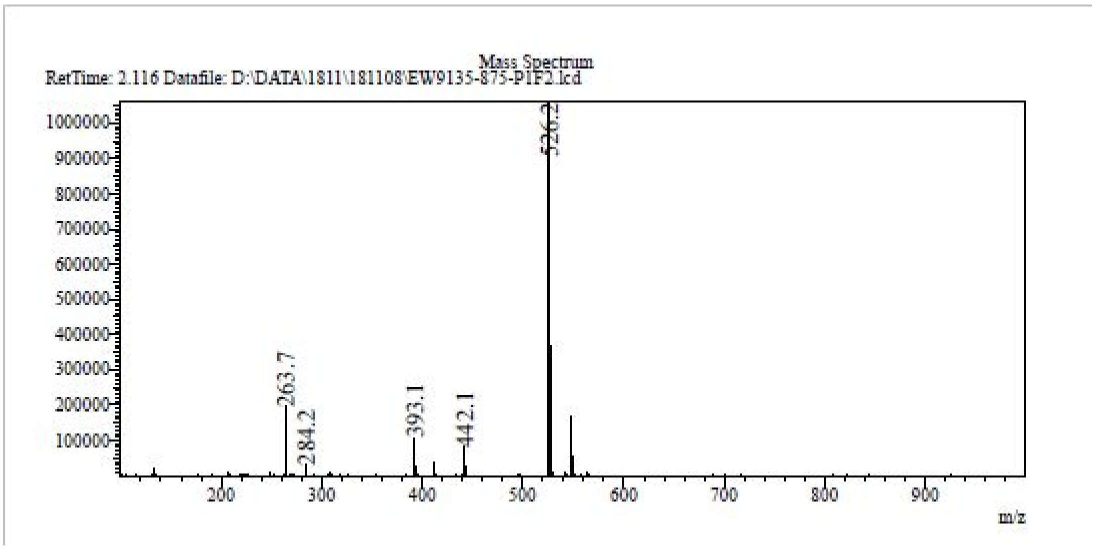

5. ¹H NMR /LCMS/HPLC Traces of Compound (*R*)-25

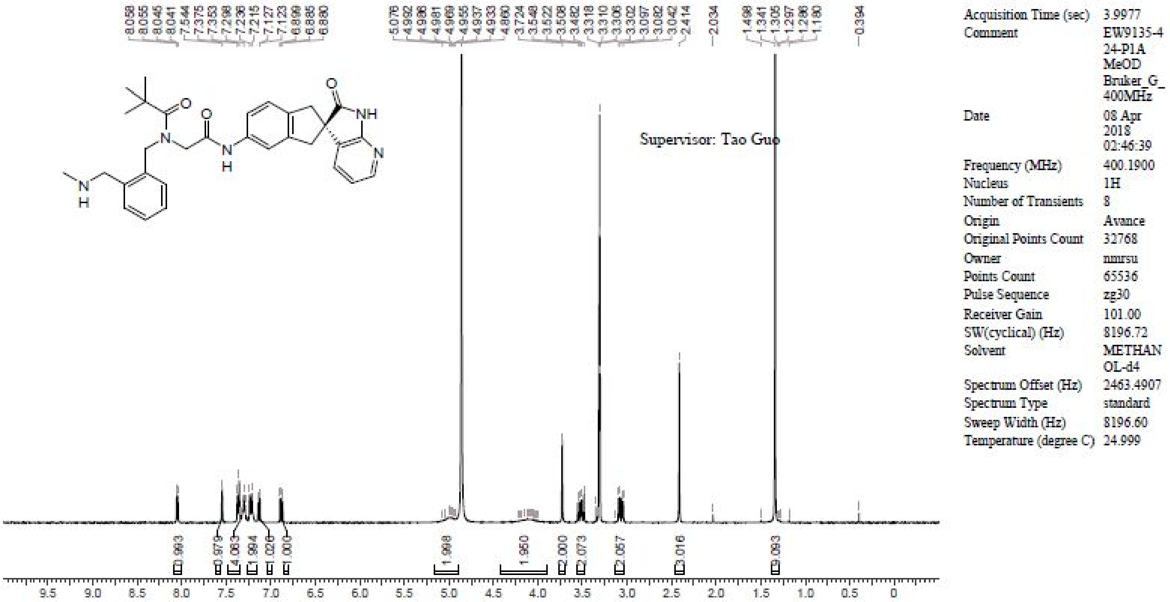

LCMS REPORT

Compound ID	: vSHF-771
Sample ID	: EW9135-424-P1A1
Injection Vol	: 1ul
Location	: vial94
Acq Method	: d:\method\0-60AB_4MIN_220&254.lcm
Org DataFile	: D:\DATA\1804\180408\EW9135-424-P1A1.lcd
Injection Date	: 2018-04-08 18:24:37
Instrument	: LCMS-A 15-105

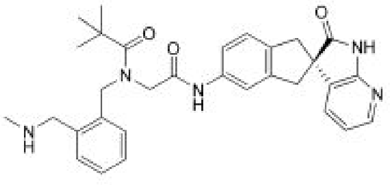

Chemical Formula: $C_{31}H_{35}N_5O_3$
Molecular Weight: 525.64

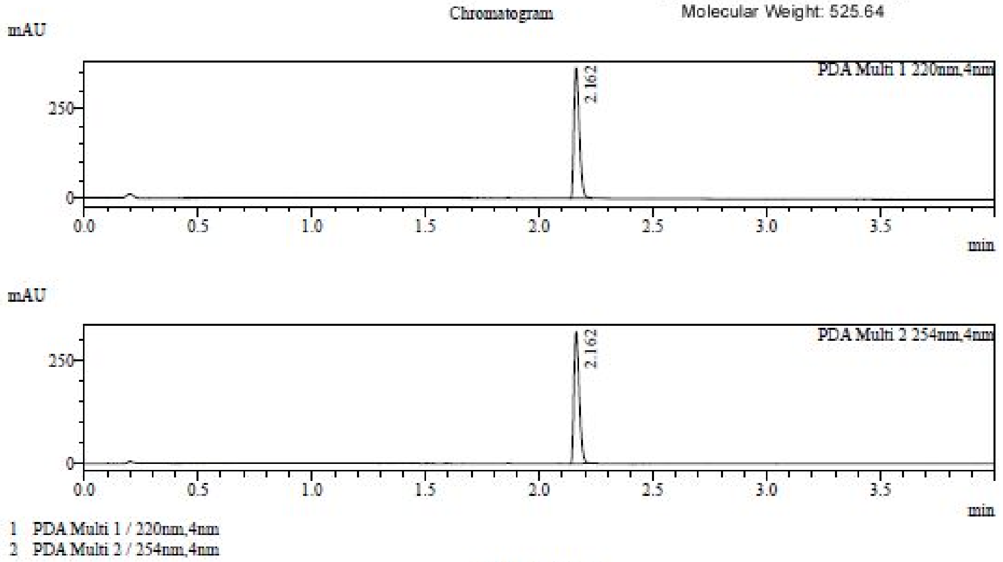

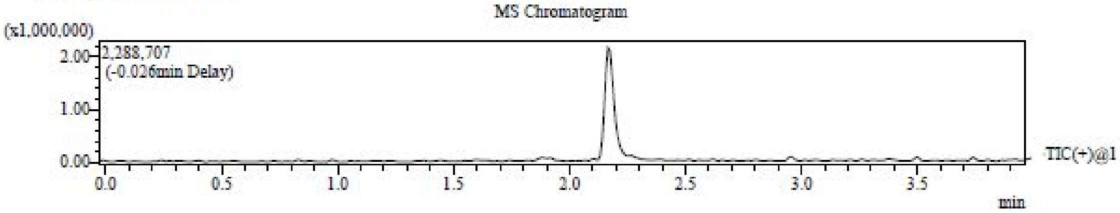

Integration Result

Peak Table

PDA Ch1 220nm

Peak#	Ret. Time	Height	Height%	USP Width	Area	Area%
1	2.162	364974	100.000	0.044	591308	100.000

Peak Table

PDA Ch2 254nm

Peak#	Ret. Time	Height	Height%	USP Width	Area	Area%
1	2.162	320982	100.000	0.044	515144	100.000

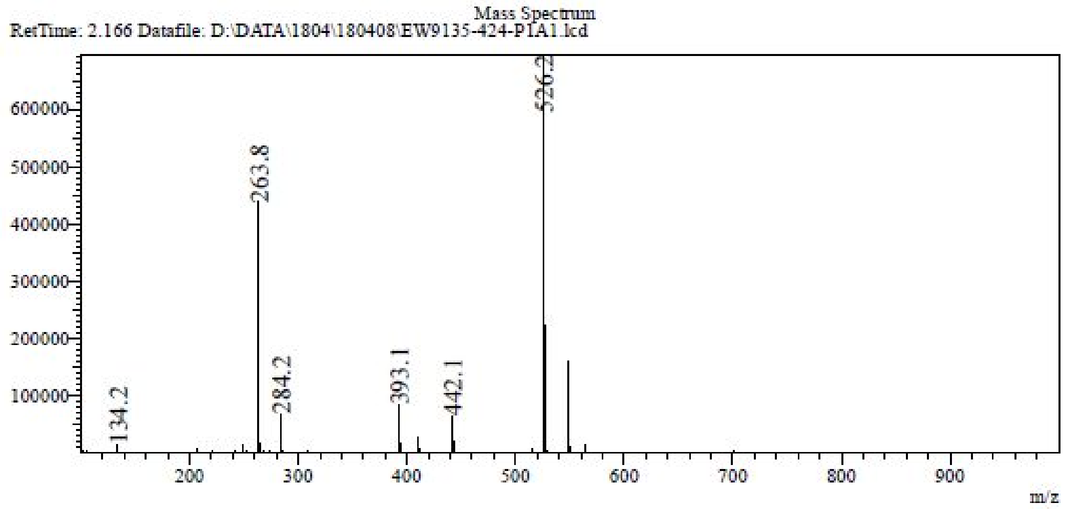

Mass Spectrum
RetTime: 2.166 Datafile: D:\DATA\1804\180408\EW9135-424-P1A1.lcd
600000
500000
400000
300000
200000
100000
526.2
263.8
284.2
393.1
442.1
134.2
200
300
400
500
600
700
800
900
m/z
